# Interventions to optimize embryo transfer in women undergoing assisted conception: a comprehensive systematic review and meta-analyses

**DOI:** 10.1093/humupd/dmac009

**Published:** 2022-03-24

**Authors:** Bede Tyler, Hugo Walford, Jennifer Tamblyn, Stephen D Keay, Dimitrios Mavrelos, Ephia Yasmin, Bassel H Al Wattar

**Affiliations:** UCL Institute for Women's Health, University College London, London, UK; UCL Institute for Women's Health, University College London, London, UK; Institute of Metabolism and Systems Research (IMSR), University of Birmingham, Birmingham, UK; Centre for Reproductive Medicine, University Hospital of Coventry & Warwickshire, Coventry, UK; UCL Institute for Women's Health, University College London, London, UK; Reproductive Medicine Unit, Elizabeth Garrett Anderson Wing, University College London Hospitals, London, UK; UCL Institute for Women's Health, University College London, London, UK; Reproductive Medicine Unit, Elizabeth Garrett Anderson Wing, University College London Hospitals, London, UK; UCL Institute for Women's Health, University College London, London, UK; Reproductive Medicine Unit, Elizabeth Garrett Anderson Wing, University College London Hospitals, London, UK

**Keywords:** embryo transfer, assisted conception, IVF, ICSI, systematic review, meta-analysis

## Abstract

**BACKGROUND:**

Several interventions and techniques are suggested to improve the outcome of embryo transfer (ET) in assisted conception. However, there remains no consensus on the optimal practice, with high variations among fertility specialists.

**OBJECTIVE AND RATIONALE:**

We conducted a comprehensive systematic review and meta-analyses of randomized controlled trials (RCTs) aiming to identify effective interventions that could be introduced around the time of ET to improve reproductive outcomes.

**SEARCH METHODS:**

We searched the electronic databases (MEDLINE, EMBASE and Cochrane CENTRAL) from inception until March 2021 using a multi-stage search strategy of MeSH terms and keywords, and included all RCTs that evaluated an intervention in the 24-h period before/after ET in women undergoing IVF/ICSI. Our primary outcome was clinical pregnancy rate post-ET confirmed as viable pregnancy on ultrasound scan. We assessed the risk of bias in included trials and extracted data in duplicate. We pooled data using a random-effect meta-analysis and reported using risk ratio (RR) with 95% CI. We explored publication bias and effect modifiers using subgroup analyses.

**OUTCOMES:**

Our search yielded 3685 citations of which we included 188 RCTs (38 interventions, 59 530 participants) with a median sample size of 200 (range 26–1761). The quality of included RCTs was moderate with most showing a low risk of bias for randomization (118/188, 62.8%) and attrition (105/188, 55.8%) but there was a significant risk of publication bias (Egger’s test *P* = 0.001). Performing ET with ultrasound guidance versus clinical touch (n = 24, RR 1.265, 95% CI 1.151–1.391, *I*^2^ = 38.53%), hyaluronic acid versus routine care (n = 9, RR 1.457, 95% CI 1.197–1.261, *I*^2^ = 46.48%) and the use of a soft versus hard catheter (n = 27, RR 1.122, 95% CI 1.028–1.224, *I*^2^ = 57.66%) led to higher clinical pregnancy rates. Other pharmacological add-ons also showed a beneficial effect including granulocyte colony-stimulating factor (G-CSF: n = 4, RR 1.774, 95% CI 1.252–2.512, *I*^2^ = 0), Atosiban (n = 7, RR 1.493, 95% CI 1.184–1.882, *I*^2^ = 68.27%) and hCG (n = 17, RR 1.232, 95% CI 1.099–1.382, *I*^2^ = 57.76%). Bed rest following ET was associated with a reduction in clinical pregnancy (n = 6, RR 0.857, 95% CI 0.741–0.991, *I*^2^ = 0.01%). Other commonly used interventions, such as non-steroidal anti-inflammatory drugs, prophylactic antibiotics, acupuncture and cervical mucus removal, did not show a significant benefit on reproductive outcomes. Our effect estimates for other important outcomes, including miscarriage and live birth, were limited by the varied reporting across included RCTs.

**WIDER IMPLICATIONS:**

Using ultrasound guidance, soft catheters and hyaluronic acid at the time of ET appears to increase clinical pregnancy rates. The use of Atosiban, G-CSF and hCG showed a trend towards increased clinical pregnancy rate, but larger trials are required before adopting these interventions in clinical practice. Bed rest post-ET was associated with a reduction in clinical pregnancy and should not be recommended.

## Introduction

Rapid developments in ART offered hope to many subfertile couples, with more than 8 million ART babies conceived worldwide (Steptoe and Edwards, 1978; Zegers-Hochschild *et al.*, 2009; Cardona Barberán et al., 2020). ART treatments are complex, comprising several interrelated steps often with high stress and psychological bearings on couples under treatment (Malina and Pooley, 2017). Numerous interventions or ‘add-on’ therapies have been introduced over the last few decades to optimize the outcome of ART treatments and assisted conception rates (Farquhar, 2019). However, the effectiveness and safety of several add-ons remain unclear raising concerns about patients’ safety and the vulnerability to profiteering (Galiano *et al.*, 2021).

Embryo transfer (ET) is a crucial component to the success of ART treatments (Mains and van Voorhis, 2010). While seemingly a simple procedure, ET is operator dependent with suboptimal practice often linked to cycle failure and reduced pregnancy rates (Yao *et al.*, 2009a). Some add-ons are proposed at the time of ET to increase the chances of conception, such as the use of ultrasound guidance (Cozzolino *et al.*, 2018), pharmacological interventions aimed to minimize uterine contractility at the time of the transfer (Ng *et al.*, 2019; Schwarze *et al.*, 2020) and also pre-transfer acupuncture and relaxation techniques (Smith *et al.*, 2019). Currently, the practice of ET varies among fertility specialists with no consensus on the optimal ET technique (Nancarrow *et al.*, 2021).

The American Society for Reproductive Medicine (ASRM) guideline recommends several ET techniques and add-ons including the use of ultrasound guidance, soft catheters and avoidance of bed rest (Penzias *et al.*, 2017a). Since its publication in 2016, several new add-ons have been proposed, with more than 20 relevant randomized controlled trials (RCTs) reported. Contemporary evidence synthesis is, therefore, needed to inform and update the current knowledge gap on ET practice.

To address this research need, we conducted a comprehensive systematic review and meta-analyses of randomized trials evaluating any intervention introduced at the time of ET to improve reproductive outcomes in couples undergoing ART.

## Methods

We conducted a systematic review using a prospectively registered protocol (PROSPERO: CRD42020216199) and reported in line with established guidelines (Page *et al.*, 2021).

### Search strategy

We searched the electronic databases (MEDLINE, EMBASE and Cochrane CENTRAL) from inception until March 2021 for all RCTs that evaluated an intervention introduced at the time of ET in women undergoing ART treatment. We used a multi-stage search strategy using MeSH terms and keywords, and combined them using the Boolean operators AND/OR to identify relevant citations (Supplementary Data). We did not apply any search filters or language restrictions. We screened the bibliographies of relevant articles and performed complementary searches in Google Scholar and Scopus to identify any missed citations and grey literature.

### Review selection and inclusion process

Two authors (B.T. and H.W.) independently screened the titles and abstracts to identify relevant citations. Full-text articles were then screened against our inclusion criteria. Any discrepancies were resolved through consultation with the senior author (B.H.A.). We included all RCTs that evaluated any clinical intervention introduced at the time of ET (within 24 h of the procedure) following any ART treatment (IVF and/or ICSI) with the aim to improve implantation rate irrespective of the cause of subfertility. Interventions were defined as ‘add-ons’ if they were introduced as supplementary to a standardized ART protocol in both groups of comparisons in the 24-h period before/after ET. Comparison groups in included trials received the same ART protocol versus control with no additional treatment (standard care), placebo or sham treatment.

Studies reporting intra-uterine sperm injection or ovulation induction treatments were excluded. Articles not in English were translated and included if deemed relevant. We also excluded quasi, non-randomized, observational and animal studies. Review articles and RCTs that did not report on any reproductive outcomes were also excluded.

### Data extraction

Data extraction was performed in duplicate (B.T. and H.W.) using a piloted electronic data collection tool with the following characteristics collected: study publication year and journal, inclusion–exclusion criteria, type of intervention and comparison evaluated, characteristics of the included study population and the evaluated ART treatments and all reproductive outcomes.

Our primary outcome was clinical pregnancy rate post-ET confirmed as viable pregnancy on ultrasound scan. We also reported live birth, ongoing pregnancy (a viable pregnancy beyond 12 weeks of gestation) and on biochemical pregnancy (confirmed with a positive βhCG test).

### Quality assessment and confidence of evidence

The quality of published literature was assessed by two independent reviewers (B.T. and H.W.) using the Cochrane Risk of Bias assessment tool (ROB2; Sterne et al., 2019). Studies were assessed in five domains: randomization process (randomization bias), deviations from intended interventions (allocation bias), performance bias, missing outcome data (attrition bias), measurement of the outcome (detection bias) and selection of the reported result (reporting bias). Each domain was assessed to have a high, medium or low risk of bias. Due to the nature of the intervention, we did not penalize unblinded trials but reported on assessors blinding. In case of uncertainty, consensus was established with input from a third reviewer (J.T.).

We employed the Grading of Recommendations Assessment, Development and Evaluation (GRADE) approach (GRADE Working Group, 2004) to evaluate the confidence in available evidence for each intervention and aid translation into clinical practice. Two independent reviewers (H.W. and J.T.) assessed the certainty of the evidence for the following domains: risk of bias in primary studies for each intervention, inconsistency across included trials, indirectness or imprecision in the pooled effect estimates. A final overall assessment was made for each intervention as high, moderate, low or very low in consultation with a third reviewer (B.H.A.).

### Statistical analysis

We reported on dichotomous outcomes using summary risk ratio (RR) with 95% CI and continuous outcomes using weighted mean difference. We pooled data using a random-effect meta-analysis and applied a restricted maximum likelihood model (Lin *et al.*, 2013). Study heterogeneity among included trials was assessed using the *I*^2^ statistics. We also assessed the publication bias and small study effect using a funnel plot for each pairwise comparison and performed Egger’s test to assess its statistical significance (Sterne and Harbord, 2004). We planned a sensitivity meta-regression and subgroup analyses to investigate potential effect modifiers where relevant (Harbord and Higgins, 2009). All statistical analyses were conducted in Stata V13 (StataCorp, TX, USA) and Open Meta-analyst software (Brown University; Providence, RI, USA).

## Results

**Figure 1.**
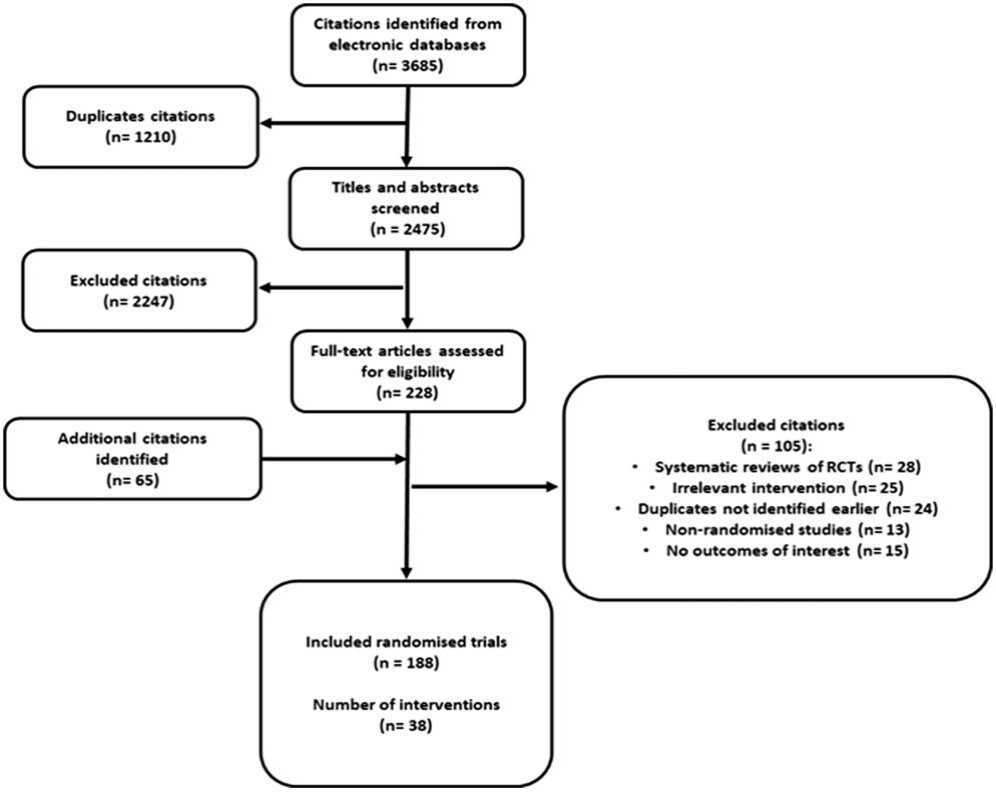
**Selection and inclusion process for randomized controlled trials evaluating interventions at the time of embryo transfer in women undergoing assisted reproduction**.

Our electronic search identified 3685 titles and abstracts of which we screened 228 articles in full against our inclusion criteria. We identified a further 65 additional records from screening bibliographies and supplementary searches. In total, we included 188 RCTs reporting on 59 530 participants (Fig. 1). The median sample size across the studies was 200 (range 26–1761). About half of the included studies were conducted in European countries (62/188, 33%) followed by the USA (23/188, 12.2%) and Iran (17/188, 9%). More than a third of included studies performed an ET at Day 2–3 (cleavage stage; 76/188, 40.4%), 8.5% at Day 5–6 (blastocyst; 16/188, 8.5%) and a third did not specify (69/188, 36.7%). Most studies performed a fresh ET (96/188, 51.1%), about a 10th performed either a frozen or fresh ET (21/188, 11.2%; Supplementary Tables SI and SII).

### Quality assessment

**Figure 2.**
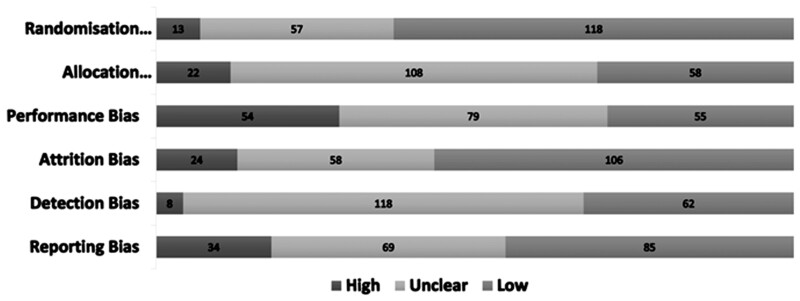
**Risk of bias in included randomized controlled trials evaluating interventions at the time of embryo transfer in women undergoing assisted reproduction**.

Overall, the quality of included RCTs was moderate with most studies assessed as low risk of bias for randomization (118/188, 62.8%) and attrition (105/188, 56.4%). Almost half of the studies were at low risk of bias for outcome reporting (85/188, 45.2%) and one-third were low risk for allocation (58/188, 30.9%) and detection bias (62/188 33.0%). Performance bias was judged to be high in one-third of the included studies (54/188, 28.7%; Fig. 2).

**Figure 3.**
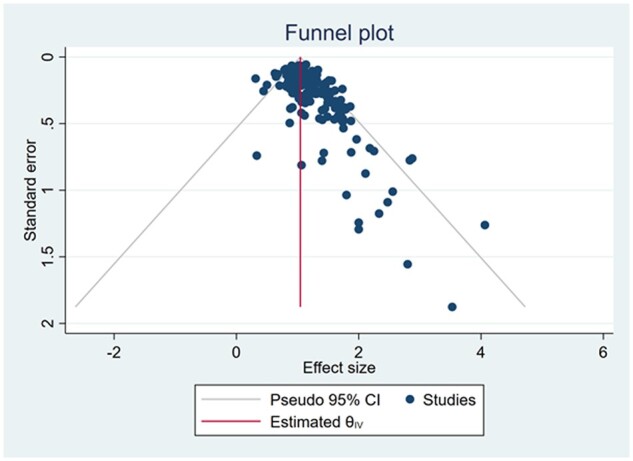
**Funnel plot evaluating the risk of publication bias in randomized controlled trials evaluating interventions at the time of embryo transfer in women undergoing assisted reproduction**.

Our funnel plots showed significant variations in effect size and standard error across included RCTs with evidence of small study effect (Fig. 3). We explored this further by removing interventions that were evaluated by a single RCT, which reduced the distribution of the funnel plot (Supplementary Fig. S1). Egger’s test showed significant publication bias with a *P*-value of 0.001.

### Pharmacological interventions

#### Antibiotics

We identified two RCTs that evaluated the effects of using antibiotics at the time of ET (Peikrishvili *et al.*, 2004; Brook *et al.*, 2006; n = 625 participants). The pooled effect estimate showed no significant benefit for the use of antibiotics compared to standard care on the rate of clinical pregnancy (RR 1.008, 95% CI 0.812–1.251, *I*^2^ = 0; Supplementary Fig. S2a). Ongoing pregnancy and miscarriage were reported only in one study (Peikrishvili *et al.*, 2004) showing no significant benefit (RR 0.840, 95% CI 0.570–1.239 and RR 1.255, 95% CI 0.499–3.157, respectively).

#### Atosiban

Seven RCTs assessed the effectiveness of Atosiban to promote uterine relaxation at the time of ET (Ahn *et al.*, 2009; Moraloglu *et al.*, 2010; Song *et al.*, 2013; Ng *et al.*, 2014; He *et al.*, 2016; Hebisha *et al.*, 2016; Yuan *et al.*, 2019) using varied doses and preparations (n = 1646 participants). The pooled effect estimate showed a significant increase in clinical pregnancy with the use of Atosiban compared to placebo (RR 1.493, 95% CI 1.184–1.882, *I*^2^ = 68.27%; Supplementary Fig. S3a).

Ongoing pregnancy was shown to increase with the use of Atosiban in three RCTs (RR 1.137, 95% CI 1.007, 1.283, *P* = 0.435, *I*^2^ = 0%; Ng *et al.*, 2014; Visnova *et al.*, 2018; Bosch *et al.*, 2019) while there was no significant effect on live birth (n = 1, RR 1.05, 95% CI 0.879–1.240; Ng *et al.*, 2014), miscarriage (n = 5, RR 1.080, 95% CI 0.748–1.561, *I*^2^ = 0%; 24, 26–29) or biochemical pregnancy (n = 2, RR 1.741, 95% CI 0.664–4.567, *I*^2^ = 92.92%; 27, 29; Supplementary Fig. S3b–d).

A subgroup analysis showed a persistent improvement in clinical pregnancy associated with the use of a medium dose Atosiban (35–50 mg; n = 5, RR 1.591, 95% CI 1.110–1.714, *I*^2^ = 76.68%; Ahn *et al.*, 2009; Moraloglu *et al.*, 2010; Song *et al.*, 2013; Ng *et al.*, 2014; Yuan *et al.*, 2019) and as well as a low dose (5–10 mg; n = 2, RR 1.380, 95% CI 1.110–1.714, *I*^2^ = 68.27%; He *et al.*, 2016; Hebisha *et al.*, 2016; Supplementary Fig. S3e).

#### Nifedipine

Only one study with low risk of bias evaluated the use of Nifedipine for uterine relaxation at the time of ET (Ng *et al.*, 2019). There was no improvement in clinical pregnancy (RR 1.115, 95% CI 0.548–2.267), live birth (RR 1.115, 95% CI 0.548–2.267) or miscarriage (RR 1.022, 95% CI 0.105–6.951) with using Nifedipine compared to placebo.

#### Non-steroidal anti-inflammatory drugs

We identified seven studies that evaluated the use of different non-steroidal anti-inflammatory drugs (NSAIDs) at the time of ET, including Piroxicam (n = 4; Moon *et al.*, 2004; Dehghani Firouzabadi *et al.*, 2006; Prato and Borini, 2009; Zarei *et al.*, 2021), Indomethacin (n = 1; Bernabeu *et al.*, 2006), Aspirin (n = 1; Duvan *et al.*, 2006) and Ibuprofen (n = 1; Fekih *et al.*, 2013). A pooled effect estimates for all types of NSAIDs (n = 1207 participants) showed no significant effect on clinical pregnancy (RR 1.294, 95% CI 0.973–1.721, *I*^2^ = 63.92%; Supplementary Fig. S4a), miscarriage (n = 4, RR 0.787, 95% CI 0.391–1.582, *I*^2^ = 0%; Dehghani Firouzabadi *et al.*, 2006; Prato and Borini, 2009; Fekih *et al.*, 2013; Zarei *et al.*, 2021) and biochemical pregnancy (n = 5, RR 1.032, 95% CI 0.840–1.268, *I*^2^ = 26.69%; Bernabeu *et al.*, 2006; Dehghani Firouzabadi *et al.*, 2006; Duvan *et al.*, 2006; Prato and Borini, 2009; Zarei *et al.*, 2021; Supplementary Fig. S4b and c). Subgroup meta-analyses per type of NSAIDs showed similar results with no overall benefit (Supplementary Fig. S4d).

#### Steroids

Only one study evaluated the effect of Prednisone on reproductive outcomes at the time of ET (Duvan *et al.*, 2006) which had no significant effect on clinical pregnancy (RR 1.064, 95% CI 0.484–2.337).

#### hCG

Supplementing hCG at the time of ET was the most commonly evaluated pharmacological intervention in 17 RCTs, given as an intrauterine infusion with or without embryo culture media (Mansour *et al.*, 2011; Cambiaghi *et al.*, 2013; Leao *et al.*, 2013; HoNg *et al.*, 2014; Santibañez *et al.*, 2014; Singh and Singh, 2014; Zarei *et al.*, 2014; Aaleyasin *et al.*, 2015; Wirleitner *et al.*, 2015a,b; Dehghani Firouzabadi et al., 2016; Eskandar *et al.*, 2016; Hosseini *et al.*, 2016; Mostajeran *et al.*, 2017; Hafezi *et al.*, 2018; Laokirkkiat *et al.*, 2019; WaNg *et al.*, 2019; Supplementary Fig. S5a). The pooled data showed a significant increase in clinical pregnancy with hCG use (n = 17, RR 1.232, 95% CI 1.099–1.382, *I*^2^ = 57.76%).

We pooled data from nine RCTs that evaluated the provision of intrauterine hCG compared to standard care, which showed a significant improvement in clinical pregnancy with hCG use (n = 9, RR 1.269, 95% CI 1.092–1.474, *I*^2^ = 43.92%; Mansour *et al.*, 2011; Cambiaghi *et al.*, 2013; Leao *et al.*, 2013; Santibañez *et al.*, 2014; Dehghani Firouzabadi et al., 2016; Eskandar *et al.*, 2016; Hosseini *et al.*, 2016; Mostajeran *et al.*, 2017; WaNg *et al.*, 2019). Eight of the included studies compared hCG injections to a controlled intrauterine injection of embryo culture medium, which did not show a significant improvement in clinical pregnancy rates (n = 8, 1.193, 95% CI 0.996–1.428, *I*^2^ = 69.7%; HoNg *et al.*, 2014; Singh and Singh, 2014; Zarei *et al.*, 2014; Aaleyasin *et al.*, 2015; Wirleitner *et al.*, 2015a,b; Hafezi *et al.*, 2018; Laokirkkiat *et al.*, 2019; Supplementary Fig. S5g).

Overall, hCG supplementation did not significantly increase live birth (n = 7, RR 1.132, 95% CI 0.924–1.387, *I*^2^ = 73.37%; Supplementary Fig. S5e; Mansour *et al.*, 2011; Singh and Singh, 2014; Aaleyasin *et al.*, 2015; Wirleitner *et al.*, 2015a,b; Hafezi *et al.*, 2018; Laokirkkiat *et al.*, 2019), ongoing pregnancy (n = 2, RR 1.339, 95% CI 0.947–1.893, *I*^2^ = 78.24%; Supplementary Fig. S5c; HoNg *et al.*, 2014; Aaleyasin *et al.*, 2015), or miscarriage rates (n = 9, RR 1.092, 95% CI 0.846–1.408, *I*^2^ = 0%; Supplementary Fig. S5d; Mansour *et al.*, 2011; HoNg *et al.*, 2014; Singh and Singh, 2014; Zarei *et al.*, 2014; Aaleyasin *et al.*, 2015; Wirleitner *et al.*, 2015a,b; Dehghani Firouzabadi et al., 2016; Hosseini *et al.*, 2016; Hafezi *et al.*, 2018). However, there was a significant increase in biochemical pregnancy rate (n = 5, RR 1.241, 95% CI 1.012–1.521, *I*^2^ = 70.33%; Supplementary Fig. S5b; Santibañez *et al.*, 2014; Aaleyasin *et al.*, 2015; Wirleitner *et al.*, 2015a; Hafezi *et al.*, 2018; Laokirkkiat *et al.*, 2019). Several hCG doses were used ranging from 100 to 6500 IU (Supplementary Table SI). However, the most commonly used dose was 500 IU (dissolved in embryo culture medium) evaluated in 15 studies, which showed a consistent increase in clinical pregnancy rates (n = 15, RR 1.275, 95% CI 1.119, 1.4521, *I*^2^ = 60.05%; Supplementary Fig. S5f; Mansour *et al.*, 2011; Cambiaghi *et al.*, 2013; Leao *et al.*, 2013; HoNg *et al.*, 2014; Santibañez *et al.*, 2014; Singh and Singh, 2014; Aaleyasin *et al.*, 2015; Wirleitner *et al.*, 2015a,b; Dehghani Firouzabadi et al., 2016; Eskandar *et al.*, 2016; Hosseini *et al.*, 2016; Hafezi *et al.*, 2018; Laokirkkiat *et al.*, 2019; WaNg *et al.*, 2019).

#### Hyaluronic acid

**Figure 4.**
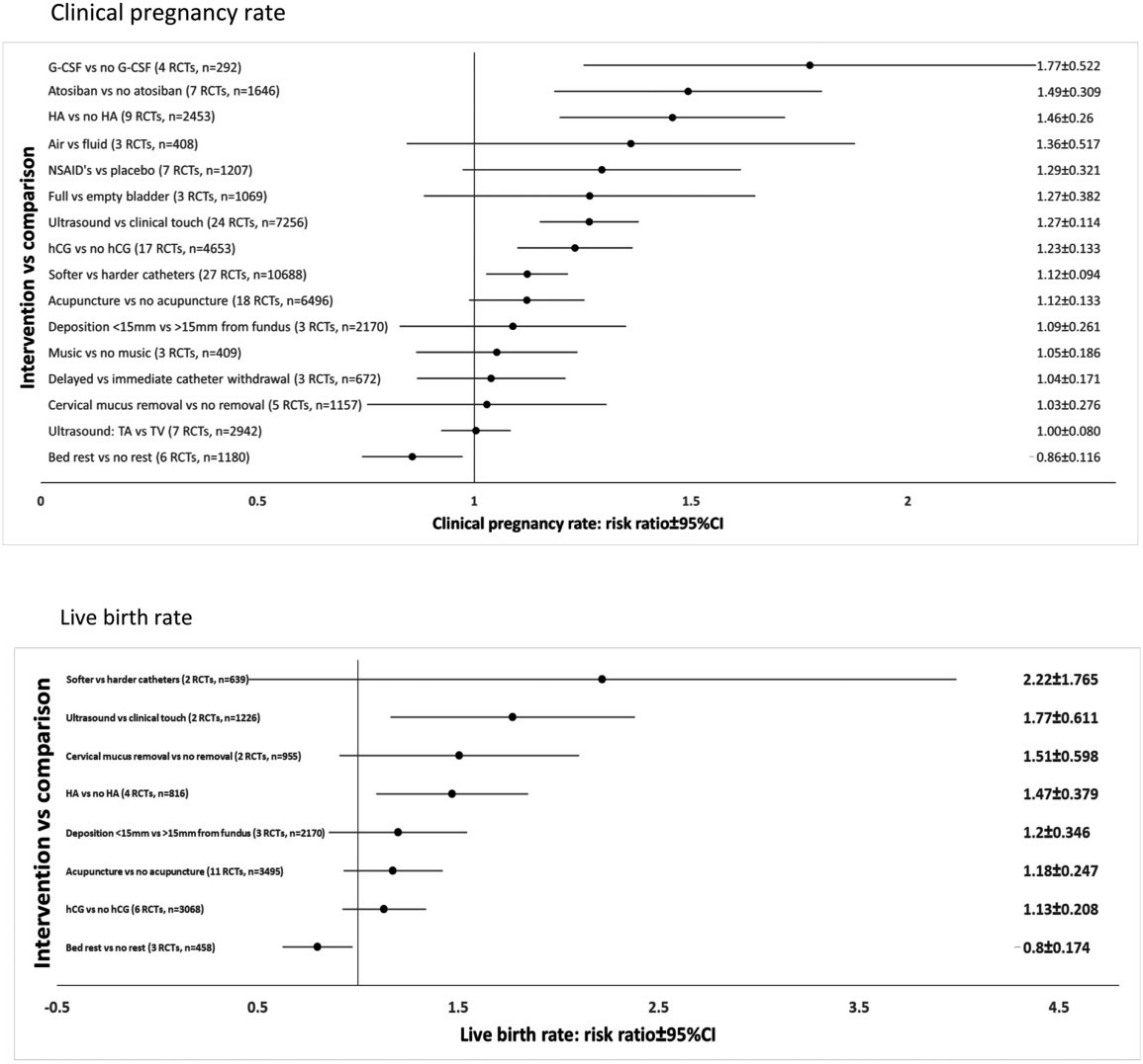
**Summary forest plots of effect estimates of evaluated interventions at the time of embryo transfer on the reproductive outcomes in women undergoing assisted reproduction.** G-CSF, granulocyte colony-stimulating factor; TA, transabdominal; TV, transvaginal; CT, computerized tomography; NSAID, non-steroidal anti-inflammatory drug; RCT, randomized controlled trial; HA, hyaluronic acid.

In total, nine RCTs evaluated the use of hyaluronic acid (HA) at the time of ET compared to no intervention/placebo (Feichtinger *et al.*, 1992; Simon *et al.*, 2003; Friedler *et al.*, 2005, 2007; Korosec *et al.*, 2007; Mahani and Davar, 2007; Tomari *et al.*, 2014; Fasano *et al.*, 2016; Perez *et al.*, 2019). The random-effect meta-analysis showed a significant increase in clinical pregnancy with the administration of HA versus placebo (RR 1.457, 95% CI 1.197–1.261, *I*^2^ = 46.48%; Fig. 4a;Feichtinger *et al.*, 1992; Simon *et al.*, 2003; Friedler *et al.*, 2005, 2007; Korosec *et al.*, 2007; Mahani and Davar, 2007; Tomari *et al.*, 2014; Fasano *et al.*, 2016; Perez *et al.*, 2019). Compared to no intervention, HA was seen to significantly increase live birth rate (n = 4, RR 1.471, 95% CI 1.092–1.982, *I*^2^ = 4.89%; Fig. 4b;Supplementary Fig. S6c; Simon *et al.*, 2003; Korosec *et al.*, 2007; Kandari, 2019; Perez *et al.*, 2019), but did not affect ongoing pregnancy rate (n = 4, RR 1.170, 95% CI 0.894–1.532, *I*^2^ = 0.01%; Supplementary Fig. S6b; Feichtinger *et al.*, 1992; Simon *et al.*, 2003; Friedler *et al.*, 2007; Korosec *et al.*, 2007).

Twelve RCTs compared the use of a high- versus low-dose HA at the time of ET (Schoolcraft *et al.*, 2002; Balaban *et al.*, 2004; Yakin *et al.*, 2004; Ravhon *et al.*, 2005; Walker *et al.*, 2005; Valojerdi *et al.*, 2006; Morbeck, 2007; Urman *et al.*, 2008; Dittmann-Műller *et al.*, 2009; Fancsovits *et al.*, 2011, 2015; Yung *et al.*, 2021), which was associated with a mild increase in clinical pregnancy (RR 1.097, 95% CI 1.028–1.169, *I*^2^ = 0.00%; Supplementary Fig. S7a; Schoolcraft *et al.*, 2002; Balaban *et al.*, 2004; Yakin *et al.*, 2004; Ravhon *et al.*, 2005; Walker *et al.*, 2005; Valojerdi *et al.*, 2006; Morbeck, 2007; Urman *et al.*, 2008; Dittmann-Műller *et al.*, 2009; Fancsovits *et al.*, 2011, 2015; Yung *et al.*, 2021), but had no significant effect on other reproductive outcomes (ongoing pregnancy rate (n = 2, RR 0.918, 95% CI 0.714–1.179, *I*^2^ = 3.63%; Morbeck, 2007; Yung *et al.*, 2021) and live birth rate (n = 6, RR 1.134, 95% CI 1.000–1.285, *I*^2^ = 22.17%; Valojerdi *et al.*, 2006; Morbeck, 2007; Urman *et al.*, 2008; Fancsovits *et al.*, 2011, 2015; Yung *et al.*, 2021; Supplementary Fig. S7b, c)).

#### Granulocyte colony-stimulating factor

Our meta-analysis of four RCTs evaluating the effects of using granulocyte colony-stimulating factor (G-CSF) at the time of ET compared to placebo (Singh *et al.*, 2015; Obidniak *et al.*, 2016; Arefi *et al.*, 2018; Singh and Singh, 2018) showed a significant increase in clinical pregnancy (RR 1.774, 95% CI 1.252–2.512, *I*^2^ = 0; Fig. 4a, Supplementary Fig. S8). Only one RCT reported on each of live birth (RR 1.518, 95% CI 0.769–2.997; Arefi *et al.*, 2018), miscarriage (RR 1.272, 95% CI 0.123–13.177; Arefi *et al.*, 2018) and ongoing pregnancy (RR 2.473, 95% CI 1.131–5.405; Singh and Singh, 2018), all showing mixed benefit of using G-CSF with low evidence quality (Supplementary Table SIV).

#### Seminal fluid

The use of seminal fluid was assessed in two RCTs (Aflatoonian et al., 2009; Karimian *et al.*, 2010) of medium risk of bias (Supplementary Table SIII). There was no significant benefit to improve biochemical pregnancy (n = 2, RR 1.138, 95% CI 0.912, 1.420, *I*^2^ = 0%; Supplementary Fig. S9), clinical pregnancy (RR 1.205, 95% CI 0.717–2.025) or live birth (RR 1.281, 95% CI 0.908–1.808; Aflatoonian *et al.*, n.d.; Karimian *et al.*, 2010).

#### Culture medium

One RCT (Sigalos *et al.*, 2018) evaluated the impact of ET low versus high volumes of culture medium, which showed no benefit on clinical pregnancy (RR 0.862, 95% CI 0.662–1.125) or ongoing pregnancy (RR 0.833, 95% CI 0.586–1.185).

#### Plasma infusion

The study by Obidniak *et al.* (2017) evaluated the effects of plasma infusion at the time of ET (n = 90 participants) reporting a 2-fold increase in clinical pregnancy compared to placebo (RR 2.182, 95% CI 1.219–3.904). Confidence in this evidence was judged to be low (Supplementary Table SIII).

#### 17-Hydroxyprogesterone caproate

We identified one RCT (Abu-Musa et al., 2008) that evaluated the effectiveness of providing one injection of 17-hydroxyprogesterone caproate (17-HPC) compared to placebo at the time of ET (n = 125 participants). There was no significant effect on clinical pregnancy with 17-HPC injection versus placebo (RR 0.902, 95% CI 0.569, 1.429).

#### Powdered gloves

A single RCT evaluated the difference in using powdered versus unpowdered gloves at the time of ET (n = 712 participants; Hannoun *et al.*, 2009). There was no difference in clinical pregnancy rate across both groups (RR 1.008, 95% CI 0.834–1.218).

### ET techniques

#### Use of ultrasound

A large body of evidence was captured evaluating the different ultrasound modalities used at the time of ET. We pooled data from 24 RCTs that compared ET under ultrasound guidance to ET guided by clinical touch, which showed a significant increase in clinical pregnancy (n = 24, RR1.265, 95% CI 1.151–1.391, *I*^2^ = 8.53%; Fig. 4a;Wisanto *et al.*, 1989; Prapas *et al.*, 1995; Kan *et al.*, 1999; Coroleu *et al.*, 2000, 2002b; Abdelmassih *et al.*, 2001; Garcı’a-Velasco *et al.*, 2001, 2002; Tang *et al.*, 2001; Matorras *et al.*, 2002; Sallam *et al.*, 2002; Bar-Hava *et al.*, 2003; Marconi *et al.*, 2003; Weissman *et al.*, 2003; de Camargo Martins *et al.*, 2004; Moraga-Sanchez *et al.*, 2004; Li *et al.*, 2005; Maldonado *et al.*, 2005; Chen *et al.*, 2007; Davar *et al.*, 2007; Kosmas *et al.*, 2007; Drakeley *et al.*, 2008; Eskandar *et al.*, 2008; Ammar *et al.*, 2013), ongoing pregnancy (n = 8, RR 1.369, 95% CI 1.144–1.639, *I*^2^ = 51.34% (Supplementary Fig. S10c; Coroleu *et al.*, 2000; Tang *et al.*, 2001; Matorras *et al.*, 2002; Marconi *et al.*, 2003; Davar *et al.*, 2007; Drakeley *et al.*, 2008; Eskandar *et al.*, 2008; Ammar *et al.*, 2013), biochemical pregnancy (n = 2, RR 1.496, 95 CI: 1.174–1.906; Supplementary Fig. S10b; Coroleu *et al.*, 2000; Kosmas *et al.*, 2007), and live birth (n = 2, RR 1.744, 95% CI 1.163–2.706, *I*^2^ = 79.97%; Fig. 4b;Supplementary Fig. S10e; Eskandar *et al.*, 2008; Azmy *et al.*, 2009) with the use of ultrasound. There was no difference in miscarriage rates between both groups (n = 12, RR 1.145, 95% CI 0.883–1.484, *I*^2^ = 0%; Supplementary Fig. S10d; Garcı’a-Velasco *et al.*, 2001, 2002; Tang *et al.*, 2001; Coroleu *et al.*, 2002b; Matorras *et al.*, 2002; Weissman *et al.*, 2003; de Camargo Martins *et al.*, 2004; Davar *et al.*, 2007; Kosmas *et al.*, 2007; Eskandar *et al.*, 2008; Azmy *et al.*, 2009; Ammar *et al.*, 2013).

We included seven RCTs that compared the use of transabdominal versus transvaginal ultrasound at the time of ET (Porat *et al.*, 2010; Bodri *et al.*, 2011; Deep *et al.*, 2013; Hauzman *et al.*, 2013; Dalal *et al.*, 2014; Revelli *et al.*, 2016; Karavani *et al.*, 2017). There was no difference between both groups on clinical pregnancy (n = 7, RR 1.004, 95% CI 0.924–1.090, *I*^2^ = 0%; Supplementary Fig. S10f; Porat *et al.*, 2010; Bodri *et al.*, 2011; Deep *et al.*, 2013; Hauzman *et al.*, 2013; Dalal *et al.*, 2014; Revelli *et al.*, 2016; Karavani *et al.*, 2017), ongoing pregnancy (n = 5, RR 1.029, 95% CI 0.922–1.148, *I*^2^ = 0.01%; Supplementary Fig. S10h; Porat *et al.*, 2010; Bodri *et al.*, 2011; Hauzman *et al.*, 2013; Revelli *et al.*, 2016; Karavani *et al.*, 2017), biochemical pregnancy (n = 2, RR 0.977, 95% CI 0.712–1.341; Supplementary Fig. S10g; Bodri *et al.*, 2011; Hauzman *et al.*, 2013), miscarriage (n = 3, RR 0.835, 95% CI 0.575–1.211, *I*^2^ = 0%; Supplementary Fig. S10i; Bodri *et al.*, 2011; Revelli *et al.*, 2016; Karavani *et al.*, 2017) and live birth (n = 1, RR 0.789, 95% CI 0.444–1.403; Karavani *et al.*, 2017). One RCT by Saravelos *et al.* (2016) evaluated the use of 3-dimensional versus 2-dimensional ultrasound at the time of ET (n = 474 participants), which showed no difference for clinical pregnancy (RR 0.981, 95% CI 0.800–1.202) or any of the other secondary outcomes.

#### Bladder fullness

Three RCTs compared the difference in reproductive outcomes following ET on a full versus empty bladder (n = 1069 participants; Mitchell *et al.*, 1989; Lewin *et al.*, 1997; Lorusso *et al.*, 2005). Overall, there was no difference across both groups for clinical pregnancy (n = 3, RR 1.266, 95% CI 0.884–1.813, *I*^2^ = 50.68%; Supplementary Fig. S11a; Mitchell *et al.*, 1989; Lewin *et al.*, 1997; Lorusso *et al.*, 2005), ongoing pregnancy (n = 2, RR 1.276, 95% CI 0.874–1.863; Supplementary Fig. S11b; Lewin *et al.*, 1997; Lorusso *et al.*, 2005) or miscarriage (n = 1, RR 1.047, 95% CI 0.467–2.346; Lorusso *et al.*, 2005).

#### Pressure on cervix

Two RCTs evaluated the effect of mechanical effect on the cervix by unscrewing the speculum to apply gentle pressure during ET (n = 716 participants), which showed no significant effect for clinical pregnancy (n = 2, RR 1.175, 95% CI 0.817–1.690; Supplementary Fig. S12; Mansour, 2005; Amui *et al.*, 2011) or ongoing pregnancy (n = 1, RR 0.843, 95% CI 0.485–1.468; Amui *et al.*, 2011).

#### Pump regulated transfer

One study (Caanen *et al.*, 2016) compared the use of a pump regulated transfer to manual ET (n = 599 participants), which showed no difference between methods for clinical pregnancy (RR 1.201, 95% CI 0.904–1.596) or ongoing pregnancy (RR 1.234, 95% CI 0.884–1.722).

#### Cervical mucus removal

Removing the cervical mucus before ET was assessed in six studies (Ruhlman *et al.*, 1999; Soroka *et al.*, 1999; Glass *et al.*, 2000; Berkkanoglu *et al.*, 2006; Visschers *et al.*, 2007; Moini *et al.*, 2011) compared to standard practice (n = 1157 participants) using different tools including cotton swabs (Moini *et al.*, 2011), cervical brush (Visschers *et al.*, 2007), aspirators (Ruhlman *et al.*, 1999; Soroka *et al.*, 1999) or flushing with culture media solution (Berkkanoglu *et al.*, 2006) or their combination (Glass *et al.*, 2000). Our meta-analysis showed no significant effect of cervical mucus removal on clinical pregnancy (n = 5, RR 1.029, 95% CI 0.753–1.405, *I*^2^ = 5.89%; Supplementary Fig. S13a; Ruhlman *et al.*, 1999; Soroka *et al.*, 1999; Glass *et al.*, 2000; Berkkanoglu *et al.*, 2006; Moini *et al.*, 2011), biochemical pregnancy (n = 2, RR 0.933, 95% CI 0.774–1.125; Supplementary Fig. S13b; Visschers *et al.*, 2007; Moini *et al.*, 2011), ongoing pregnancy (n = 2, RR 1.068, 95% CI 0.849–1.344; Supplementary Fig. S13c; Berkkanoglu *et al.*, 2006; Visschers *et al.*, 2007), miscarriage (n = 2, RR 0.790, 95% CI 0.487–1.282; Supplementary Fig. S13d; Visschers *et al.*, 2007; Moini *et al.*, 2011) or live birth (n = 2, RR 1.506, 95% CI 0.908–2.499; Supplementary Fig. S13e; Visschers *et al.*, 2007; Moini *et al.*, 2011).

#### ET catheters

A total of 27 RCTs compared ET outcomes using soft versus hard catheters (Gilberto Almodin *et al.*, n.d.; Wisanto *et al.*, 1989; Al-Shawaf *et al.*, 1993; Perin *et al.*, 1997; Grunert *et al.*, 1998; Amorcho *et al.*, 1999; Ghazzawi *et al.*, 1999; Mayer *et al.*, 1999; Boone *et al.*, 2001; Curfs *et al.*, 2001; Lavery *et al.*, 2001; Karande *et al.*, 2002; Levi-Setti *et al.*, 2002; McDonald and Norman, 2002; Mortimer *et al.*, 2002; van Weering *et al.*, 2002; Foutouh *et al.*, 2003; McIlveen *et al.*, 2005; Baris *et al.*, 2007; Rhodes *et al.*, 2007; El-Shawarby *et al.*, 2008; Saldeen *et al.*, 2008; Yao *et al.*, 2009b; Allahbadia *et al.*, 2010; Talwar *et al.*, 2011; Nihat Candan *et al.*, 2014). Overall, there was a significant increase in clinical pregnancy with the use of a soft catheter (RR 1.122, 95% CI 1.028–1.224, *I*^2^ = 57.66%; Fig. 4a;Supplementary Fig. S14a) but no difference was found for ongoing pregnancy (n = 3, RR 1.138, 95% CI 0.904–1.432, *I*^2^ = 32.46%; Supplementary Fig. S14b; Karande *et al.*, 2002; Levi-Setti *et al.*, 2002; Baris *et al.*, 2007) or live birth rates (n = 2, RR 2.222, 95% CI 0.457–10.806, *I*^2^ = 94.13%; Fig. 4b;Supplementary Fig. S14c; Perin *et al.*, 1997; Saldeen *et al.*, 2008).

Using a double compared to a single lumen ET catheter seemed to reduce clinical pregnancy rate in a small RCT of 66 participants (RR 0.314, 95% CI 0.129–0.764; Meriano *et al.*, 2000). Similarly, using a straight versus a bent tip catheter seemed to also reduce clinical pregnancy in another small RCT (Ocal *et al.*, 2003; RR 0.648, 95% CI 0.421–0.999). The use of an echogenic versus standard ET catheter did not affect the rate of clinical pregnancy in both groups (RR 1.302, 95% CI 0.966–1.756; Coroleu *et al.*, 2006). Confidence in evidence sought on these interventions was low (Table I, Supplementary Table SIV). Yayla Abide *et al.* (2018) suggested that catheter rotation at the time of ET compared to standard practice yielded a higher clinical pregnancy rate (RR 1.560, 95% CI 1.026–2.371) with no difference reported for ongoing pregnancy (RR 1.435, 95% CI 0.911–2.260) or biochemical pregnancy (RR 1.577, 95% CI 1.051–2.366). This RCT showed a moderate risk of bias and its results showed be interpreted with caution (Supplementary Table SIII).

**Table I:** GRADE assessment of evidence from randomised trials evaluating interventions at the time of embryo transfer in women undergoing assisted reproduction

Certainty assessment						Summary of findings		
Participants (studies) Follow up	Risk of bias	Inconsistency	Indirectness	Imprecision	Overall certainty of evidence	Study event rates (%)		Relative effect (95% CI)
						With Comparison	With Interventions at the time of ET	
***Higher confidence and significant effect size:***								
**Hyaluronic acid versus no HA**								
2453	not serious	not serious	not serious	not serious	⊕⊕⊕⊕	286/1305 (21.9%)	387/1148 (33.7%)	**RR 1.457**
(9 RCTs)					HIGH			(1.197 to 1.773)
**Ultrasound guided ET versus clinical touch**								
7256	not serious	not serious	not serious	serious ^a^	⊕⊕⊕◯	986/3587 (27.5%)	1245/3669 (33.9%)	**RR 1.265**
(24 RCTs)					MODERATE			(1.151 to 1.391)
**Softer versus harder catheters**								
10688	not serious	not serious	serious ^b^	not serious	⊕⊕⊕◯	1704/5248 (32.5%)	1969/5440 (36.2%)	**RR 1.122**
(27 RCTs)					MODERATE			(1.028 to 1.224)
***Lower confidence and significant effect size:***								
**G-CSF versus placebo/no G-CSF**								
292	serious ^c^	not serious	not serious	serious ^a^	⊕⊕◯◯	35/154 (22.7%)	58/138 (42.0%)	**RR 1.774**
(4 RCTs)					LOW			(1.252 to 2.512)
**Atosiban versus placebo/no atosiban**								
1646	not serious	serious ^d^	not serious	serious ^a^	⊕⊕◯◯	325/823 (39.5%)	426/823 (51.8%)	**RR 1.493**
(7 RCTs)					LOW			(1.184 to 1.882)
**hCG versus placebo/no hCG**								
4653	serious ^e^	serious ^f^	not serious	not serious	⊕⊕◯◯	883/2298 (38.4%)	1052/2355 (44.7%)	**RR 1.232**
(16 RCTs)					LOW			(1.099 to 1.382)
***High confidence and no significant effect:***								
**Transvaginal ultrasound versus transabdominal ultrasound**								
2942	not serious	not serious	not serious	not serious	⊕⊕⊕⊕	620/1457 (42.6%)	638/1485 (43.0%)	**RR 1.004**
(7 RCTs)					HIGH			(0.924 to 1.090)
***Some positive associations with clinical pregnancy rate:***								
**NSAID's versus placebo/no NSAID's**								
1207	not serious	not serious	serious ^h^	not serious	⊕⊕⊕◯	166/599 (27.7%)	222/608 (36.5%)	**RR 1.294**
(7 RCTs)					MODERATE			(0.973 to 1.721)
**Acupuncture versus placebo/no acupuncture**								
6496	not serious	serious ^i^	not serious	not serious	⊕⊕⊕◯	1379/3409 (40.5%)	1379/3087 (44.7%)	**RR 1.121**
(18 RCTs)					MODERATE			(0.988 to 1.273)
**Air versus fluid in catheter tip**								
408	serious ^c^	serious ^j^	not serious	serious ^a^	⊕◯◯◯	58/203 (28.6%)	76/205 (37.1%)	**RR 1.361**
(3 RCTs)					VERY LOW			(0.844 to 2.195)
**Full bladder versus empty bladder**								
1069	serious ^c^	serious ^k^	not serious	very serious ^g^	⊕◯◯◯	102/525 (19.4%)	148/544 (27.2%)	**RR 1.266**
(3 RCTs)					VERY LOW			(0.884 to 1.813)
***Lower confidence and no real associations with clinical pregnancy rate:***								
**Music versus no music**								
409	not serious	not serious	not serious	very serious ^g^	⊕⊕◯◯	101/209 (48.3%)	102/200 (51.0%)	**RR 1.052**
(3 RCTs)					LOW			(0.866 to 1.279)
**Nurse versus doctor**								
655	not serious	not serious	not serious	very serious ^g^	⊕⊕◯◯	115/327 (35.2%)	111/328 (33.8%)	**RR 0.961**
(2 RCTs)					LOW			(0.778 to 1.187)
**Pressure on cervix versus no pressure**								
716	serious ^c^	not serious	not serious	very serious ^g^	⊕◯◯◯	172/359 (47.9%)	221/357 (61.9%)	**RR 1.175**
(2 RCTs)					VERY LOW			(0.817 to 1.690)
**Site of deposition: further from fundus (15-20mm) versus closer (<12mm)**								
2170	serious ^c^	serious ^l^	very serious ^m^	very serious ^g^	⊕◯◯◯	176/921 (19.1%)	256/1249 (20.5%)	**RR 1.089**
(3 RCTs)					VERY LOW			(0.828 to 1.431)
**Delayed versus immediate catheter withdrawal**								
672	serious ^c^	not serious	not serious	very serious ^g^	⊕◯◯◯	129/339 (38.1%)	128/333 (38.4%)	**RR 1.039**
(3 RCTs)					VERY LOW			(0.868 to 1.243)
**Cervical mucus removal versus no removal**								
1157	serious ^c^	serious ^o^	not serious	very serious ^g^	⊕◯◯◯	225/597 (37.7%)	229/560 (40.9%)	**RR 1.029**
(5 RCTs)					VERY LOW			(0.753 to 1.405)
**Antibiotics versus no antibiotics**								
625	serious ^c^	not serious	serious ^p^	very serious ^g^	⊕◯◯◯	109/317 (34.4%)	107/308 (34.7%)	**RR 1.008**
(2 RCTs)					VERY LOW			(0.812 to 1.251)
**Mindfulness versus no mindfulness**								
500	serious ^c^	not serious	not serious	very serious ^g^	⊕◯◯◯	121/247 (49.0%)	113/253 (44.7%)	**RR 0.911**
(2 RCTs)					VERY LOW			(0.755 to 1.098)
***Significant negative association with clinical pregnancy rates:***								
**Bed rest versus shorter/no bed rest**								
1180	serious ^c^	not serious	not serious	serious ^a^	⊕⊕◯◯	217/591 (36.7%)	185/589 (31.4%)	**RR 0.857**
(6 RCTs)					LOW			(0.741 to 0.991)

The table presents evidence summary for interventions evaluated in two or more randomised trials.

GRADE: Grading of Recommendations Assessment, Development and Evaluation, CI: Confidence interval; RR: Risk ratio, ET: embryo transfer, G-CSF: granulocyte colony-stimulating factor,HA: hyaluronic acid. NSAID: non-steroidal anti-inflammatory drug

**Explanations**

a Wide confidence intervals

b Significant heterogeneity in study design: lots of different catheter types compared which we believe may have impacted outcomes

c Downgraded due to concerns about whole risk of bias

d Downgraded due to high heterogeneity: I2 = 68.27%

e Downgraded due to concerns about allocation concealment and lack of blinding

f Downgraded due to high heterogeneity: I2 = 57.76%

g Very wide confidence intervals

h Significant heterogeneity in study design: lots of different NSAID's and length of treatment

i Downgraded due to high heterogeneity: I2 = 75.14%

j Downgraded due to high heterogeneity: I2 = 61.16%

k Downgraded due to high heterogeneity: I2 = 50.68%

l Downgraded due to high heterogeneity: I2 = 60.99%

m Significant heterogeneity in study design, not consistent site of deposition in any of them

o Downgraded due to high heterogeneity: I2 = 75.89%

Three RCTs compared the use of air or fluid in the transfer catheter (Krampl *et al.*, 1995; Moreno *et al.*, 2004; Madani et al., 2010). Both interventions had a similar effect on clinical pregnancy rate (RR 1.361, 95% CI 0.844–2.195) with moderate inconsistency (*I*^2^ = 61.16%; Supplementary Fig. S15). We included three RCTs that compared early versus delayed catheter withdrawal after ET (Martínez *et al.*, 2001; Arvas *et al.*, 2014; Devranoğlu et al., 2018). Both techniques yielded a similar clinical pregnancy rate (n = 3, RR 1.039, 95% CI 0.868–1.243, *I*^2^ = 0%; Supplementary Fig. S16; Martínez *et al.*, 2001; Arvas *et al.*, 2014; Devranoğlu et al., 2018), ongoing pregnancy (n = 1, RR 0.889, 95% CI 0.594–1.329; Devranoğlu et al., 2018) and biochemical pregnancy (n = 1, RR 1.014, 95% CI 0.728–1.413; Devranoğlu et al., 2018).

The site of embryo deposition (>15 mm versus <15 mm from the fundus) was assessed in three RCTs (Nazari *et al.*, 1993; Coroleu *et al.*, 2002a; Franco *et al.*, 2004). Our pooled effect estimate showed no difference between both groups for clinical pregnancy (n = 3, RR 1.089, 95% CI 0.828–1.431, *I*^2^ = 60.99%; Supplementary Fig. S17a; Nazari *et al.*, 1993; Coroleu *et al.*, 2002a; Franco *et al.*, 2004), ongoing pregnancy (n = 3, RR 1.191, 95% CI 0.858–1.653, *I*^2^ = 65.08%; Supplementary Fig. S17b; Nazari *et al.*, 1993; Coroleu *et al.*, 2002a; Franco *et al.*, 2004), miscarriage (n = 2, RR 0.916, 95% CI 0.446–1.881; Supplementary Fig. S17c; Coroleu *et al.*, 2002a; Franco *et al.*, 2004) or live birth (n = 3, RR 1.201, 95% CI 0.855–1.687, *I*^2^ = 66.36%; Supplementary Fig. S17d; Nazari *et al.*, 1993; Coroleu *et al.*, 2002a; Franco *et al.*, 2004). One small RCT (Groutz *et al.*, 1997; n = 40 participants) compared the outcome of transmyometrial to transcervical ultrasound-guided ET showing no difference in the rate of clinical pregnancy (RR 0.333, 95% CI 0.038–2.939) or ongoing pregnancy (RR 0.143, 95% CI 0.008–2.599).

#### Bed rest

We performed a meta-analysis of six RCTs that evaluated the effect of bed rest following ET compared to immediate ambulation or no rest (Botta and Grudzinskas, 1997; Rezábek *et al.*, 2001; Amarin and Obeidat, 2004; Purcell *et al.*, 2007; Gaikwad *et al.*, 2013; Malhotra and Sarkar, 2019). Overall, there was a reduction in clinical pregnancy rates in the bed rest group (RR 0.857, 95% CI 0.741–0.991, *I*^2^ = 0.01%; Fig. 4a, Supplementary Fig. S18a) while there was no significant difference for biochemical pregnancy (n = 2, RR 0.823, 95% CI 0.538–1.259; Supplementary Fig. S18b; Amarin and Obeidat, 2004; Gaikwad *et al.*, 2013), ongoing pregnancy (n = 4, RR 0.870, 95% CI 0.656–1.153, *I*^2^ = 50.53%; Supplementary Fig. S18c; Botta and Grudzinskas, 1997; Amarin and Obeidat, 2004; Purcell *et al.*, 2007; Gaikwad *et al.*, 2013), miscarriage (n = 3, RR 1.076, 95% CI 0.466–2.483, *I*^2^ = 61.84%; Supplementary Fig. S18d; Botta and Grudzinskas, 1997; Amarin and Obeidat, 2004; Gaikwad *et al.*, 2013) or live birth (n = 3, RR 0.800, 95% CI 0.626–1.022, *I*^2^ = 21.93%; Fig. 4b;Supplementary Fig. S18e; Rezábek *et al.*, 2001; Gaikwad *et al.*, 2013; Malhotra and Sarkar, 2019).

#### Nurse versus doctor

Two RCTs compared ET outcomes when performed by a nurse or a doctor (n = 655 participants) showing no significant difference between both groups for clinical pregnancy (n = 2, RR of 0.961, 95% CI 0.778–1.187; Supplementary Fig. S19; Bjuresten et al., 2003; Rinaldi *et al.*, 2014) or ongoing pregnancy (n = 1, RR 0.981, 95% CI 0.728–1.324; Rinaldi *et al.*, 2014).

### Relaxation interventions

#### Acupuncture

Eighteen RCTs evaluated the effect of acupuncture interventions on clinical pregnancy (n = 6437 participants) including 16 studies on needle acupuncture (Paulus *et al.*, 2002, 2003; Benson *et al.*, 2006; Westergaard *et al.*, 2006; Craig *et al.*, 2007, 2014; Fratterelli *et al.*, 2008; Domar et al., 2009; So *et al.*, 2009, 2010; Andersen *et al.*, 2010; Madaschi *et al.*, 2010; Omodei *et al.*, 2010; Moy *et al.*, 2011; Qu *et al.*, 2014; Dehghani *et al.*, 2020), two on laser acupuncture (Benson *et al.*, 2006; Fratterelli *et al.*, 2008) and two on transcutaneous electrical acupoint stimulation (TEAS; Zhang *et al.*, 2011; Zhong and Zhang, 2017). Eight studies compared acupuncture to sham acupuncture therapy (Paulus *et al.*, 2003; Benson *et al.*, 2006; Fratterelli *et al.*, 2008; So *et al.*, 2009, 2010; Andersen *et al.*, 2010; Moy *et al.*, 2011; Zhang *et al.*, 2011), whilst 12 of the studies compared acupuncture to no treatment (Paulus *et al.*, 2002; Benson *et al.*, 2006; Westergaard *et al.*, 2006; Craig *et al.*, 2007, 2014; Fratterelli *et al.*, 2008; Domar et al., 2009; Madaschi *et al.*, 2010; Omodei *et al.*, 2010; Qu *et al.*, 2014; Zhong and Zhang, 2017; Dehghani *et al.*, 2020). Overall, acupuncture interventions did not increase the rate of clinical pregnancy (RR 1.121, 95% CI 0.988–1.273, *I*^2^ = 75.14%; Supplementary Fig. S20a). Subgroup analysis showed a similar effect for needle (n = 16, RR 1.076, 95% CI 0.919–1.260, *I*^2^ = 73.44%) and laser acupuncture (n = 2, RR 1.193, 95% CI 0.996–1.430, *I*^2^ = 0%) whilst TEAS showed some improvement in clinical pregnancy rate (n = 2, RR 1.345, 95% CI 1.039–1.741, *I*^2^ = 62.66%) but confidence in this evidence was low (Supplementary Fig. S20f).

Compared to standard care or sham intervention, acupuncture showed some benefit in increasing the rate of biochemical pregnancy (n = 8, RR 1.150, 95% CI 1.002–1.321, *I*^2^ = 70.45%; Benson *et al.*, 2006; Westergaard *et al.*, 2006; Craig *et al.*, 2007; Fratterelli *et al.*, 2008; Domar et al., 2009; Zhang *et al.*, 2011; Zhong and Zhang, 2017; Dehghani *et al.*, 2020) though evidence was of poor quality with high heterogeneity. There was no difference for ongoing pregnancy (n = 11, RR 1.130, 95% CI 0.931–1.371, *I*^2^ = 75.16%; Paulus *et al.*, 2002, 2003; Westergaard *et al.*, 2006; Fratterelli *et al.*, 2008; So *et al.*, 2009, 2010; Andersen *et al.*, 2010; Madaschi *et al.*, 2010; Omodei *et al.*, 2010; Seto *et al.*, 2017; Dehghani *et al.*, 2020), miscarriage (n = 10, RR 1.073, 95% CI 0.828–1.391, *I*^2^ = 0.0%; Paulus *et al.*, 2002, 2003; Westergaard *et al.*, 2006; So *et al.*, 2009, 2010; Andersen *et al.*, 2010; Madaschi *et al.*, 2010; Omodei *et al.*, 2010; Zhang *et al.*, 2011; Craig *et al.*, 2014) or live birth (n = 11, RR 1.176, 95% CI 0.929–1.488, *I*^2^ = 80.92%; Paulus *et al.*, 2002, 2003; So *et al.*, 2009, 2010; Andersen *et al.*, 2010; Madaschi *et al.*, 2010; Omodei *et al.*, 2010; Zhang *et al.*, 2011; Craig *et al.*, 2014; Qu *et al.*, 2014; Seto *et al.*, 2017; Fig. 4b, Supplementary Fig. S20b–e). There were no significant differences in clinical pregnancy rates observed between studies using a sham acupuncture control (n = 8, RR 1.056, 95% CI 0.890–1.252); or those with no placebo comparison (n = 12, RR 1.177, 95% CI 0.988–1.273; Supplementary Fig. S20g).

Listening to music during ET was assessed in three RCTs compared to standard care (Murphy *et al.*, 2014; Stocker *et al.*, 2016; Aba *et al.*, 2017). Overall, this did not increase the rate of clinical pregnancy (RR 1.052, 95% CI 0.866–1.279, *I*^2^ = 0.00%; Supplementary Fig. S21). Similarly, the use of mindfulness was assessed in two RCTs (Benson *et al.*, 2006; Fratterelli *et al.*, 2008) also showing no difference in clinical pregnancy (RR 0.911, 95% CI 0.755–1.098, *I*^2^ = 0.00%) or biochemical pregnancy rate (RR 0.912, 95% CI 0.780–1.066, *I*^2^ = 0%; Supplementary Fig. S22a and b, respectively). The effect of mindfulness on ongoing pregnancy rate was reported in one study (Fratterelli *et al.*, 2008) showing no significant increase was seen (RR 0.946, 95% CI 0.738–1.211).

One study measured the effect of porcine relaxin compared to a placebo (MacLennan *et al.*, 1985). There was no increase in clinical pregnancy (RR 0.882, 95% CI 0.405–1.924) or ongoing pregnancy rate (RR 1.177, 95% CI 0.442–3.134) or miscarriage rate (RR 0.441, 95% CI 0.085–2.296). Similarly, one study measured the effect of glyceryl trinitrate compared to a placebo (Shaker *et al.*, 1993) also showing no increase in clinical pregnancy rate (RR 0.947, 95% CI 0.554–1.620).

There was one study that investigated massage therapy as an intervention compared to standard care (Gavrizi and Skillern, 2019). The results from this study showed no significant increase in live birth with massage (RR 1.371, 95% CI 0.881–2.133). Catoire *et al.* (2013) compared the effect of hypnosis on the day of ET to the use of benzodiazepine in 93 women, which did not report a significant difference for clinical pregnancy (RR 1.032, 95% CI 0.595–1.790) or live birth (RR 1.015, 95% CI 0.509–2.03).

## Discussion

### Summary of main findings

In this comprehensive systematic review, we identified a large number of competing interventions proposed to increase the chances of conception following ET. Overall, the quality of evidence was low with significant concerns regarding trial methodology and risk of publication bias. Several interventions do seem, however, to significantly improve the chances of conception (Table I).

The use of ultrasound guidance and soft ET catheters are commonly used in clinical practice with good quality evidence to support their effectiveness. We identified several promising pharmacological agents that also seem to aid embryo implantation and increase clinical pregnancy including HA, G-CSF, atosiban and hCG. A common mechanistic effect across these interventions could be through maximizing uterine relaxation at the time of ET and optimizing endometrial receptivity (Mains and van Voorhis, 2010). Evidence for these interventions largely stems from small trials and further evaluation in larger trials is warranted before adopting them into routine clinical practice.

Recommending bed rest post-ET was the only intervention in this review that significantly reduced clinical pregnancy rates. This is consistent with ASRM Guidance, which recommends against this intervention (Penzias *et al.*, 2017a). We identified a substantial number of unnecessary interventions that lack sufficient evidence in support of their effectiveness and safety. Such interventions should not be routinely recommended in clinical practice pending assessment in future randomized trials.

### Strengths and limitations

The strengths of our review stem from its pragmatic design and comprehensive search strategy, therefore, offering the most up to date evidence on ET techniques and interventions. We employed a standard methodology and assessed the risk of bias in included trials following a prospectively registered protocol. We conducted subgroup analyses to explore potential effect modifiers, assessed publication bias and employed the GRADE approach to aid evidence translation into clinical practice.

Our findings have several methodological limitations. The quality of included trials was often suboptimal with almost half showing a high risk of bias for outcome reporting (44.9%). This limited our ability to synthesize evidence on all outcomes of interest, particularly live birth. We addressed this perceived bias by downgrading confidence in available evidence in our GRADE assessment of the evaluated interventions; however, this remains a subjective judgement of evidence quality and certainty.

Several effect modifiers could impact the outcome of ET including the operator’s experience, patient characteristics (age, BMI, uterine anomalies, etc.), number of embryos transferred and the transfer of fresh versus frozen embryos. Clearly, the technology and practice of assisted conception have also progressed significantly over the time span of included trials (1989–2021) leading to inherent variations in evaluated ART protocols (Supplementary Table SI). We were unable to explore certain effect modifiers due to reporting limitations, however, we attempted to address this by implementing a random-effect model to adjust our pooled effect estimate (Cornell *et al.*, 2014). Further evaluation using individual patient data (IPD) is, therefore, warranted to synthesize higher-quality evidence.

### Implications for clinical practice

Currently, a large number of add-on treatments are proposed to couples undergoing ART, yet the debate on their effectiveness and safety prevail (Zemyarska, 2019; Lensen *et al.*, 2021). We detected many RCTs conducted within the last 20 years evaluating many different interventions focused on optimizing ET success. This highlights the important role of robust evidence synthesis to inform clinical practice and minimize the risk of exposing patients to potentially harmful or unnecessary interventions.

Most of the published systematic reviews on ET techniques are clustered around interventions commonly used in current practice such as ultrasound guidance (Teixeira *et al.*, 2015; Cozzolino *et al.*, 2018) and acupuncture (El-Toukhy *et al.*, 2008; Gu *et al.*, 2019; Smith *et al.*, 2019). While helpful, systematic reviews focused on evaluating singular interventions can lead to fragmented evidence synthesis and an underappreciation of the body of evidence relevant to a particular health condition. To that end, we implemented a comprehensive search strategy, which helped us to detect several interventions of uncertain benefit that were evaluated in one or two RCTs only. We, therefore, caution against the routine uptake of such ‘experimental’ interventions into clinical practice pending further evaluation in larger, well-conducted RCTs. Although we detected several ET interventions that appear to improve chances of conception, the current body of evidence remains imprecise, and these interventions should be offered in research settings only.

Publication bias and selective outcomes reporting are major barriers to robust evidence synthesis (Sutton *et al.*, 2000) specifically in the context of high stakes interventions such as ET. We chose to report primarily on clinical pregnancy rate to offer the most comprehensive evidence synthesis as most of the included trials did not report on key additional outcomes such as live birth. Bed rest is an important example where varied outcomes reported in primary RCTs (Botta and Grudzinskas, 1997; Rezábek *et al.*, 2001; Amarin and Obeidat, 2004; Purcell *et al.*, 2007; Gaikwad *et al.*, 2013; Malhotra and Sarkar, 2019) could have contributed to the delay in identifying its adverse impact on clinical pregnancy rate following ET. As such, robust evaluation of the confidence in available evidence is crucial before introducing changes to clinical practice (Pottie *et al.*, 2012).

Professional societies should champion the concurrent evaluation of available evidence to regularly update clinical practice guidelines. For example, the most up to date ASRM guidelines on ET recommend cervical mucus removal based on ‘fair’ evidence at the time (one RCT and one cohort study; Penzias *et al.*, 2017a). Still, our findings summarizing evidence from five RCTs showed no benefit of this intervention.

Finally, the process of ET involves several steps immediately before, during and after where the operator technique seems to significantly impact the final outcome (Cirillo *et al.*, 2020). Establishing a standardized protocol of best practice may facilitate training and reduce variation in practice across fertility clinics (Penzias *et al.*, 2017b).

### Future research need

The majority of the included RCTs in our review had a relatively small sample size with a moderate risk of bias specifically for outcome reporting. This significantly hinders efficient evidence synthesis and increases the risk of small study effect (Harbord *et al.*, 2009). While several of the evaluated interventions seem to have a promising effect in improving clinical pregnancy rate (e.g. Atosiban and hCG), there is a need to evaluate their effectiveness and safety in well-powered RCTs and prospective IPD meta-analyses to inform clinical practice at scale (Tierney *et al.*, 2015).

In this review, we focused on interventions within 24 h of the ET to increase homogeneity across pooled RCTs. However, several interventions aimed to improve endometrial receptivity and the implantation window could improve the outcome ET outcome such as endometrial scratching and different methods of luteal support (Mains and van Voorhis, 2010). Further evidence synthesis is, therefore, required to advise on the best practice in preparation for ET.

Finally, the majority of the included RCTs did not engage lay consumers in their design, conduct or reporting. Patient engagement is particularly relevant when evaluating anti-anxiety and relaxation interventions during ART treatments, which to date seem to have been based on anecdotal practice (Mahlstedt *et al.*, 1987). Future trials should aim to engage all relevant stakeholders in the ART process to improve the permeation of research into clinical practice.

## Conclusion

Using ultrasound guidance, soft catheters and HA at the time of ET appears to increase pregnancy rates. The use of atosiban, G-CSF and hCG showed a trend towards increased clinical pregnancy rate, but larger trials are required before adopting these interventions in clinical practice. Bed rest post-ET was associated with a reduction in clinical pregnancy and should not be recommended.

## Supplementary data

Supplementary data are available at *Human Reproduction Update* online.

## Data availability

Some or all datasets generated during and/or analysed during the current study are not publicly available but are available from the corresponding author on reasonable request.

## Authors’ roles

B.T., H.W. and J.T. conducted the data extraction, primary analysis, data illustration and drafted the first manuscript. S.D.K., D.M. and E.Y. supervised the project conduct and provided critical input to the final manuscript. B.H.A. conceived the idea, wrote the protocol, supervised the analysis and edited the final manuscript.

## Funding

No funding was received towards this work directly. B.H.A. holds a personal development award from the National Institute of Health Research.

## Conflict of interest

Nothing to disclose.

## Supplementary Material

dmac009_Supplementary_Data
