# Supplementary material for: Interventions to optimize embryo transfer in women undergoing assisted conception: a comprehensive systematic review and meta-analyses
Source: Hum Reprod Update. 2022 Mar 24;28(4):480–500. doi: 10.1093/humupd/dmac009 (PMC9631462; doi:10.1093/humupd/dmac009)
Supplement: dmac009_Supplementary_Data [file dmac009_supplementary_data.docx]

**Interventions to optimise embryo transfer in women undergoing assisted conception: A comprehensive systematic review and meta-analyses.**

Bede Tyler^1^, Hugo Walford^1^, Jennifer Tamblyn^2^, Stephen Keay^3^, Dimitrios Mavrelos^1,4^, Ephia Yasmin^1,4^, Bassel H. Al Wattar^1,4^.

**Supplementary materials**

- **Supplementary Table I:** Characteristics of included randomised trials evaluating interventions to optimise the reproductive outcomes at the time of embryo transfer.
- **Supplementary Table II:** List of excluded studies.
- **Supplementary Table III:** Summary of risk of bias across included randomised trials evaluating interventions to optimise the reproductive outcomes at the time of embryo transfer.
- **Supplementary Table IV:** GRADE assessment of evidence evaluating all interventions performed at the time of embryo transfer in women undergoing assisted reproduction
- **Supplementary Figure S1:** Funnel plot to evaluate the risk of publication bias in included randomised trials evaluating interventions to optimise the reproductive outcomes at the time of embryo transfer after excluding interventions evaluated in a single RCT.
- **Supplementary Figure S2:** Forest plot of the effectiveness of using anti-biotics at the time of embryo transfer on reproductive outcomes.

a: clinical pregnancy

b: biochemical pregnancy

c: ongoing pregnancy

d: miscarriage

e: subgroup analysis by anti-biotics dose on clinical pregnancy

- **Supplementary Figure S3:** Forest plot of the effectiveness of using Atosiban at the time of embryo transfer on reproductive outcomes.

a: clinical pregnancy

b: biochemical pregnancy

c: ongoing pregnancy

d: miscarriage

e: subgroup analysis by Atosiban dose on clinical pregnancy

- **Supplementary Figure S4:** Forest plot of the effectiveness of using NSAIDs at the time of embryo transfer on reproductive outcomes.

a: clinical pregnancy

b: biochemical pregnancy

c: miscarriage

d: subgroup analysis by type of NSAID on clinical pregnancy

- **Supplementary Figure S5:** Forest plot of the effectiveness of using hCG at the time of embryo transfer on reproductive outcomes.

a: clinical pregnancy

b: biochemical pregnancy

c: ongoing pregnancy

d: miscarriage

e: live birth

f: subgroup analysis by hCG dose on clinical pregnancy

g: subgroup analysis by comparison (control injection or no treatment) on clinical pregnancy

- **Supplementary Figure S6:** Forest plot of the effectiveness of using hyaluronic acid at the time of embryo transfer on reproductive outcomes.

a: clinical pregnancy

b: ongoing pregnancy

c: live birth

- **Supplementary Figure S7:** Forest plot of the effectiveness of high vs low doses of hyaluronic acid at the time of embryo transfer on reproductive outcomes.

a: clinical pregnancy

b: ongoing pregnancy

c: live birth

- **Supplementary Figure S8:** Forest plot of the effectiveness of using G-CSF at the time of embryo transfer on clinical pregnancy
- **Supplementary Figure S9:** Forest plot of the effectiveness of using seminal fluid at the time of embryo transfer on biochemical pregnancy
- **Supplementary Figure S10:** Forest plot of the effectiveness of ultrasound vs clinical touch on reproductive outcomes at the time of embryo transfer.

a: clinical pregnancy

b: biochemical pregnancy

c: ongoing pregnancy

d: miscarriage

e: live birth

f: subgroup analysis by ultrasound route (TA vs TV) on clinical pregnancy

g: subgroup analysis by ultrasound route (TA vs TV) on biochemical pregnancy

h: subgroup analysis by ultrasound route (TA vs TV) on ongoing pregnancy

i: subgroup analysis by ultrasound route (TA vs TV) on miscarriage

- **Supplementary Figure S11:** Forest plot of the effectiveness of bladder fullness vs no intervention at the time of embryo transfer on reproductive outcomes.

a: clinical pregnancy

b: ongoing pregnancy

- **Supplementary Figure S12:** Forest plot of the effectiveness of pressure on cervix at the time of embryo transfer on clinical pregnancy
- **Supplementary Figure S13:** Forest plot of the effectiveness of cervical mucus removal at the time of embryo transfer on reproductive outcomes.

a: clinical pregnancy

b: biochemical pregnancy

c: ongoing pregnancy

d: miscarriage

e: live birth

- **Supplementary Figure S14:** Forest plot of the effectiveness of using soft vs hard embryo transfer catheters at the time of embryo transfer on reproductive outcomes.

a: clinical pregnancy

b: ongoing pregnancy

c: live birth

- **Supplementary Figure S15:** Forest plot of the effectiveness of using air vs fluid in the embryo transfer catheter on clinical pregnancy
- **Supplementary Figure S16:** Forest plot of the effectiveness of early vs delayed catheter withdrawal at the time of embryo transfer on clinical pregnancy.
- **Supplementary Figure S17:** Forest plot of the effect of the site of embryo deposition at the time of embryo transfer on reproductive outcomes.

a: clinical pregnancy

b: ongoing pregnancy

c: miscarriage

d: live birth

- **Supplementary Figure S18:** Forest plot of the effectiveness of bed rest following embryo transfer on reproductive outcomes.

a: clinical pregnancy

b: biochemical pregnancy

c: ongoing pregnancy

d: miscarriage

e: live birth

- **Supplementary Figure S19:** Forest plot comparing the difference in clinical pregnancy following embryo transfer performed by nurse vs doctor.
- **Supplementary Figure S20:** Forest plot of the effectiveness of using acupuncture at the time of embryo transfer on reproductive outcomes.

a: clinical pregnancy

b: biochemical pregnancy

c: ongoing pregnancy

d: miscarriage

e: live birth

f: subgroup analysis by type of acupuncture on clinical pregnancy

g: subgroup analysis by comparison (sham acupuncture or no treatment) on clinical pregnancy

- **Supplementary Figure S21:** Forest plot of the effectiveness of music at the time of embryo transfer on clinical pregnancy
- **Supplementary Figure S22:** Forest plot of the effectiveness of mindfulness at the time of embryo transfer on reproductive outcomes.

a: clinical pregnancy

b: biochemical pregnancy

- **Supplementary Appendix S1:** Literature search strategy.

**Supplementary Table SI:** Characteristics of included randomised trials evaluating interventions to optimise the reproductive outcomes at the time of embryo transfer

| CHARACTERISTICS OF INCLUDED STUDIES EVALUATING THE EFFECTS OF ACUPUNCTURE ON CLINICAL PREGNANCY RATES | | | | | | | |  |
| --- | --- | --- | --- | --- | --- | --- | --- | --- |
| Study | **Country** | **Number** | **Inclusion/Exclusion Criteria** | **Intervention** | **Comparison** | **Fresh or Frozen Cycle** | **Stage of Embryo Development** |  |
| Andersen 2010 (1) | Denmark | 635 | I: ⩽37 years old IVF/ICSI  1/2 embryos in 1^st^/2^nd^/3^rd^ cycle.  E: Frozen, other treatment received | Two sessions of needle AC: (1) 30 min pre-ET, (2) 30 min post ET | Non-invasive sham control | FR | D2-3 |  |
| Benson 2006* (2) | USA | 208 | NR | Two sessions of acupuncture: (1) 25 min before ET, (2) 25 min after ET  Two study groups: (1) needle AC, (2) laser AC | Two comparison groups: Sham laser acupuncture (placebo), no adjuvant treatment | NR | D3-5 |  |
| Craig 2007 (3) | USA | 94 | I: Women undergoing IVF who have not had acupuncture within 3 months | Two sessions of needle AC, both at an off-site location: (1) within 1– 2 h before ET; (2) within 1–2 h after ET | No adjuvant treatment | NR | NR |  |
| Craig 2014 (4) | USA | 113 | NR | Two sessions of needle AC: (1) within 1–2 h before ET; (2) within 1–2 h after ET | No adjuvant treatment | FR | D2-5 |  |
| Dehghani 2020* (5) | Iran | 186 | I: Couple's consent, women undergoing ET  E: Did not achieve ET or did not want to participate further | Two study groups: (1) needle AC 25 min before ET, (2) needle AC 25 min before and after ET | No adjuvant treatment | NR | NR |  |
| Domar 2009 (6) | USA | 146 | I: ET on weekend, nondonor eggs | Two sessions of needle AC: (1) 25 min before ET; (2) immediately after ET | Lying quietly for 25 min | FR | NR |  |
| Fratterelli 2008* (7) | USA | 797 | NR | Two sessions: (1) 25 min before ET, (2) 25 min after ET.  Two study groups: (1) needle AC, (2) laser AC | Two comparison groups: sham laser acupuncture, no intervention | NR | NR |  |
| Madaschi 2010 (8) | Brazil | 416 | I: All patients aged ≤35 years, and undergoing ICSI cycles for the first time | Two sessions of needle AC: (1) 25 min before ET; (2) immediately after ET | No adjuvant treatment | NR | D2-3 |  |
| Moy 2011 (9) | USA | 161 | I: Day 3 ET, infertile women ages 18–38, FR IVF or ICSI, acupuncture naïve  E: ≥38 years old, day 5 ET, had prior experience with acupuncture, previously participated in the trial | Two sessions of needle AC: (1) 25 min before ET; (2) immediately after ET | Invasive sham control | FR | D3 |  |
| Ng 2011 (10) | China | 800 | NR | Two sessions of non-invasive acupuncture: (1) for 25 minutes before ET, (2) for 25 mins after ET | No adjuvant treatment | NR | NR |  |
| Omodei 2010 (11) | Italy | 168 | NR | Two sessions of needle AC: (1) 25 min before ET; (1) immediately after ET | No adjuvant treatment | NR | D2-5 |  |
| Paulus 2002 (12) | Germany | 160 | I: Good embryo quality | Two sessions of needle AC: (1) 25 min before ET; (2) immediately after ET | No adjuvant treatment | FR | D2-3 |  |
| Paulus 2003 (13) | Germany | 200 | I: Good embryo quality | Two sessions of needle AC: (1) 25 min before ET; (2) immediately after ET | No adjuvant treatment | FR | NR |  |
| Qu 2014* (14) | China | 305 | I: Infertile women with tubal blockage  E: Not fluent in Chinese, neurologic or psychiatric disorders, taking any tranquillizer, acupressure or acupuncture therapy, an ear deformity, history of smoking or drinking | Two sessions of auricular AC: (1) before ET, (2) after ET | Two comparison groups: (1) Two sessions of sham auricular acupuncture before and after ET, (2) no adjuvant treatment | NR | NR |  |
| Seto 2017 (15) | Hong Kong | 596 | I: <35 years old, undergoing first IVF cycle, endometrial thickness >8mm, 2 good quality embryos (embryo of >/= 4 cells, grade 1 or 2). | Two sessions of 25 mins of needle AC: (1) before ET, (2) after ET | Two sessions of 25 mins of placebo acupuncture instead | FR and FZ | NR |  |
| So 2009 (16) | Hong Kong/ China | 370 | I: IVF, normal uterine cavity on ultrasound  E: Abnormal uterine cavity, cancellation of ET due to failed fertilization/risk of OHSS | Two sessions of needle AC: (1) 25 min before ET; (2) immediately after ET | Non-invasive sham control | FR | D2-3 |  |
| So 2010 (17) | Hong Kong/  China | 226 | I: Subfertile patients, frozen ET, normal uterine cavity shown on ultrasound  E: Frozen embryo(s) replaced in stimulated IVF cycles; lysis of all frozen embryos on thawing; donor oocytes; PGD | One session of needle AC immediately after ET | Non-invasive sham control | FZ | D2-3 |  |
| Westergaard 2006 (18) | Denmark | 182 | I: Couples’ consent | Two sessions of needle AC: (1) 25 min before ET; (2) immediately after ET | No adjuvant treatment | FR | D2-3 |  |
| Zhang 2011* (19) | China | 309 | I: Infertile women 21–44 years old, fresh or frozen, IVF or ICSI  E: Not eligible for ET, adverse ovarian reserve, previous acupuncture experience | Two study groups: (1) single TEAS treatment 30 minutes after ET; (2) double TEAS treatments: 24 hours before ET and 30 minutes after ET | Mock TEAS: 30 minutes after ET | FR/FZ | NR |  |
| Zhong 2017 (20) | China | 1761 | I: IVF-ET patients of natural cycle or hormone replacement cycle | Two sessions of TEAS: (1) 30 min 24 h before ET, (2) 30 min within 2 h after transfer | No adjuvant treatment | NR | NR |  |
| CHARACTERISTICS OF INCLUDED STUDIES EVALUATING THE EFFECTS OF AIR VS FLUID IN THE TRANSFER CATHETER ON CLINICAL PREGNANCY RATES | | | | | | | |  |
| Study | **Country** | **Number** | **Inclusion/Exclusion Criteria** | **Intervention** | **Comparison** | **Fresh or Frozen Cycle** | **Stage of Embryo Development** |  |
| Krampl 1995 (21) | Austria | 196 | NR | Aspirated without being sealed by air bubbles | Loading was performed using traditional methods | FR | D2-3 |  |
| Madani 2010 (22) | Iran | 110 | E: Aged >40 years, frozen-thawed ET, oocyte-donation cycles | Pushing 0.2 mL of air into the catheter immediately after ET | Standard ET | FR | D2-3 |  |
| Moreno 2004 (23) | Spain | 102 | I: Regularly menstruating premenopausal women, age 29–42 years, IVF/ICSI indications included male factor, tubal infertility, unexplained infertility, endometriosis, both ovaries. Uterine cavity was normal in all patients.  E: Occult ovarian failure on the basis of their cycle day 3 FSH concentration, which was 12 IU/L measured within the three cycles before IVF/ICSI. | Embryos were loaded as follows: 200 L of air in the syringe, 100–125 L of air in the proximal part of the catheter, 20–25 L of medium containing the embryos to be transferred, and 10 L of air at the tip of the catheter. | The syringe and the entire catheter were filled with medium and the embryo-containing medium (20–25 L) was aspirated without being bracketed by air spaces | FR | NR |  |
| CHARACTERISTICS OF INCLUDED STUDIES EVALUATING THE EFFECTS OF ANTIBIOTICS BEFORE EMBRYO TRANSFER ON CLINICAL PREGNANCY RATES | | | | | | | |  |
| Study | **Country** | **Number** | **Inclusion/Exclusion Criteria** | **Intervention** | **Comparison** | **Fresh or Frozen Cycle** | **Stage of Embryo Development** |  |
| Brook 2006 (24) | UK | 350 | E: Penicillin allergy, undergoing oncology freeze, on concurrent antibiotics, requiring antibiotic prophylaxis at the time of transvaginal oocyte collection | Patients were randomly allocated to antibiotics (1.5g of co-amoxyclav tablets - 750mg the night before the transfer and 750mg 2 hours prior to transfer) | No-treatment. No placebo tablets were used. | Fresh | NR |  |
| Peikrishvili 2004 (25) | France | 275 | I: Age <38 years | Amoxicillin & clavulanic acid from the day of ET | Control group | NR | NR |  |
| CHARACTERISTICS OF INCLUDED STUDIES EVALUATING THE EFFECTS OF ATOSIBAN BEFORE EMBRYO TRANSFER ON CLINICAL PREGNANCY RATES | | | | | | | |  |
| Study | **Country** | **Number** | **Inclusion/Exclusion Criteria** | **Intervention** | **Comparison** | **Fresh or Frozen Cycle** | **Stage of Embryo Development** |  |
| Ahn 2009 (26) | Korea | 40 | I: >2 failures of IVF/ICSI  E: Low ovarian reserve | Intravenous administration of atosiban started with a bolus dose 6.75 mg one hour before ET and continued at an infusion rate of 18 mg/hour. After ET, administered atosiban was reduced to 6 mg/hour and continued for 2 hours. | Control group received nothing. | FR | D3 |  |
| Bosch 2019 (27) | Multinational | 255 | I: age 18-37 years, history of repeated implantation failure  E: Uterine pathology and thrombophilia disease | Patients received either barusiban (40 mg 45min pre-transfer + 10 mg 15min post-transfer) administered subcutaneously | Placebo, administered subcutaneously | NR | D3-5 |  |
| He 2016 (28) | China | 120 | I: Endometriosis, frozen–thawed ET, aged 20–45 years; baseline FSH <10 IU/L; normal serum CA-125 level (<35 IU/L); one or more day-5 good-quality embryo(s) available for transfer; <3 previous ET cycle failures  E: Uterine anomaly; uterine fibroids; hydrosalpinges; fresh embryo transfer; received GnRH agonists or antagonists (GnRH analogous) before FET; endometrial thickness <8 mm; day-3 ET;] endocrine disorders | Single bolus (6.75 mg, 0.9 mL per vial) of atosiban was administrated before ET | No atosiban | FZ | D5 |  |
| Hebisha 2016 (29) | Egypt | 182 | I: ICSI, male or tubal factor infertility, using long agonist protocol | Group A received 7.5 mg Atosiban by slow IV injection 20 minutes before ET | Group B received placebo as sodium chloride 0.9% solution also by IV injection 20 minutes before ET | NR | D5 |  |
| Moraloglu 2010 (30) | Turkey | 180 | I: Basal FSH hormone concentration <10 IU/l; age 20– 39 years; first IVF cycle; long protocol with GnRH agonist and rFSH; at least two top-quality embryos after ICSI.  E: Age >39 years; severe male factor (10 IU/ml); clinically relevant systemic disease (e.g. diabetes mellitus type 1); >3 previous failed IVF cycles, IVF cycle cancelled due to poor response to gonadotrophins; uterine anomaly, uterine fibroids, hydrosalpinges; frozen–thawed cycles; only one top-quality embryo after ICSI; difficult transfer | The treatment group received intravenous administration of atosiban before embryo transfer with a total administered dose of 37.5 mg. | In the control group, the same number of cycles was performed with placebo medication. | FR | D3 |  |
| Ng 2014 (31) | China, Hong Kong, Vietnam | 800 | I: IVF, age <43 years, normal uterine cavity shown on ultrasound scanning  E: ≥3 previous IVF cycles; donor oocytes;) natural IVF or in vitro maturation cycles; endometrial thickness <8 mm; hydrosalpinx;) transfer cancelled due to no fertilization or risk of OHSS; blastocyst transfer; PGD; recruited in the same study before; joined other studies in the centres. | Women in the atosiban group received i.v. atosiban 30 min before embryo transfer with a bolus dose of 6.75 mg, and the infusion was continued at 18 mg/h for 1 h. The dose of atosiban was then reduced to 6 mg/h continued for another 2 h. | Those in the placebo group received i.v. normal saline only | NR | D2-3 |  |
| Song 2013 (32) | China | 120 | NR | 37.5mg: Atosiban - intravenous administration of atosiban before 30 minutes of embryo transfer with a total administered dose of 37.5 mg | No adjuvant treatment | NR | D2-3 |  |
| Tournaye 2017 (33) | Czech Republic, Belgium, Switzerland, UK | 778 | I: Age <36 years, <1 failed ART cycle, GnRH antagonist, <1.5 ng/mL serum progesterone on hCG day, luteal support with vaginal micronized progesterone | Single oral 900 mg dose of nolasiban administered about 4 hours before ET following IVF/ICSI. | Placebo dose administered about 4 hours before ET following IVF/ICSI. | NR | D3-5 |  |
| Yuan 2019 (34) | China | 204 | I: Previous difficult IVF transfer, age <43 years, normal uterine cavity, clear information about previous IVF-ET cycles (including number of embryos transferred, embryo quality, endometrial thickness), ≥1 more good-quality embryos after retrieving on the day of ET.  E: Uterine anomaly, hydrosalpinx, endometrial thickness <7.5 mm, blastocyst transfer. | In atosiban group, atosiban with a total dose of 37.5 mg was administered. | Placebo - normal saline infusion for the same duration | FZ | D2-3 |  |
| CHARACTERISTICS OF INCLUDED STUDIES EVALUATING THE EFFECTS OF BED REST AFTER EMBRYO TRANSFER ON CLINICAL PREGNANCY RATES | | | | | | | |  |
| Study | **Country** | **Number** | **Inclusion/Exclusion Criteria** | **Intervention** | **Comparison** | **Fresh or Frozen Cycle** | **Stage of Embryo Development** |  |
| Amarin 2004 (35) | Jordan | 378 | I: Age <40 years, undergoing IVF with GnRH pituitary down-regulation and controlled ovarian hyperstimulation.  E: Age >40 years | Rest for 24 hours after ET | Rest for 1 hour after ET | FR | NR |  |
| Botta and Grudzinskas 1997 (36) | Italy | 180 | I: Infertile patients undergoing IVF embryo transfer at the clinic | Rest for 24 hours after ET | Rest for 20 minutes after ET | FR | D3 |  |
| Gaikwad 2013 (37) | Spain | 240 | I: Infertile patients undergoing IVF embryo transfer, age 25-49 years, first cycle of IVF, oocyte donation  E: Uterine fibroid/s, recurrent miscarriages with >2 abortions, unilateral or bilateral hydrosalpinx, BMI >28 kg/m^2^, recipients opted for single ET, partner diagnosed severe oligoasthenoteratozoospermia, patient chose not to participate in the study | Ten minutes of bed rest after ET | No bed rest | FR | D3-5 |  |
| Malhotra 2019 (38) | India | 180 | I: Fresh IVF/ICSI cycles with age 25-38 years, BMI 18-28 kg/m^2^, normal endometrial cavity  E: Frozen ET, uterine fibroid, adenomyosis of uterus, unilateral or bilateral hydrosalpinx, poor endometrium < 6mm at time of ET | Rest for 15 minutes after ET | No bed rest | FR | NR |  |
| Purcell 2007 (39) | USA | 164 | I: Infertile patients undergoing IVF | Rest for 30 minutes after ET | No bed rest | NR | D2-3 |  |
| Rezábek 2001 (40) | Czech Republic | 38 | I: Infertile patients undergoing IVF | Rest overnight after ET | Rest for 20 minutes after ET | NR | NR |  |
| CHARACTERISTICS OF INCLUDED STUDIES EVALUATING THE EFFECTS OF CATHETER WITHDRAWAL ON CLINICAL PREGNANCY RATES | | | | | | | |  |
| Study | **Country** | **Number** | **Inclusion/Exclusion Criteria** | **Intervention** | **Comparison** | **Fresh or Frozen Cycle** | **Stage of Embryo Development** |  |
| Arvas 2014 (41) | Turkey | 277 | NR | Embryo transfer catheter was gently withdrawn immediately | Embryo transfer catheter was gently withdrawn after a 20-second wait | FR | D5 |  |
| Devranoglu 2016 (42) | Turkey | 295 | I: ICSI, age <40 years, ≥1 fresh embryo of good quality  E: Frozen embryos, donor ETs, suspected uterine cavity abnormalities such as endometrial polyps or submucous fibroids, cervical pathologies, history of intrauterine adhesions | ET catheter was removed slowly within the first 5 s after the ET | ET catheter was removed following a 30-s wait | FR | D2-5 |  |
| Martinez 2001 (43) | Spain | 100 | I: IVF patients, ≥2 embryos of good quality, no difficulties during transfer | Slow withdrawal of the catheter immediately after embryo injection | Withdrawal of the catheter after a 30 s wait. | FR | D2-6 |  |
| CHARACTERISTICS OF INCLUDED STUDIES EVALUATING THE EFFECTS OF CERVICAL MUCUS REMOVAL BEFORE EMBRYO TRANSFER ON CLINICAL PREGNANCY RATES | | | | | | | |  |
| Study | **Country** | **Number** | **Inclusion/Exclusion Criteria** | **Intervention** | **Comparison** | **Fresh or Frozen Cycle** | **Stage of Embryo Development** |  |
| Berkkanoglu 2006 (44) | Turkey | 181 | I: Age <40 years  E: Difficult transfer, uterine abnormalities unintentional reflux of media at time of cervical irrigation | Cervical irrigation with intrauterine flushing before ET | Cervical irrigation without intrauterine flushing before ET | NR | D2 |  |
| Glass 2000 (45) | USA | 253 | I: Age <40 years, day 3 ET  E: Frozen embryo transfer, previous cervical cone biopsy, donor egg ET | Mucus removal by aspiration and cervical irrigation, flushing endocervical canal | No mucus removal | FR | D3 |  |
| Moini 2011 (46) | Iran | 530 | I: Age <35 years, >2 good-quality embryos, fresh ET  E: Uterine factor infertility, congenital abnormalities such as bicornuate, unicornuate, septate or didelphis uteri, acquired abnormalities including uterine ﬁbroids, polyps, hyperplasia, intrauterine adhesions, endometritis, or hyperplasia, | The cervical canal was cleansed using sterile cotton swabs prior to ET. | Routine ET without additional cervical canal manipulation | FR | D2-3 |  |
| Ruhlman 1999 (47) | Argentina | 97 | NR | Mucus removal by aspiration | No mucus removal | NR | NR |  |
| Soroka 1999 (48) | Canada | 96 | NR | Mucus removal by cervical brush | No mucus removal | NR | NR |  |
| Visschers 2007 (49) | Netherlands | 428 | NR | Cervical mucus was removed using a cervical brush, as commonly used to collect cervical PAP smear tests. | Mock procedure - ectocervix was touched with a cotton wool swab, but no endocervical mucus occurred. | FR/FZ | D2-3 |  |
| CHARACTERISTICS OF INCLUDED STUDIES EVALUATING THE EFFECTS OF EMBRYO TRANSFER CATHETER TYPE ON CLINICAL PREGNANCY RATES | | | | | | | |  |
| Study | **Country** | **Number** | **Inclusion/Exclusion Criteria** | **Intervention** | **Comparison** | **Fresh or Frozen Cycle** | **Stage of Embryo Development** |  |
| Al Shawaf 1993 (50) | UK | 224 | NR | Soft catheter - Wallace catheter | Firm catheter - Frydman | FR/FZ | NR |  |
| Allahbadia 2010 (51) | Israel | 175 | I: Donor egg IVF-ET  E: Already in a trial, woman receiving eggs from multiple donors | Softer catheter - Sureview | Firmer catheter - Wallace | NR | NR |  |
| Almodin 2009 (52) | Brazil | 93 | I: Aged 25-39 | New flexible catheter - Ingamed | Firmer catheter - Sydney Catheter | NR | D3 |  |
| Amorocho 1999 (53) | Spain | 214 | NR | Soft catheter - Gynetics Delphin | Firm catheter - Gynetics Emtrac-A | FR | NR |  |
| Ata 2007 (54) | Turkey | 260 | I: First assisted reproduction trial | Soft catheter - Wallace | Intermediate catheter - Labotect | FR | D3 |  |
| Boone 2001 (55) | USA | 113 | NR | Softer catheter - Cook | Firm catheter - Wallace catheter | NR | D3 or D6 |  |
| Candan 2014 (56) | Belgium | 227 | I: <35 years old, fresh single blastocyst transfer | Soft catheter - Cook | Firm catheter - Prodimed | FR | D5 |  |
| Coroleu 2006 (57) | Spain | 186 | I: Age 25-43 years, included male factor, tubal infertility, unexplained infertility, and endometriosis | Echogenic soft Wallace catheter | Standard soft Wallace catheter | FR | D2-3 |  |
| Curfs 2001 (58) | Netherlands | 480 | NR | Soft catheter - Edwards-Wallace | Firm catheter - Tight difficult transfer | FR | NR |  |
| El-Sharwarby 2008 (59) | UK | 308 | I: IVF at the unit  E: Single embryo transfers, PGD, gamete donation, or chemo/radiotherapy patients, repeated IVF failures (>6), previously difficult embryo transfer. | Soft catheter - Wallace | Firm catheter - RocketEmbryon | FR | D2-3 |  |
| Foutouh 2003 (60) | Egypt | 205 | NR | Soft catheter - Edwards-Wallace | Firm catheter - Rocket embryo transfer | FR | NR |  |
| Ghazzawi 1999 (61) | Jordan | 320 | NR | Soft catheter - Edwards-Wallace | Firm catheter - Erlangen metal | FR/FZ | NR |  |
| Grunert 1998 (62) | Spain | 150 | NR | Soft catheter - Edwards-Wallace or Cook | Firm catheter - Frydman DT | FR | NR |  |
| Karande 2002 (63) | USA | 251 | I: IVF-ET from October 2000 to April 2001 | Echo-dense tip catheter (Cook Echotip) | Wallace catheter | FR/FZ | D3 |  |
| Lavery 2001 (64) | UK | 308 | NR | Soft catheter - Edwards-Wallace | Firm catheter - Rocket embryo transfer | FR | NR |  |
| Levi-Setti 2002 (65) | Italy | 200 | I: Age < 37 years, ≥2 type 1 or 2 embryos | Soft catheter - Cook | Firm catheter - Wallace | NR | NR |  |
| Mayer 1999 (66) | USA | 213 | NR | Soft catheter – Cook | Firm catheter - Wallace | NR | NR |  |
| McDonald 2002 (67) | Australia | 650 | I: Age <40, IVF  E: Known uterine abnormality, day 3 FSH >10 IU/l, previous difficult embryo transfer, PGD | Soft catheter - Cook | Firm catheter - Tomcat | FR/FZ | D3 |  |
| McIlveen 2005 (68) | UK | 150 | E: Age >40 years, high basal FSH, a previous difficult ET, >6 previous ETs | Soft catheter - Cook K-jet | Firm catheter - Edwards-Wallace | FR | D2-3 |  |
| Meriano 2000 (69) | Canada | 66 | I: Premenopausal, age 18–38, ≤2 previous IVF cycles, normal uterine cavity on hysterosalpingography or hysteroscopy.  E: Known cervical problem, previous difficult transfer, <2 embryos available | Double lumen catheter - Fryman TDT | Single lumen catheter - Tomcat | FR | D2-3 |  |
| Mortimer 2002 (70) | Canada | 118 | I: <40 years of age, ≤2 previous IVF attempts, sufficient Day 3 | Soft catheter - Cook SIVF 6019 | Firm catheter - Tomcat | FR | D3 |  |
| Ocal 2003 (71) | Turkey | 298 | I: Patients undergoing IVF ET | Straight catheter tip | Bent catheter tip | NR | NR |  |
| Perin 1999 (72) | Brazil | 248 | NR | Soft catheter - Cook Tefcat | Firm catheter -Frydmann Tight difficult Transfer | NR | D2 |  |
| Rhodes 2007 (73) | USA | 99 | I: <40 years of age, BMI 20 –35, fresh sperm or oocytes, ≥3 embryos available for transfer, never had previous ART cycle  E: Gestational carrier | Soft catheter - Cook World | Firm catheter - Edwards-Wallace | FR | D3 |  |
| Ruhlmann 2015 (74) | Argentina | 703 | I: IVF or ICSI within a 30-month period  E: Lack of retrieved mature oocytes; failed complete fertilization or cleavage; no fresh sperm available; endometrium unfit for ET; extreme difficulty or impossibility when performing the mock ET; or cycle cancellation to prevent OHSS | Soft catheter - Frydman Ultrasoft | Firm catheter - Frydman classic | NR | NR |  |
| Saldeen 2008 (75) | Egypt & Sweden | 391 | I: Age <40 years, ≥2 embryos generated from a fresh IVF cycle on the day of ET | Soft catheter - Cook Sydney SIVF | Firm catheter - Edwards-Wallace | FR | NR |  |
| Talwar 2011 (76) | India | 1446 | I: Infertile patients undergoing IVF after OPU | Soft catheter - Cook | Firm catheter - Frydman classic | NR | D2-3 |  |
| Van Weering 2002 (77) | Netherlands | 1296 | I: All patients eligible for embryo transfer  E: Cryopreserved embryos, participation in another study protocol | Soft catheter - Cook K-soft 5000 | Firm catheter - Tight difficult transfer | FR | D2-5 |  |
| Wisanto 1989 (78) | Belgium | 400 | I: Women undergoing IVF at the centre | Soft catheter - Edwards-Wallace or Frydman | Firm catheter - Tight difficult transfer | FR | D3 |  |
| Yao 2009 (79) | Belgium | 1446 | I: Patients treated in the centre | Cook K-SOFT-5100 | Frydman classical catheters 4.5 | NR | D2-3 |  |
| CHARACTERISTICS OF INCLUDED STUDIES EVALUATING THE EFFECTS OF A FULL BLADDER DURING EMBRYO TRANSFER ON CLINICAL PREGNANCY RATES | | | | | | | |  |
| Study | **Country** | **Number** | **Inclusion/Exclusion Criteria** | **Intervention** | **Comparison** | **Fresh or Frozen Cycle** | **Stage of Embryo Development** |  |
| Lewin 1997 (80) | Israel | 796 | NR | Full bladder group were asked to urinate, and then drink 1,000 ml of water 1 h prior to the actual ET. | Empty bladder | NR | NR |  |
| Lorusso 2005 (81) | Italy | 131 | I: BMI 18-30 kg/m^2^  E: Uterine or ovarian abnormalities | Full bladder | Empty Bladder | FR | NR |  |
| Mitchell 1989 (82) | UK | 142 | NR | Emptied their bladders 1 hr before ET and then drank 250 ml of fluid. | Emptied bladders immediately before ET | FR | NR |  |
| CHARACTERISTICS OF INCLUDED STUDIES EVALUATING THE EFFECTS OF G-CSF DURING EMBRYO TRANSFER ON CLINICAL PREGNANCY RATES | | | | | | | |  |
| Study | **Country** | **Number** | **Inclusion/Exclusion Criteria** | **Intervention** | **Comparison** | **Fresh or Frozen Cycle** | **Stage of Embryo Development** |  |
| Arefi 2018 (83) | Iran | 52 | I: Age 22-44 years, >2 IVF/ICSI-ET failures despite transfer of ≥2 good-quality embryos in each attempt.  E: hysterosalpingogram abnormalities, thrombophilia, immunological and genetics problems, severe male factor infertility. | The treated group received 300 μg (0.5 ml) recombinant human G-CSF subcutaneously which was injected 30 min before blastocyst embryo transfer. | Control group received standard ET procedure | NR | D5 |  |
| Obidniak 2016* (84) | Russia | 130 | I: RIF defined as ≥2 cycles of IVF with good-quality embryos transferred in each cycle without achieving clinical pregnancy; blastocyst transfer  E: congenital uterine anomalies; Asherman’s syndrome; endometrial thickness <7 mm | 1) study group N1: intrauterine perfusion with G-CSF (filgrastim 30 million IU, 1 mL) using insemination catheter 5 days prior to embryo transfer; 2) study group N2 : G-CSF (filgrastim 30 million IU, 1 ml) was administered subcutaneously once at the day of embryotransfer; | Control group: no therapy | FR | D5 |  |
| Singh 2015 (85) | India | 48 | I: Infertile patients, thin endometrium, age <42 years | The study group received intrauterine administration of G‐CSF (300 mg/ml) before Embryo Transfer in IVF cycles | Control group received placebo‐saline before Embryo Transfer in IVF cycles | NR | NR |  |
| Singh 2018 (86) | India | 102 | NR | The study group received either intrauterine infusion of 300 microgramme/1 ml of G‐CSF, or subcutanious injection of GCSF before ET | Control group underwent either placebo‐saline infusion or placebo subcutanious multivitamin injection before ET | FR | NR |  |
| CHARACTERISTICS OF INCLUDED STUDIES EVALUATING THE EFFECTS OF hCG ADMINISTRATION DURING EMBRYO TRANSFER ON CLINICAL PREGNANCY RATES | | | | | | | |  |
| Study | **Country** | **Number** | **Inclusion/Exclusion Criteria** | **Intervention** | **Comparison** | **Fresh or Frozen Cycle** | **Stage of Embryo Development** |  |
| Aaleyasin 2015 (87) | Iran | 483 | I: Infertile women undergoing IVF/ICSI  E: Age > 40 years; history of percutaneous epididymal sperm aspiration; testicular sperm extraction; myomectomy; hydrosalpinx; uterine fibroma with pressure effect on endometrium; endometriosis; azoospermia | 500 IU hCG in a volume of 50 μL tissue culture media injected into the uterus 5 to 7 minutes before ET | Control: 50 μL tissue culture media instead of hCG | FR | D2-3 |  |
| Cambiaghi 2013 (88) | Brazil | 44 | I: endometrial thickness > 7 mm on day the donor received hCG, ≥2 blastocysts on day of ET | 500 IU intrauterine injection of hCG 6 hours before ET | ET without any pre-intrauterine injection | FR | D5 |  |
| Dehaghani Firouzabadi 2016* (89) | Iran | 159 | I: age 20-40, male factor or unexplained infertility, basal FSH < 12  E: azoospermia; uterine myoma; endometriosis; hydrosalpinges; previous IVF/ICSI trials (successful or unsuccessful); endocrine disease (e.g. diabetes/thyroid dysfunction); hysteroscopic operation due to submucosal myoma; intrauterine synechia | Two study groups: 1000 IU hCG (40 μL) intrauterine injection 7 minutes before ET vs 500 IU hCG (40 μL) | Nothing before ET | FR | D2-3 |  |
| Eskandar 2016 (90) | Saudi Arabia | 240 | I: women undergoing embryo transfer | 500 IU of hCG intrauterine 10 minutes before ET | no pre-intrauterine injection | NR | NR |  |
| Hafezi 2018* (91) | Iran | 180 | I: Primary infertility, first frozen embryo transfer cycle after a failed fresh IVF/ICSI cycle, >1 good embryos (grade A, B)  E: age≥40 years; BMI≥30 kg/m2 , severe male factor infertility (azoospermia, percutaneous epididymal sperm aspiration, and testicular sperm extraction), uterine factor, myomectomy, hydrosalpinx, myoma with pressure effect on endometrium, endometriosis, storage time of freezing >2 years. | Three groups: intrauterine infusion of 40 μL of tissue culture medium containing 500 IU of hCG | Control 1 - endometrial infusion of ET media only vs control 2 – no intrauterine infusion | FZ | NR |  |
| Hong 2014 (92) | USA | 300 | I: Fresh or frozen ET, age <43  E: not simultaneously participating in another prospective clinical trial | Endometrial infusion of 20 μL ET media with 500 IU of purified urinary placental hCG <3 minutes before ET | Control: endometrial infusion of 20 μL ET media only | FR/FZ | D5 |  |
| Hosseini 2016 (93) | Iran | 100 | I: women undergoing assisted reproduction  E: history of uterine surgery such as myomectomy; recurrent miscarriage; hydrosalpinx, endometrioma, or endometriosis | Intrauterine injection of 40 μL of 500 IU hCG 7 mins before ET | ET carried out with no intervention | FZ | D2-5 |  |
| Laokirkkiat 2018 (94) | Thailand | 200 | I: Infertile women, age 18–43 years, fresh and frozen–thawed cycles  E: azoospermic male partner, no oocytes retrieved, no embryos available, endometrial thickness <8 mm, donor oocytes, in vitro oocyte maturation cycles. | 500 IU of hCG in 10 μL culture medium infused into the uterine cavity using a soft catheter 4 min before ET | Control - 10 μL of culture medium alone | FR/FZ | D3-5 |  |
| Leao 2013 (95) | Brazil | 36 | I: ≥2 previous failures in IVF cycles | intrauterine injection of hCG 500 IU 6 hours before ET | No intervention | NR | NR |  |
| Mansour 2011a* (96) | Egypt | 260 | I: Age <40 years, infertility due to male factor  E: previous IVF/ICSI trials, azoospermia; uterine myoma, previous myomectomy; endometriosis; hydrosalpinges | Two study groups: 40 μL of tissue culture medium containing hCG 100 IU injected intrauterine 7 minutes before ET vs 200 IU hCG | No intrauterine hCG injection before ET | FR | D2-3 |  |
| Mansour 2011b (96) | Egypt | 212 | I: Age <40 years, infertility due to male factor  E: previous IVF/ICSI trials, azoospermia; uterine myoma, previous myomectomy; endometriosis; hydrosalpinges | 40 μL of tissue culture medium containing hCG 500 IU injected intrauterine 7 minutes before ET | No intrauterine hCG injection before ET | FR | D2-3 |  |
| Mostajeram 2017 (97) | Iran | 100 | I: age 20-40 years, BMI 18-30 kg/m^2^, infertile due to male factor, regular menstrual cycle of 24 to 35 days, ovulatory  E: PCOS, uterine pathologies, endometriosis, hydrosalpinges, any endocrine disease, chronic systemic illness; azoospermia; previous successful IVF or ICSI | Injection of 700 IU of intrauterine hCG 10 minutes before embryo transfer | No hCG before embryo transfer | FZ | D5 |  |
| Santibañez 2014 (98) | Mexico | 210 | I: infertile women age < 40 years undergoing IVF/ICSI  E: azoospermia | 20 μL of embryo culture medium containing hCG 500 IU administered intrauterine before ET | No intrauterine hCG | FR/FZ | D2-3 |  |
| Singh 2014 (99) | India | 216 | I: infertile women aged < 42 years recurrent implantation failure | Intrauterine administration of rhCG 500 IU in 40 μL 5 minutes before ET | Culture medium administered only before ET | NR | D2-3 |  |
| Wang 2019 (100) | China | 137 | I: RIF, Age <40 years, regular menstruation (cycle 25~35 d),  E: Postoperative uterine cavity sticking, tubal effusion, endocrine diseases, endometriosis. Uterine abnormalities: intermural fibroids (≥3 cm), submucosal muscles tumours, adenomyomas, endometrial polyps, abnormal endometrial hyperplasia, uterine cavity adhesion and abnormal uterine cavity shape; hydrosalpinx, endometriosis, chromosomal abnormalities in either or both of the couple; blastocyst stage embryos, PGD | 500 IU hCG intrauterine perfusion - 40 μL of which was injected at an intrauterine site at 3 minutes before embryo transfer | No hCG | FZ | NR |  |
| Wirleitner 2015a (101) | Austria | 1004 | I: fresh blastocyst transfer on day 5; age ≤ 43 years  E: oocyte donation cycles; RIF (≥ 3 negative IVF cycles) | Intrauterine hCG 500 IU in 40 μL embryo culture medium G-2 PLUS administered on either 2 days before or 3 mins before ET | 40 μL culture medium without hCG at the same times | FR | D2-5 |  |
| Wirleitner 2015b (102) | Austria | 480 | I: Age 38-43 years  E: RIF | Intrauterine hCG 500 IU dissolved in 40 μL embryo culture medium administered 3 minutes before ET | 40 μL culture medium without hCG 3 minutes before ET | FR | D5 |  |
| Zarei 2014 (103) | Iran | 210 | I: age 18-40 years, infertility  E: Autoimmune disorders, endocrinopathies, previous successful IVF/ICSI trials; endometriosis; azoospermia; hydrosalpinges | rhCG 250 μg (0.5 mL, 6500 IU) administered through intrauterine injection 12 minutes before ET | intrauterine injection of normal saline (0.5 mL) 12 minutes before ET | NR | D2-3 |  |
| CHARACTERISTICS OF INCLUDED STUDIES EVALUATING THE EFFECTS OF A HYALURONIC ACID ADMINISTRATION BEFORE EMBRYO TRANSFER ON CLINICAL PREGNANCY RATES | | | | | | | |  |
| Study | **Country** | **Number** | **Inclusion/Exclusion Criteria** | **Intervention** | **Comparison** | **Fresh or Frozen Cycle** | **Stage of Embryo Development** |  |
| Balaban 2004 (104) | Turkey | 386 | I: Blastocyst stage embryos  E: None | ET in EmbryoGlue (0.5 mg/mL HA) 30 mins prior to transfer | ET in G2.3 (0.125mg/mL HA). | FR | D5 |  |
| Dittmann-Muller 2009 (105) | Germany | 102 | I: IVF or ICSI between January 2006 and March 2007.  E: None | ET in EmbryoGlue (0.5 mg/mL HA) 30 minutes prior to transfer | Transfer in G-2 (0.125 mg/mL HA). | FR | D3 |  |
| Fancsovits 2011 (106) | Hungary | 200 | I: None  E: None | ET in EmbryoGlue (0.5 mg/mL HA) 5 to 10 minutes before transfer. | Transfer in G-2 (0.125 mg/mL HA) | FR | D3 |  |
| Fancsovits 2015 (107) | Hungary | 409 | I: IVF or ICSI between January 2010 and August 2012, only 1 treatment cycle  E: Oocyte donation | ET in EmbryoGlue (0.5 mg/mL HA) 5-10 minutes before transfer. | Transfer in G-2 (0.125 mg/mL HA) | FR | D3 |  |
| Fasano 2016 (108) | Belgium | 328 | I: NR  E: NR | ET in EmbryoGlue (0.5% mg/mL HA) | Routine ET medium | FR | D3-5 |  |
| Feichtinger 1992 (109) | Austria | 546 | I: Age 23-39 years  E: age >39 | Fibrin sealant: 20 /il of thrombin solution (500 IU/ml) and 20 /J of dissolved protein-fibrinogen concentrate | Conventional ET | NR | NR |  |
| Friedler 2005 (110) | Israel | 187 | I: Age <43 years, IVF or ICSI, failed to achieve pregnancy after 4 previous embryo transfers  E: Age >43 years, more or less than 4 previous treatment cycles | ET with EmbryoGlue (0.5 mg/mL HA) | HTF medium enriched with 20% serum substitute supplement (SSS) | NR | D2-4 |  |
| Friedler 2007 (111) | Israel | 101 | I: Failed to achieve ongoing pregnancy after >4 previous embryo transfers in which 2-4 embryos were transferred, including at least one optimal embryo. Age <43 years, given informed consent.  E: Age >43 years, systemic disease, BMI > 29 kg/m^2^, uterine malformation, evidence of low ovarian response, elevated baseline FSH (>12 IU/L), hydrosalpinx, participation in any other clinical study | ET in EmbryoGlue (0.5 mg/mL HA and 2.5 mg/mL recombinant human albumin) for 10 minutes | ET in HTF with gentamycin, enriched with 20% serum substitute supplement. | NR | D2-3 |  |
| Kandari 2019 (112) | India | 321 | I: Time lapse selected; single, FR embryo transfers in patients with PCOS | Embryo transfer in EmbryoGlue | CSCM medium (containing no HA) | FR | D3-5 |  |
| Korosec 2007 (113) | Slovenia and Austria | 296 | I: Age <37, <3 treatment cycles | Fresh and frozen-thawed embryo transfers in EmbryoGlue (0.5 mg/mL HA) | M2 medium | FR/FZ | D5 |  |
| Mahani 2007 (114) | Iran | 60 | I: Age ≤35, ≥3 embryos suitable for transfer, no previous IVF/ICSI cycles | Embryo transfers in EmbryoGlue (0.5 mg/mL HA), exposed for 10 minutes prior to transfer | standard medium containing 20% albumin | FR | D3 |  |
| Morbeck 2007 (115) | USA | 83 | I: Frozen thaw embryo transfers. Men aged >18, women aged 18-42 (if using their own oocytes and embryos frozen before 39 years old) or 18-50 if using donor oocytes  E: Prior participation to this study. Blastocyst, single embryos. Prior embryo transfer with large amount of blood on the outside of the catheter. ≥3 previous treatment failures | ET in EmbryoGlue (0.5 mg/mL HA), exposed for an average of 15 minutes prior to transfer | Transfer in G2 culture medium (0.125 mg/mL HA). | FZ | D3 |  |
| Perez 2019 (116) | USA | 119 | NR | Treatment groups consisted of one-hour, two-hour and three-hour post thaw exposure to EmbryoGlue! before ET | Embryo glue following the manufacture’s guidelines. | FZ | D5 |  |
| Ravhon 2005 (117) | Israel | 148 | I: None  E: None | FR embryo transfers in EmbryoGlue (0.5 mg/mL HA) | FR transfers in G1 medium (0.125 mg/ mL HA) | FR | NR |  |
| Schoolcraft 2002 (118) | USA | 175 | I: Both IVF patients with their own oocytes and oocyte donors were included | G2.3 medium supplemented with EmbryoGlue (0.5 mg/mL HA) | transfer in G2.3 medium (0.125 mg/mL HA) | NR | D3 |  |
| Simon 2003 (119) | Israel | 80 | I: Age ≤35 years, ≥3 embryos suitable for transfer, ≤3 previous treatment failures  E: None | Embryo transfers in culture medium supplemented with 0.5 mg/mL HA, exposed for 5 to 10 minutes prior to ET | Culture medium | NR | D3 |  |
| Tomari 2014 (120) | Japan | 736 | I: None  E: None | EG for more than 30 min before intrauterine transfer. | No HA | FR/FZ | D 2-3 |  |
| Urman 2008 (121) | Turkey | 1282 | I: Treatment cycles reaching embryo transfer, fresh, IVF/ICSI, participant's own oocytes | ET in EmbryoGlue (0.5 mg/mL HA) | G2 version 3 (0.125 mg/mL HA) supplemented with HSA (human serum albumin) | FR | D3-5 |  |
| Valojerdi 2006 (122) | Iran | 815 | I: None  E: None | EmbryoGlue prior to the transfer | No treatment | FR | D3 |  |
| Walker 2005 (123) | UK | 68 | I: Age <39  E: Prior participation in this study, blastocyst embryos, single embryo transfer, prior embryo transfer with a large amount of blood on the catheter, ≥3 consecutive failed embryo transfers. | ET in EmbryoGlue (0.5 mg/mL HA) | Transfer in G1 version 3 (0.125 mg/mL HA) | FZ | NR |  |
| Yakin 2005 (124) | Turkey | 129 | NR | ET in EmbryoGlue (0.5 mg/mL HA) | G2 version 3 culture medium (0.125 mg/ mL HA) | FZ | D3 |  |
| Yung 2021 (125) | China | 550 | I: Age <43,frozen-thawed embryo transfer E: Donor oocyte/embryo treatment, PGD | Hyaluronan-enriched embryo transfer medium (HETM) (hyaluronan concentration 0.5 mg/mL) | Conventional medium (HA concentration 0.125 mg/mL) | FZ | D3-6 | |
| CHARACTERISTICS OF INCLUDED STUDIES EVALUATING THE EFFECTS OF NSAIDS DURING EMBRYO TRANSFER ON CLINICAL PREGNANCY RATES | | | | | | | | |
| Study | **Country** | **Number** | **Inclusion/Exclusion Criteria** | **Intervention** | **Comparison** | **Fresh or Frozen Cycle** | **Stage of Embryo Development** |  |
| Bernabeu 2006 (126) | Spain | 136 | I: First cycle, Oocyte recipients, IVF and ICSI, no known allergic reaction to NSAIDs, no neurological or gastrointestinal disease.  E: Recurrent miscarriage, endometrial pathology, severe endometriosis. | Indomethacin - three doses of 100mg of indomethacin rectally every 12h, starting on the night prior to the transfer | No indomethacin | NR | D2-3 | |
| Dal Prato 2009 (127) | Italy | 200 | I: age <44 years; regular ovulatory menstrual cycles of 25–33 days; infertility caused by tubal, idiopathic or male factors or endometriosis; ≤2 previous ETs  E: FSH concentrations >15 IU/l on day 3 of menstrual cycle, previously shown poor response to gonadotrophins, cryptozoospermia requiring testicular sperm aspiration | Treatment group patients received a single oral dose of 10 mg of piroxicam 1–2 h before embryo transfer. | Control group received standard treatment. | FR | D2 | |
| Duvan 2006* (128) | Turkey | 187 | I: Non-selected patients, first ICSI cycle  E: conditions contraindicated to anti-aggregants and/or steroid administrations | Four groups: A. Aspirin 100 mg/day; B. Aspirin 100 mg þ prednisone 10 mg/day; C. Prednisone 10 mg; | D. Placebo | FR | D5 | |
| Fekih 2013 (129) | Tunisia | 166 | I: IVF due to tubal, male infertility, unexplained, or endometriosis factors. | Ibuprofen - an oral dose of 200mg ofibuprophen (10 drops) 90minutes before embryo transfer | Placebo 90 mins before ET | FR | D5-6 | |
| Firouzabadi 2007 (130) | Iran | 180 | I: IVF due to tubal, ovarian, male infertility unexplained and mixed factor | The treatment group received an oral dose of piroxicam 1-2 hours before ET | The control group received a placebo, 1-2 hours before ET | FR | D2 | |
| Moon 2004 (131) | Korea | 266 | I: IVF because of tubal, male infertility, unexplained, or endometriosis factor. | In the treatment group, patients received an oral dose of 10 mg of piroxicam 1-2 hours before ET. | The control group were treated with placebo. | FR/FZ | D5 | |
| Zarei 2020 (132) | Iran | 178 | I: Age <42 years, infertility due to male factors or tubal factors, ovulation disorders, endometriosis and unexplained, normal uterine anatomy  E: donated eggs, structural uterine abnormalities, malignancies, autoimmune or other serious medical illnesses. | Patients received either single dose of piroxicam 1–2 h before frozen–thawed ET | Placebo at the same time. | FZ | D2-5 | |
| CHARACTERISTICS OF INCLUDED STUDIES EVALUATING THE EFFECTS OF A NURSE VS A DOCTOR PERFORMING THE EMBRYO TRANSFER ON CLINICAL PREGNANCY RATES | | | | | | | | |
| Study | **Country** | **Number** | **Inclusion/Exclusion Criteria** | **Intervention** | **Comparison** | **Fresh or Frozen Cycle** | **Stage of Embryo Development** | |
| Bjuresten 2003 (133) | Sweden | 102 | NR | ET by a midwife | ET by a gynaecologist. | FR | D2-3 | |
| Rinaldi 2014 (134) | Italy | 553 | E: Decreased ovarian reserve with FSH level >15 IU, surgically retrieved spermatozoa | A midwife (with no experience in ultrasound) to provide UGET assistance | UGET assistance by a trained gynaecologist | NR | NR | |
| CHARACTERISTICS OF INCLUDED STUDIES EVALUATING THE EFFECTS OF RELAXATION OR MINDFULNESS ON CLINICAL PREGNANCY RATES | | | | | | | | |
| Study | **Country** | **Number** | **Inclusion/Exclusion Criteria** | **Intervention** | **Comparison** | **Fresh or Frozen Cycle** | **Stage of Embryo Development** | |
| Aba 2017 (135) | Turkey | 186 | I: Age 20-25 years, primary infertility for at least 1 year, IVF-ET, able to speak Turkish, literate, agree to participate in the study  E: Secondary infertility, physical or mental illness, hearing impairments, perception disorders, communication problems. | 28 minutes of music therapy was applied to the music therapy group 1 h before the embryo transfer and after the embryo transfer. | Routine monitoring and treatment protocols were applied before and after the embryo transfer | NR | D2-3 | |
| Benson 2006 (2) | USA | 100 | NR | Relaxation group (part of a larger acupuncture study) | No intervention | NR | D3-5 | |
| Fratterelli 2008 (7) | USA | 400 | NR | Relaxation group (part of a larger acupuncture study) | No intervention | NR | NR | |
| Gavrizi 2019 (136) | USA | 65 | I: Undergoing SET of autologous PGT-A euploid embryo in controlled FET cycles E: ≥2 failed FETs, uterine anomaly, prior uterine surgery, already undergoing massage or acupuncture therapy. | Massage therapy - standardized 20-minute massage by one therapist beginning 45 minutes prior to SET | Standard care without massage | FZ | NR | |
| MacLennan 1985 (137) | Australia | 96 | NR | Patients received 2 mg purified porcine relaxin. The appropriate gel was placed in the posterior vaginal fornix by means of a soft latex catheter and a syringe immediately after embryo transfer and again 3 days later. | Placebo (distilled water) mixed with 300 mg of water-soluble cellulose (Tylose) granules (Hoechst) to make a viscous gel. | NR | NR | |
| Murphy 2014 (138) | USA | 181 | I: Subfertile women aged 21-44, IVF  E: Already enrolled in other IVF-ET clinical trials, PGD | Harp therapy | Standard therapy | NR | NR | |
| Shaker 1993 (139) | UK | 120 | I: first IVF/embryo transfer treatment, informed consent. | Patients were allocated to receive two sublingual spray emissions of either GTN (400 /tg/spray) or placebo, approximately 3 min before embryo transfer. | Two sublingual spray emissions of placebo | NR | NR | |
| Stocker 2016 (140) | UK | 42 | I: Age 25-40 years, IVF or ICSI, fresh or frozen embryos  E: Women unable to consent, or with hearing impairment. | Underwent the ET procedure whilst listening to self-selected music with no restrictions to the type of music they could select, through headphones, and listened for a further 15 minutes in the recovery room. | Did not listen to music in the ET procedure or in the following 15 minutes. | FR/FZ | D2-5 | |
| CHARACTERISTICS OF INCLUDED STUDIES EVALUATING THE EFFECTS OF SEMINAL FLUID APPLICATION DURING EMBRYO TRANSFER ON CLINICAL PREGNANCY RATES | | | | | | | | |
| Study | **Country** | **Number** | **Inclusion/Exclusion Criteria** | **Intervention** | **Comparison** | **Fresh or Frozen Cycle** | **Stage of Embryo Development** | |
| Aflatoonian 2009 (141) | Iran | 385 | I: couples undergoing assisted reproduction, >5 years of subfertility  E: none stated | Intervention: vaginal intercourse at least once during the 12 hours following embryo transfer. | Abstinence during the entire ART cycle | FR | D2-3 | |
| Karimian 2010 (142) | Iran | 569 | I: women undergoing ART | Intercourse | Abstinence | NR | NR | |
| CHARACTERISTICS OF INCLUDED STUDIES EVALUATING THE EFFECTS OF THE SITE OF EMBRYO DEPOSITION ON CLINICAL PREGNANCY RATES | | | | | | | | |
| Study | **Country** | **Number** | **Inclusion/Exclusion Criteria** | **Intervention** | **Comparison** | **Fresh or Frozen Cycle** | **Stage of Embryo Development** | |
| Coroleu 2002 (143) | Spain | 180 | NR | Two study groups: (1) 10.2+/-0.9mm from fundus, (2) 14.6+/-0.7mm from fundus | 19.3+/-0.8mm from fundus | FR | D2-3 | |
| Franco 2004 (144) | Brazil | 400 | E: Frozen cycles | Intention - <50% endometrial cavity length from fundus,  Actual - 12.3+/-1.6mm from fundus | Intention - >50% endometrial cavity length from fundus, Actual - 18.3+/-3.2mm from fundus | FR | D3 | |
| Nazari 1993 (145) | USA | 1590 | NR | <5m from fundus | >15mm from fundus | FR/FZ | NR | |
| CHARACTERISTICS OF INCLUDED STUDIES EVALUATING THE EFFECTS OF ULTRASOUND GUIDED EMBRYO TRANSFER ON CLINICAL PREGNANCY RATES | | | | | | | | |
| Study | **Country** | **Number** | **Inclusion/Exclusion Criteria** | **Intervention** | **Comparison** | **Fresh or Frozen Cycle** | **Stage of Embryo Development** | |
| Abdelmassih 2001 (146) | Brazil | 109 | NR | TA ultrasound guided ET | Clinical touch | FR | NR | |
| Ammar 2013 (147) | Egypt | 90 | NR | TA ultrasound guided ET | Clinical touch | FR | D2 | |
| Azmy 2009 (148) | Egypt | 853 | I: < 38 years, ≤ BMI 30 kg/m^2^, basal FSH ≤ 12 IU/mL, embryo quality ≥ grade B on D3, number of fresh embryos replaced ≤ 3 | TA ultrasound guided ET | Clinical touch | FR | NR | |
| Bar Hava 2003 (149) | NR | 131 | NR | TA ultrasound guided ET | Clinical touch | FR | NR | |
| Bodri 2011 (150) | Spain | 329 | I: Fresh ET, two D2-3 embryos  E: Black recipients, Turner syndrome, uterine malformations/fibroids | TA ultrasound guided ET | TV ultrasound guided ET | FR | D2-3 | |
| Chen 2007 (151) | Taiwan | 50 | I: Grade 2 or better embryo, 8 cell stage  E: Age ≥40 years, cleaved embryos with <8 cells; three previous failed assisted conception cycles; anticipated difficulty with ET | TA ultrasound guided ET | Clinical touch | FR | D3 | |
| Coroleu 2000 (152) | Spain | 362 | I: Patients from our IVF–embryo transfer treatment programme | TA ultrasound guided ET | Clinical touch | FR | D2-6 | |
| Coroleu 2002 (153) | Spain | 184 | I: Aged 30–44 years, IVF, frozen embryos  E: Oocyte donation cycles | TA Ultrasound guided ET | Clinical touch | FZ | NR | |
| Dalal 2014 (154) | India | 354 | NR | TV ultrasound guided ET | TA ultrasound guided ET | NR | NR | |
| Davar 2007 (155) | Iran | 180 | NR | TA ultrasound guided ET | Clinical touch | NR | NR | |
| de Camargo Martins 2004 (156) | Brazil | 100 | NR | TA ultrasound guided ET | Clinical touch | FR | NR | |
| Deep 2013 (157) | China | 208 | NR | TA ultrasound guided ET | TV ultrasound guided ET | FR | NR | |
| Drakeley 2008 (158) | UK | 1649 | I: IVF or ICSI at the Hewitt centre | TA ultrasound guided ET | Clinical touch | FR/FZ | D2 | |
| Eskandar 2008 (159) | Saudi Arabia | 373 | I: Women undergoing ET in a fresh cycle and with good-quality embryos | TA ultrasound guided ET | Clinical touch | FR | D3 | |
| Garcia-Velasco 2001 (160) | Spain | 215 | NR | TA ultrasound guided ET | Clinical touch | FR | D2-6 | |
| Garcia-Velasco 2002 (161) | Spain | 374 | I: Part of oocyte donation program, good quality embryos | TA ultrasound guided ET | Clinical touch | FR | D2-6 | |
| Hauzman 2013 (162) | Spain | 97 | I: Egg donation with ET with two embryos on D3  E: Submucous or >3 cm intramural fibroids, Turner syndrome patients, and black recipients | TA ultrasound guided ET | TV ultrasound guided ET | NR | D3 | |
| Kan 1999 (163) | UK | 195 | I: ET following IVF  E: Age >42 years old, >3 previous assisted conception cycles, previous difficult or anticipated difficult embryo transfer, general anaesthesia for the patient | TA ultrasound guided ET | Clinical touch | FR | NR | |
| Karavani 2017 (164) | Israel | 120 | I: Cleavage-stage fresh ET in patients 18-40 years old  E: BMI>35 kg/m^2^, PGD cases, oocyte donation, and blastocyst transfer | TA ultrasound guided ET | TV ultrasound guided ET | FR | D2-3 | |
| Kosmas 2007 (165) | Belgium | 300 | I: Fresh embryos, on D3 or 5. BMI of 20–30kg/m^2^  E: >40 years old, previous cervical surgery for cervical intraepithelial neoplasia (CIN) | TA ultrasound guided ET using an echo-sensitive catheter (K-J-SPPE echo tip, Cook) | Clinical touch using the K-Soft Cook catheter | FR | NR | |
| Li 2005 (166) | China | 330 | I: Infertile patients, age 28-41 | TA ultrasound guided ET | Clinical touch | FR | D2-3 | |
| Maldonado 2005 (167) | Brazil | 26 | NR | TA ultrasound guided ET without previous hysterosonometry | Hysterosonometry performed without ultrasound guidance. | FR | NR | |
| Marconi 2003 (168) | Argentina | 83 | I: <38 years of age | TA ultrasound guided ET | Clinical touch | FR | D3 | |
| Matorras 2002 (169) | Spain | 515 | I: <40 years  E: Frozen, embryos oocyte donation, ICSI | TA ultrasound guided ET | Clinical touch | FR | D2-6 | |
| Moraga-Sanchez 2004 (170) | Mexico | 67 | NR | TA ultrasound guided ET | Clinical touch | NR | NR | |
| Porat 2010 (171) | USA | 186 | I: Autologous IVF, oocyte donation, and frozen embryo transfer  E: Cases of severe OHSS requiring cryopreservation; gestational surrogate cycles; inadequate visualization during a mock-ET. | TA ultrasound guided ET | TV ultrasound guided ET | FR/FZ | D3-6 | |
| Prapas 1995 (172) | Greece | 132 | NR | TA ultrasound guided ET | Clinical touch | NR | NR | |
| Revelli 2016 (173) | Italy | 1648 | I: IVF at our unit, age < 43 years, consent to procedure  E: Age ≥ 43 years, uterine abnormalities (e.g., malformations, myomas, endometrial polyps), blastocyst, necessary to change the catheter during ET for cervical stenosis | TA ultrasound guided ET | TV ultrasound guided ET | FR | D2-3 | |
| Sallam 2002 (174) | Egypt | 640 | E: Cryopreserved embryos, general anaesthesia | TA ultrasound guided ET | Clinical touch | FR | NR | |
| Saravelos 2016 (175) | Hong Kong | 474 | I: All women undergoing US-guided ET  E: Age ≥42 years, endometrial cavity not adequately visualised on ultrasound | 3D TA ultrasound guided ET | 2D TA ultrasound guided ET | FR/FZ | D3-5 | |
| Tang 2001 (176) | Hong Kong | 800 | I: IVF, ICSI  E: General anaesthesia, refusal to participate | TA ultrasound guided ET | Clinical touch | FR/FZ | NR | |
| Weissman 2003 (177) | Israel | 155 | I: IVF-ET between 07/02 and 2/03 | TA ultrasound guided ET | Clinical touch | FR | D2-3 | |
| Wisanto 1989 (78) | Belgium | 196 | I: IVF-ET from July 1987 to March 1988,  E: NR | TA ultrasound guided ET | Clinical touch | NR | NR | |
| CHARACTERISTICS OF INCLUDED STUDIES EVALUATING THE EFFECTS OF MISCELLANEOUS INTERVENTIONS DURING EMBRYO TRANSFER ON CLINICAL PREGNANCY RATES | | | | | | | | |
| Study | **Country** | **Number** | **Inclusion/Exclusion Criteria** | **Intervention** | **Comparison** | **Fresh or Frozen Cycle** | **Stage of Embryo Development** | |
| Yayla Abide 2018 (178) | Turkey | 2000 | I: Age 20-40, grade 1 embryos at cleavage or blastocyst stage, easy ET, first, fresh cycle single ET  E: Age >40 years, age <20 years, uterine anatomical abnormalities (e.g., malformations, myomas, endometrial polyps), poor ovarian reserve, low-quality embryos, difficult ET, and frozen cycle transfer, endometriosis. | Catheter rotation (360 degrees) | ET with no further manipulation, the catheter was pulled back slowly and without rotation | FR | D2-5 | |
| Sigalos 2017 (179) | Greece | 236 | I: age ≤ 45 years old, BMI ≤ 36 kg/m2, baseline FSH concentration < 15 IU/l, ovarian stimulation with the same GnRH antagonist protocol, normal uterine cavity with endometrium thickness > 7 mm, and trilaminar morphology at the time of transfer and semen parameters of > 1 × 106/ml motile spermatozoa with > 4% physiological morphology (according to 5th edition of WHO laboratory manual for the examination and processing of human semen)  E: PGD, frozen–thaw, natural cycles or patients following different ovarian stimulation protocol | ET with a low volume (20–25 μl) of culture medium | ET with a high volume (40–45 μl) of culture medium | FR | D3 | |
| Catoire 2013 (180) | France | 93 | I: Age 18-38, first ET  E: Refusal to participate, psychiatric disorder, usage of sedatives or tranquilizers, uterus malformation, contraindication to pregnancy, oocyte donation, frozen oocytes. | Benzodiazepine group received 10mg of diazepam | Hypnosis group received a placebo (lactose). | FR | NR | |
| Ng 2019 (181) | Australia | 93 | I: Age 18-45 years, IVF/ICSI, fresh or frozen–thawed ET, Baseline BP ≥100/60mmHg measured pre-embryo transfer, informed consent.  E: BMI >38, Early follicular phase (day 2–4) serum FSH level >20mIU/mL, abnormal uterine cavity, any contraindication to pregnancy, contraindication for nifedipine, estrogen, or progesterone suppositories, other drugs that interact with cytochrome P450 activity: azole antifungals, cimetidine, cyclosporine, erythromycin, quinidine, terfenadine, warfarin, benzodiazepines, flecainide, imipramine, propafenone, and theophylline; irregular heart beat, on hypertension medication, administration of any investigational drugs within 3 months prior to study, patient not able to communicate adequately with investigators, previous entry into the study, PGD | Nifedipine 30 minutes prior to ET | Placebo 30 mins prior | FR/FZ | NR | |
| Hannoun 2009 (182) | Lebanon | 712 | I: IVF cases reaching ET, indications for IVF treatment included patients with male factor infertility, tubal factor, endometriosis, and unexplained infertility.  E: None | ET performed wearing powdered gloves | ET performed while wearing un-powdered gloves. | FR | D2 | |
| Obidniak 2017 (183) | Russia | 90 | I: RIF, normal karyotype, absence of uterine factors of infertility, absence of chromosomal abnormalities in previous pregnancy | Underwent ET with intrauterine infusion of 2.0ml of autologous PRP | Underwent ET without intrauterine administration | FZ | NR | |
| Mansour 2004 (184) | Egypt | 639 | I: First ICSI trial for male factor infertility, age <39 years old, normal hormonal profile, no pelvic pathology.  E: Azoospermic patients requiring surgical retrieval of spermatozoa | Screw of the vaginal speculum was loosened in order to exert a gentle pressure on the portiovaginalis of the cervix before ejecting embryos and was maintained for 7 min afterwards. | No pressure was applied on the cervix | FR | D2-3 | |
| Amui 2011 (185) | USA | 77 | NR | Loosen the screw of the vaginal speculum to exert gentle pressure on the portiovaginalis of the cervix for 7 mins. | Conventional transfer | NR | D3 | |
| Caanen 2016 (186) | Netherlands | 599 | I: None  E: In other IVF/ ICSI-related research trials, patients with intra-uterine pathology, surrogacy or egg donation cycles | Transfer using the pump regulated embryo transfer (PRET) device | Manual ET technique | FR/FZ | NR | |
| Groutz 1997 (187) | Israel | 40 | I: Patients who failed to conceive after at least three conventional IVF-ET cycles, cervical stenosis. | Ultrasound-guided TV, transmyometrial ET | Transcervical ET. | FR | NR | |
| Abu-Musa 2008 (188) | Lebanon | 125 | I: First IVF cycle between June and December 2006, tubal, male- factor infertility, unexplained, PCOS, or endometriosis factors.  E: None | 17a-hydroxyprogesterone caproate (17-HPC) (250 mg, IM), 1 day before ET. | No injections | FR | D2 | |

*****ET: embryo transfer, AC: acupuncture, FR : fresh embryo transfer, FZ, frozen embryo transfer, NR : Not reported, IVF: in-vitro fertilisation, ICSI: intracytoplasmic sperm injection, BMI: body mass index

**Supplementary Table SII:** List of excluded studies

| Study | Reason for Exclusion |
| --- | --- |
| Abou-Setta | Systematic review |
| Abou-Setta 2005 | Systematic review |
| Abou-Setta 2006 | Systematic review |
| Abou-Setta 2007 | Systematic review |
| Abou-Setta 2007 | Systematic review |
| Agha-Hosseini 2010 | Unsuitable intervention – zygote intrafallopian transfer |
| Aly 2018 | Duplicate |
| Aly 2019 | No outcomes of interest |
| Amui 2011 | Too little information |
| Angik 2018 | Not an RCT/randomised |
| Anonymous 2018 | No outcomes of interest |
| Badenhnoosh 2014 | No outcomes of interest |
| Balmaceda 1992 | Difficult to compare with other studies |
| Ben-Rafael 1995 | Unsuitable intervention – started too early |
| Bhat 2014 | No outcomes of interest |
| Bontekoe 2014 | Systematic review |
| Brown 2007 | Systematic review |
| Brown 2010 | Systematic review |
| Brown 2016 | Systematic review |
| Buckett 2003 | Systematic review |
| Buckett 2015 | Systematic review |
| Caglar 2017 | Not an RCT |
| Cenksoy 2014 | Not an RCT |
| Cha 2001 | Unsuitable intervention – started too early |
| Chen 2015 | Unsuitable intervention - started too early |
| Cheung 2017 | No outcomes of interest |
| Coyle 2020 | Systematic review |
| Cozzolino 2018 | Systematic review |
| Craicunas 2014 | Systematic review |
| Craicunas 2017 | Systematic review |
| Dalton-Brewer 2009 | No outcomes of interest |
| Dehghani 2014 | Duplicate |
| Dehghani 2014 | Duplicate |
| Drakeley 2015 | Duplicate |
| Drew 2014 | No n for groups |
| Dutta 2017 | No outcomes of interest |
| Dutta 2018 | Duplicate |
| Eftekhar 2016 | Unsuitable intervention – started too early |
| Feichtinger 1990 | Not an RCT |
| Fluker 1993 | Unsuitable intervention – zygote intrafallopian transfer |
| Frishman 2007 | No outcomes of interest |
| Gao 2019 | Systematic review |
| Goto 2007 | Unsuitable intervention – started too early |
| Goto 2009 | Unsuitable intervention – started too early |
| Gu 2019 | Systematic review |
| Guven 2020 | Unsuitable intervention – started too early |
| Habana 2001 | Unsuitable intervention – zygote intrafallopian transfer |
| Hebisha 2018 | Duplicate |
| Hong 2013 | Duplicate |
| Huang 2016 | Unsuitable intervention – started too early |
| Huang 2017 | Unsuitable intervention – started too early |
| Janati 2013 | Duplicate |
| Khan 1991 | Unsuitable intervention – started too early |
| Kokkali 2014 | No outcomes of interest |
| Kroon 2012 | Systematic review |
| Kuwahara 2013 | Unsuitable intervention – started too early |
| Kwon 2015 | Difficult to compare with other studies |
| Leeton 1987 | Unsuitable intervention – gamete intrafallopian transfer |
| Li 2004 | Duplicate |
| Li 2004 | Duplicate |
| Llavador-Guerrero 2016 | No number of enrolled participants per groups |
| Manheimer 2013 | Systematic review |
| Mansour 2011 | Duplicate |
| Moraloglu 2010 | Duplicate |
| Morin 2017 | Duplicate |
| Murtinger | Not an RCT |
| Nakagawa 2011 | Duplicate |
| Nakagawa 2012 | No outcomes of interest |
| Nakagawa 2012 | Duplicate |
| Ng 2010 | Duplicate |
| Ng 2017 | Duplicate |
| Nyboe Andersen 2011 | Unsuitable intervention – started too early |
| Oliviera 2004 | Duplicate |
| Ozdamar 2018 | Duplicate |
| Phy 2017 | No outcomes of interest |
| Prapas 1995 | Duplicate |
| Prapas 2012 | Unsuitable intervention – started too early |
| Preutthipan 1994 | Unsuitable intervention – zygote intrafallopian transfer |
| Qu 2019 | No outcomes of interest |
| Saharkhiz 2014 | Difficult to compare with other studies |
| Sallam 2003 | Systematic review |
| Sallam 2011 | Duplicate |
| Sallam 2015 | Systematic review |
| Schwarze 2020 | Systematic review |
| Seminal Fluid |  |
| Seto 2016 | Not an RCT |
| Shamonki 2005 | Not an RCT |
| Shen 2015 | Systematic review |
| Su 2020 | No outcomes of interest |
| Teixeira 2015 | Systematic review |
| Thornton 2018 | Not an RCT |
| Tremellen 2000 | Unsuitable intervention – started too early |
| Udoff 2014 | Unsuitable intervention – started too early |
| Ulug 2017 | No outcomes of interest |
| Valojerdi 2010 | Unsuitable intervention – started too early |
| Vincent 1995 | Unsuitable intervention – laparoscopic pronuclear stage transfer |
| Wu 2020 | Unsuitable intervention – started too early |
| Xing 2018 | Unsuitable intervention – started too early |
| Zarei 2013 | Duplicate |
| Zhang 2014 | Not an RCT |

**Supplementary Table SIII:** Summary of risk of bias across included randomised trials evaluating interventions to optimise the reproductive outcomes at the time of embryo transfer

| Acupuncture | | | | | | | |
| --- | --- | --- | --- | --- | --- | --- | --- |
| Study | | **Randomisation** | **Selection** | **Performance** | **Attrition** | **Detection** | **Reporting** |
| Andersen | 2010 | + | + | + | + | ? | ? |
| Benson | 2006 | ? | ? | + | + | ? | + |
| Craig | 2007 | + | + | - | - | ? | - |
| Craig | 2014 | + | + | - | + | ? | + |
| Dehghani | 2020 | ? | - | - | + | ? | ? |
| Domar | 2009 | + | + | - | + | + | ? |
| Fratterelli | 2008 | + | + | - | + | ? | + |
| Madaschi | 2010 | + | ? | - | + | ? | + |
| Moy | 2011 | + | + | + | + | ? | + |
| Ng | 2011 | + | + | + | + | ? | + |
| Omodei | 2010 | ? | + | - | + | ? | + |
| Paulus | 2002 | + | + | - | + | ? | + |
| Paulus | 2003 | + | + | + | + | + | + |
| Qu | 2014 | + | ? | + | + | ? | + |
| Seto | 2017 | + | + | + | + | ? | + |
| So | 2009 | + | + | + | + | + | ? |
| So | 2010 | + | + | + | + | + | ? |
| Westergaard | 2006 | + | + | - | + | ? | ? |
| Zhong | 2017 | ? | ? | - | + | + | + |
| Zhang | 2011 | + | ? | + | - | ? | + |
| Air vs Fluid | | | | | | | |
| Study | | **Randomisation** | **Selection** | **Performance** | **Attrition** | **Detection** | **Reporting** |
| Krampl | 1995 | ? | ? | - | ? | ? | - |
| Madani | 2010 | + | ? | ? | + | ? | + |
| Moreno | 2004 | + | + | + | ? | + | + |
| Antibiotics | | | | | | | |
| Study | | **Randomisation** | **Selection** | **Performance** | **Attrition** | **Detection** | **Reporting** |
| Brook | 2006 | + | + | + | ? | + | + |
| Peikrishvili | 2004 | + | ? | - | ? | ? | ? |
| Atosiban | | | | | | | |
| Study | | **Randomisation** | **Selection** | **Performance** | **Attrition** | **Detection** | **Reporting** |
| Ahn | 2009 | ? | ? | - | + | ? | + |
| Bosch | 2019 | - | ? | + | + | ? | + |
| He | 2016 | + | + | ? | + | + | + |
| Hebisha | 2016 | ? | ? | ? | + | + | ? |
| Moraloglu | 2010 | - | - | + | + | + | ? |
| Ng | 2014 | + | + | + | + | + | + |
| Song | 2013 | ? | ? | ? | ? | ? | ? |
| Tournaye | 2017 | ? | ? | + | + | ? | + |
| Yuan | 2019 | + | ? | + | + | ? | + |
| Bed Rest | | | | | | | |
| Study | | **Randomisation** | **Selection** | **Performance** | **Attrition** | **Detection** | **Reporting** |
| Amarin | 2004 | + | + | - | + | + | - |
| Botta and Grudzinskas | 2004 | ? | ? | - | + | + | - |
| Gaikwad | 2013 | + | + | + | + | + | - |
| Malhotra | 2019 | ? | ? | - | + | ? | - |
| Purcell | 2007 | + | + | - | + | + | - |
| Rezabek | 2001 | ? | ? | - | + | + | - |
| Catheter withdrawal | | | | | | | |
| Study | | **Randomisation** | **Selection** | **Performance** | **Attrition** | **Detection** | **Reporting** |
| Arvas | 2014 | + | - | - | + | ? | + |
| Devranoglu | 2016 | + | - | ? | - | ? | + |
| Martinez | 2001 | + | ? | ? | + | ? | ? |
| Cervical Mucus Removal | | | | | | | |
| Study | | **Randomisation** | **Selection** | **Performance** | **Attrition** | **Detection** | **Reporting** |
| Berkkanoglu | 2006 | + | ? | - | + | + | + |
| Glass | 2000 | ? | ? | ? | ? | ? | - |
| Moini | 2011 | + | ? | + | + | + | + |
| Ruhlman | 1999 | ? | ? | - | + | ? | - |
| Soroka | 1999 | ? | ? | - | + | + | - |
| Visschers | 2007 | + | + | + | + | + | + |
| Embryo Transfer Catheters | | | | | | | |
| Study | | **Randomisation** | **Selection** | **Performance** | **Attrition** | **Detection** | **Reporting** |
| Al Shawaf | 1993 | + | ? | ? | ? | ? | ? |
| Allahbadia | 2010 | + | + | ? | + | + | ? |
| Almodina | 2009 | ? | ? | ? | ? | ? | ? |
| Amorocho | 1999 | ? | ? | ? | ? | ? | ? |
| Ata | 2007 | + | ? | ? | + | + | ? |
| Boone | 2001 | + | ? | ? | + | ? | - |
| Candan | 2014 | ? | ? | ? | ? | ? | ? |
| Coroleu | 2006 | + | + | ? | + | ? | ? |
| Curfs | 2001 | + | ? | ? | + | ? | ? |
| El-Sharwarby | 2008 | ? | ? | ? | + | ? | ? |
| Foutouh | 2003 | ? | ? | ? | ? | ? | - |
| Ghazzawi | 1999 | - | ? | ? | - | ? | ? |
| Grunert | 1998 | ? | ? | ? | + | ? | ? |
| Karande | 2002 | - | - | ? | + | ? | ? |
| Lavery | 2001 | ? | ? | ? | ? | ? | ? |
| Levi-Setti | 2002 | ? | ? | ? | ? | ? | ? |
| Mayer | 1999 | - | ? | ? | - | ? | ? |
| McDonald | 2002 | + | + | ? | + | ? | ? |
| McIlveen | 2005 | + | + | ? | + | ? | ? |
| Meriano | 2000 | + | + | ? | + | ? | ? |
| Mortimer | 2002 | ? | ? | ? | ? | ? | - |
| Ocal | 2003 | ? | ? | ? | ? | ? | - |
| Perin | 1999 | ? | ? | ? | ? | ? | ? |
| Rhodes | 2007 | ? | ? | ? | ? | ? | - |
| Ruhlmann | 2015 | + | ? | ? | - | ? | ? |
| Saldeen | 2008 | + | + | ? | + | ? | ? |
| Talwar | 2011 | - | ? | ? | + | ? | ? |
| Van Weering | 2002 | + | + | ? | + | ? | ? |
| Wisanto | 1989 | + | ? | ? | ? | ? | ? |
| Yao | 2009 | + | + | ? | + | ? | ? |
| Full Bladder | | | | | | | |
| Study | | **Randomisation** | **Selection** | **Performance** | **Attrition** | **Detection** | **Reporting** |
| Lerwin | 1997 | - | - | - | ? | - | + |
| Lorusso | 2005 | + | ? | - | ? | + | ? |
| Mitchell | 1989 | + | ? | + | + | ? | ? |
| G-CSF | | | | | | | |
| Study | | **Randomisation** | **Selection** | **Performance** | **Attrition** | **Detection** | **Reporting** |
| Arefi | 2018 | + | ? | - | - | + | + |
| Obidniak | 2016 | - | - | ? | ? | - | + |
| Singh | 2015 | + | ? | ? | + | ? | + |
| Singh | 2018 | + | - | ? | ? | ? | ? |
| hCG | | | | | | | |
| Study | | **Randomisation** | **Selection** | **Performance** | **Attrition** | **Detection** | **Reporting** |
| Aaleyasin | 2015 | + | + | + | + | + | + |
| Cambiaghi | 2013 | + | ? | - | ? | + | ? |
| Dehaghani Firouzabadi | 2016 | + | ? | - | + | + | ? |
| Eskandar | 2016 | + | ? | - | ? | + | ? |
| Hafezi | 2018 | + | + | + | + | ? | + |
| Hong | 2014 | + | + | + | + | + | ? |
| Hosseini | 2016 | + | ? | - | + | + | ? |
| Laokirkkiat | 2018 | + | ? | + | - | + | + |
| Leao | 2013 | ? | ? | - | ? | + | ? |
| Mansour | 2011 | + | ? | - | ? | + | + |
| Monstajeram | 2017 | + | ? | + | ? | + | ? |
| Santibanez | 2014 | + | ? | - | + | + | ? |
| Singh | 2014 | + | ? | - | + | + | + |
| Wang | 2019 | + | ? | + | - | ? | ? |
| Wirleitner | 2015a | + | ? | - | ? | + | + |
| Wirleitner | 2015b | ? | ? | + | + | + | + |
| Zarei | 2014 | + | ? | + | - | + | ? |
| Hyaluronic Acid | | | | | | | |
| Study | | **Randomisation** | **Selection** | **Performance** | **Attrition** | **Detection** | **Reporting** |
| Balaban | 2004 | + | + | + | + | + | - |
| Dittmann-Muller | 2009 | + | ? | + | + | + | + |
| Fancsovits | 2011 | + | ? | + | ? | + | + |
| Fancsovits | 2015 | + | ? | + | + | + | + |
| Fasano | 2016 | ? | ? | ? | ? | ? | ? |
| Feichtinger | 1992 | - | ? | - | + | ? | ? |
| Friedler | 2005 | ? | ? | ? | + | ? | + |
| Friedler | 2007 | + | + | + | + | + | + |
| Kandari | 2019 | + | + | ? | + | ? | + |
| Korosec | 2007 | + | - | + | - | + | + |
| Mahani | 2007 | ? | ? | - | + | + | + |
| Morbeck | 2007 | + | + | + | + | + | + |
| Perez | 2019 | ? | ? | ? | + | ? | + |
| Ravhon | 2005 | ? | ? | ? | + | ? | + |
| Schoolcraft | 2002 | + | ? | ? | + | ? | + |
| Simon | 2003 | + | + | + | + | + | + |
| Tomari | 2014 | ? | ? | - | + | ? | ? |
| Urman | 2008 | + | + | + | + | + | - |
| Valojerdi | 2006 | ? | ? | + | - | ? | + |
| Walker | 2005 | ? | ? | ? | - | ? | + |
| Yakin | 2005 | ? | ? | ? | - | ? | + |
| Yung | 2019 | + | ? | + | + | + | + |
| NSAIDs | | | | | | | |
| Study | | **Randomisation** | **Selection** | **Performance** | **Attrition** | **Detection** | **Reporting** |
| Bernabeu | 2006 | + | ? | ? | - | + | ? |
| Dal Prato | 2009 | + | + | - | ? | ? | ? |
| Duvan | 2006a | + | + | ? | + | ? | ? |
| Fekih | 2013 | ? | ? | + | ? | ? | ? |
| Firouzabadi | 2007 | + | ? | + | + | + | + |
| Moon | 2004 | ? | ? | + | + | ? | ? |
| Zarei | 2020 | + | + | + | + | + | + |
| Nurse vs Doctor | | | | | | | |
| Study | | **Randomisation** | **Selection** | **Performance** | **Attrition** | **Detection** | **Reporting** |
| Bjuresten | 2003 | + | + | - | ? | ? | ? |
| Rinaldi | 2014 | + | + | - | + | + | + |
| Relaxation or Mindfulness* Benson 2006 and Fratterelli 2008 included in Acupuncture | | | | | | | |
| Study | | **Randomisation** | **Selection** | **Performance** | **Attrition** | **Detection** | **Reporting** |
| Aba | 2017 | + | ? | + | - | + | + |
| Gavrizi | 2019 | + | ? | + | + | ? | ? |
| MacLennan | 1985 | + | ? | + | ? | ? | ? |
| Murphy | 2014 | + | - | - | + | ? | + |
| Shaker | 1993 | + | - | + | ? | - | + |
| Seminal Fluid | | | | | | | |
| Study | | **Randomisation** | **Selection** | **Performance** | **Attrition** | **Detection** | **Reporting** |
| Aflatoonian | 2009 | + | + | - | ? | + | + |
| Karimian | 2010 | ? | ? | ? | ? | + | ? |
| Site of Deposition | | | | | | | |
| Study | | **Randomisation** | **Selection** | **Performance** | **Attrition** | **Detection** | **Reporting** |
| Coroleu | 2002 | + | ? | ? | + | ? | + |
| Franco | 2004 | + | ? | - | + | - | + |
| Nazari | 1993 | - | - | ? | ? | ? | ? |
| Ultrasound* Wisanto 1989 included in Embryo Transfer Catheters | | | | | | | |
| Study | | **Randomisation** | **Selection** | **Performance** | **Attrition** | **Detection** | **Reporting** |
| Abdelmassih | 2001 | ? | ? | - | + | ? | + |
| Ammar | 2013 | + | + | ? | + | ? | - |
| Amzy | 2009 | ? | ? | ? | + | ? | ? |
| Bar Hava | 2003 | ? | ? | ? | - | ? | - |
| Bodri | 2011 | + | ? | + | + | + | + |
| Chen | 2007 | + | - | ? | - | ? | - |
| Coroleu | 2000 | ? | ? | ? | ? | ? | - |
| Coroleu | 2002 | ? | ? | ? | ? | ? | ? |
| Dalal | 2014 | ? | - | - | ? | ? | - |
| Davar | 2007 | + | + | ? | + | ? | - |
| de Camargo Martins | 2004 | + | - | ? | ? | ? | + |
| Deep | 2013 | ? | - | - | ? | ? | + |
| Drakeley | 2008 | + | + | ? | ? | ? | ? |
| Eskandar | 2008 | ? | ? | ? | ? | ? | - |
| Garcia-Velasco | 2001 | + | - | ? | ? | ? | + |
| Garcia-Velasco | 2002 | + | + | ? | ? | ? | - |
| Hauzman | 2013 | ? | ? | - | + | ? | + |
| Kan | 1999 | + | - | + | ? | + | + |
| Karavani | 2017 | + | + | - | ? | - | + |
| Kosmas | 2007 | + | ? | ? | ? | ? | - |
| Li | 2005 | ? | ? | ? | ? | ? | - |
| Maldonado | 2005 | ? | ? | ? | - | ? | - |
| Marconi | 2003 | ? | ? | ? | ? | ? | - |
| Matorras | 2002 | + | ? | ? | ? | ? | + |
| Moraga-Sanchez | 2004 | + | ? | ? | - | ? | - |
| Porat | 2010 | + | + | - | + | ? | + |
| Prapas | 1995 | - | - | ? | ? | - | ? |
| Revelli | 2016 | + | + | - | + | ? | + |
| Sallam | 2002 | + | - | - | ? | ? | + |
| Saravelos | 2016 | + | + | + | + | + | ? |
| Tang | 2001 | + | ? | ? | - | ? | + |
| Weissman | 2003 | ? | ? | ? | - | ? | - |
| Miscellaneous | | | | | | | |
| Study | | **Randomisation** | **Selection** | **Performance** | **Attrition** | **Detection** | **Reporting** |
| Abu-Musa | 2008 | + | ? | - | + | ? | + |
| Amui | 2011 | - | - | ? | ? | ? | ? |
| Caanen | 2016 | + | + | - | + | + | + |
| Groutz | 1997 | ? | ? | ? | + | ? | + |
| Catorie | 2013 | + | ? | + | + | ? | + |
| Hannoun | 2009 | + | - | - | + | ? | + |
| Mansour | 2004 | ? | + | ? | - | - | + |
| Ng | 2019 | + | + | + | + | ? | + |
| Obidniak | 2017 | - | ? | ? | ? | - | - |
| Sigalos | 2017 | + | ? | ? | - | ? | ? |
| Yayla Abide | 2018 | + | + | + | + | + | + |

*?:Unclear -: Low +: High

**Supplementary Table SIV:** GRADE assessment of evidence evaluating all interventions performed at the time of embryo transfer in women undergoing assisted reproduction

|  | | | | | | | | |
| --- | --- | --- | --- | --- | --- | --- | --- | --- |
| **Certainty assessment** | | | | | | **Summary of findings** | | |
| **Participants  (studies) Follow up** | **Risk of bias** | **Inconsistency** | **Indirectness** | **Imprecision** | **Overall certainty of evidence** | **Study event rates (%)** | | **Relative effect (95% CI)** |
|  |  |  |  |  |  | **With Comparison** | **With Interventions at the time of ET** |  |
| ***Higher confidence and significant effect size:*** | | | | | | | | |
| **Hyaluronic acid vs no HA** | | | | | | | | |
| 2453 (9 RCTs) | not serious | not serious | not serious | not serious | ⨁⨁⨁⨁ HIGH | 286/1305 (21.9%) | 387/1148 (33.7%) | **RR 1.457** (1.197 to 1.773) |
| **Ultrasound guided ET vs clinical touch** | | | | | | | | |
| 7256 (24 RCTs) | not serious | not serious | not serious | serious ^a^ | ⨁⨁⨁◯ MODERATE | 986/3587 (27.5%) | 1245/3669 (33.9%) | **RR 1.265** (1.151 to 1.391) |
| **Softer vs harder Catheters** | | | | | | | | |
| 10688 (27 RCTs) | not serious | not serious | serious ^b^ | not serious | ⨁⨁⨁◯ MODERATE | 1704/5248 (32.5%) | 1969/5440 (36.2%) | **RR 1.122** (1.028 to 1.224) |
| ***Lower confidence and significant effect size:*** | | | | | | | | |
| **G-CSF vs placebo/no G-CSF** | | | | | | | | |
| 292 (4 RCTs) | serious ^c^ | not serious | not serious | serious ^a^ | ⨁⨁◯◯ LOW | 35/154 (22.7%) | 58/138 (42.0%) | **RR 1.774** (1.252 to 2.512) |
| **Atosiban vs placebo/no atosiban** | | | | | | | | |
| 1646 (7 RCTs) | not serious | serious ^d^ | not serious | serious ^a^ | ⨁⨁◯◯ LOW | 325/823 (39.5%) | 426/823 (51.8%) | **RR 1.493** (1.184 to 1.882) |
| **hCG vs placebo/no hCG** | | | | | | | | |
| 4653 (16 RCTs) | serious ^e^ | serious ^f^ | not serious | not serious | ⨁⨁◯◯ LOW | 883/2298 (38.4%) | 1052/2355 (44.7%) | **RR 1.232** (1.099 to 1.382) |
| **Intrauterine plasma infusion vs no infusion** | | | | | | | | |
| 90 (1 RCT) | serious ^c^ | not serious | not serious | very serious ^g^ | ⨁◯◯◯ VERY LOW | 11/45 (24.4%) | 24/45  (53.3%) | **RR 2.182** (1.219 to 3.904) |
| **Catheter rotation vs no catheter rotation** | | | | | | | | |
| 200 (1 RCT) | serious ^c^ | not serious | not serious | very serious ^g^ | ⨁◯◯◯ VERY LOW | 25/100 (25.0%) | 39/100 (39.0%) | **RR 1.560** (1.026 to 2.371) |
|  | | | | | | | | |
| ***High confidence and no significant effect:*** | | | | | | | | |
| **Transvaginal ultrasound vs transabdominal ultrasound** | | | | | | | | |
| 2942 (7 RCTs) | not serious | not serious | not serious | not serious | ⨁⨁⨁⨁ HIGH | 620/1457 (42.6%) | 638/1485 (43.0%) | **RR 1.004** (0.924 to 1.090) |
| ***Some positive associations with clinical pregnancy rate:*** | | | | | | | | |
| **NSAID's vs placebo/no NSAID's** | | | | | | | | |
| 1207 (7 RCTs) | not serious | not serious | serious ^h^ | not serious | ⨁⨁⨁◯ MODERATE | 166/599 (27.7%) | 222/608 (36.5%) | **RR 1.294** (0.973 to 1.721) |
| **Acupuncture vs placebo/no acupuncture** | | | | | | | | |
| 6496 (18 RCTs) | not serious | serious ^i^ | not serious | not serious | ⨁⨁⨁◯ MODERATE | 1379/3409 (40.5%) | 1379/3087 (44.7%) | **RR 1.121** (0.988 to 1.273) |
| **Air vs fluid in catheter tip** | | | | | | | | |
| 408 (3 RCTs) | serious ^c^ | serious ^j^ | not serious | serious ^a^ | ⨁◯◯◯ VERY LOW | 58/203 (28.6%) | 76/205 (37.1%) | **RR 1.361** (0.844 to 2.195) |
| **Echogenic vs standard catheter tip** | | | | | | | | |
| 188 (1 RCT) | serious ^c^ | not serious | not serious | very serious ^g^ | ⨁◯◯◯ VERY LOW | 39/92 (42.4%) | 53/96 (55.2%) | **RR 1.302** (0.966 to 1.756) |
| **Full bladder vs empty bladder** | | | | | | | | |
| 1069 (3 RCTs) | serious ^c^ | serious ^k^ | not serious | very serious ^g^ | ⨁◯◯◯ VERY LOW | 102/525 (19.4%) | 148/544 (27.2%) | **RR 1.266** (0.884 to 1.813) |
| **Pump regulated vs manual embryo transfer** | | | | | | | | |
| 599 (1 RCT) | serious ^c^ | not serious | not serious | very serious ^g^ | ⨁◯◯◯ VERY LOW | 65/294 (22.1%) | 81/305 (26.6%) | **RR 1.201** (0.904 to 1.600) |
| ***Lower confidence and no real associations with clinical pregnancy rate:*** | | | | | | | | |
| **Nifedipine vs placebo** | | | | | | | | |
| 93 (1 RCT) | not serious | not serious | not serious | very serious ^g^ | ⨁⨁◯◯ LOW | 11/47 (23.4%) | 12/46 (26.1%) | **RR 1.115** (0.548 to 2.267) |
| **Steroids (prednisone) vs placebo** | | | | | | | | |
| 90 (1 RCT) | not serious | not serious | not serious | very serious ^g^ | ⨁⨁◯◯ LOW | 14/40 (35.0%) | 19/50 (38.0%) | **RR 1.090** (0.626 to 1.880) |
| **Music vs no music** | | | | | | | | |
| 409 (3 RCTs) | not serious | not serious | not serious | very serious ^g^ | ⨁⨁◯◯ LOW | 101/209 (48.3%) | 102/200 (51.0%) | **RR 1.052** (0.866 to 1.279) |
| **3D vs 2D ultrasound** | | | | | | | | |
| 474 (1 RCT) | not serious | not serious | not serious | very serious ^g^ | ⨁⨁◯◯ LOW | 105/237 (44.3%) | 103/237 (43.5%) | **RR 0.981** (0.800 to 1.202) |
| **Nurse vs doctor** | | | | | | | | |
| 655 (2 RCTs) | not serious | not serious | not serious | very serious ^g^ | ⨁⨁◯◯ LOW | 115/327 (35.2%) | 111/328 (33.8%) | **RR 0.961** (0.778 to 1.187) |
| **Seminal fluid application vs no seminal fluid** | | | | | | | | |
| 385 (1 RCT) | serious ^c^ | not serious | not serious | very serious ^g^ | ⨁◯◯◯ VERY LOW | 23/195 (11.8%) | 27/190 (14.2%) | **RR 1.200** (0.717 to 2.030) |
| **Pressure on cervix vs no pressure** | | | | | | | | |
| 716 (2 RCTs) | serious ^c^ | not serious | not serious | very serious ^g^ | ⨁◯◯◯ VERY LOW | 172/359 (47.9%) | 221/357 (61.9%) | **RR 1.175** (0.817 to 1.690) |
| **Site of deposition: further from fundus (15-20mm) vs closer (<12mm)** | | | | | | | | |
| 2170 (3 RCTs) | serious ^c^ | serious ^l^ | very serious ^m^ | very serious ^g^ | ⨁◯◯◯ VERY LOW | 176/921 (19.1%) | 256/1249 (20.5%) | **RR 1.089** (0.828 to 1.431) |
| **Delayed vs immediate catheter withdrawal** | | | | | | | | |
| 672 (3 RCTs) | serious ^c^ | not serious | not serious | very serious ^g^ | ⨁◯◯◯ VERY LOW | 129/339 (38.1%) | 128/333 (38.4%) | **RR 1.039** (0.868 to 1.243) |
|  | | | | | | | | |
| **Hypnosis therapy vs benzodiazepine** | | | | | | | | |
| 93 (1 RCT) | not serious | not serious | serious ^n^ | very serious ^g^ | ⨁◯◯◯ VERY LOW | 15/43 (34.9%) | 18/50 (36.0%) | **RR 1.032** (0.595 to 1.790) |
| **Cervical mucus removal vs no removal** | | | | | | | | |
| 1157 (5 RCTs) | serious ^c^ | serious ^o^ | not serious | very serious ^g^ | ⨁◯◯◯ VERY LOW | 225/597 (37.7%) | 229/560 (40.9%) | **RR 1.029** (0.753 to 1.405) |
| **Powdered gloves vs un-powdered gloves** | | | | | | | | |
| 712 (1 RCT) | serious ^c^ | not serious | not serious | very serious ^g^ | ⨁◯◯◯ VERY LOW | 133/356 (37.4%) | 134/356 (37.6%) | **RR 1.010** (0.834 to 1.218) |
| **Antibiotics vs no antibiotics** | | | | | | | | |
| 625 (2 RCTs) | serious ^c^ | not serious | serious ^p^ | very serious ^g^ | ⨁◯◯◯ VERY LOW | 109/317 (34.4%) | 107/308 (34.7%) | **RR 1.008** (0.812 to 1.251) |
| **Glyceryl trinitrate vs placebo** | | | | | | | | |
| 120 (1 RCT) | serious ^c^ | not serious | not serious | very serious ^g^ | ⨁◯◯◯ VERY LOW | 19/60 (31.7%) | 18/60 (30.0%) | **RR 0.947** (0.554 to 1.620) |
| **Mindfulness vs no mindfulness** | | | | | | | | |
| 500 (2 RCTs) | serious ^c^ | not serious | not serious | very serious ^g^ | ⨁◯◯◯ VERY LOW | 121/247 (49.0%) | 113/253 (44.7%) | **RR 0.911** (0.755 to 1.098) |
|  | | | | | | | | |
| **17a-hydroxyprogesterone caproate vs no injection** | | | | | | | | |
| 125 (1 RCT) | serious ^c^ | not serious | not serious | very serious ^g^ | ⨁◯◯◯ VERY LOW | 24/62 (38.7%) | 22/63 (34.9%) | **RR 0.902** (0.569 to 1.430) |
| **Porcine relaxant vs placebo** | | | | | | | | |
| 96 (1 RCT) | serious ^c^ | not serious | not serious | very serious ^g^ | ⨁◯◯◯ VERY LOW | 10/45 (22.2%) | 10/51 (19.6%) | **RR 0.882** (0.405 to 1.924) |
| **High vs low volume of culture medium transferred** | | | | | | | | |
| 216 (1 RCT) | serious ^c^ | not serious | not serious | very serious ^g^ | ⨁◯◯◯ VERY LOW | 57/105 (54.3%) | 52/111 (46.8%) | **RR 0.863** (0.662 to 1.125) |
| **Transmyometrial vs transcervical embryo transfer** | | | | | | | | |
| 40 (1 RCT) | serious ^c^ | not serious | not serious | very serious ^g^ | ⨁◯◯◯ VERY LOW | 3/20 (15.0%) | 1/20 (5.0%) | **RR 0.333** (0.038 to 2.940) |
| ***Significant negative association with clinical pregnancy rates:*** | | | | | | | | |
| **Bed rest vs shorter/no bed rest** | | | | | | | | |
| 1180 (6 RCTs) | serious ^c^ | not serious | not serious | serious ^a^ | ⨁⨁◯◯ LOW | 217/591 (36.7%) | 185/589 (31.4%) | **RR 0.857** (0.741 to 0.991) |
| **Straight vs bent catheter tip** | | | | | | | | |
| 298 (1 RCT) | very serious ^c^ | not serious | not serious | very serious ^g^ | ⨁◯◯◯ VERY LOW | 40/146 (27.4%) | 27/152 (17.8%) | **RR 0.648** (0.421 to 0.999) |
| **Double vs single lumen catheter** | | | | | | | | |
| 66 (1 RCT) | serious ^c^ | not serious | not serious | very serious ^g^ | ⨁◯◯◯ VERY LOW | 15/32 (46.9%) | 5/34 (14.7%) | **RR 0.314** (0.129 to 0.764) |

**CI:** Confidence interval; **RR:** Risk ratio

**Explanations**

a. Wide confidence intervals

b. Significant heterogeneity in study design: lots of different catheter types compared which we believe may have impacted outcomes

c. Downgraded due to concerns about whole risk of bias

d. Downgraded due to high heterogeneity: I2 = 68.27%

e. Downgraded due to concerns about allocation concealment and lack of blinding

f. Downgraded due to high heterogeneity: I2 = 57.76%

g. Very wide confidence intervals

h. Significant heterogeneity in study design: lots of different NSAID's and length of treatment

i. Downgraded due to high heterogeneity: I2 = 75.14%

j. Downgraded due to high heterogeneity: I2 = 61.16%

k. Downgraded due to high heterogeneity: I2 = 50.68%

l. Downgraded due to high heterogeneity: I2 = 60.99%

m. Significant heterogeneity in study design, not consistent site of deposition in any of them

n. No control group

o. Downgraded due to high heterogeneity: I2 = 75.89%

p. Amoxicillin vs co-amoxiclav

**Supplementary Figure S1:** Funnel plot to evaluate the risk of publication bias in included randomised trials evaluating interventions to optimise the reproductive outcomes at the time of embryo transfer after excluding interventions evaluated in a single RCT.


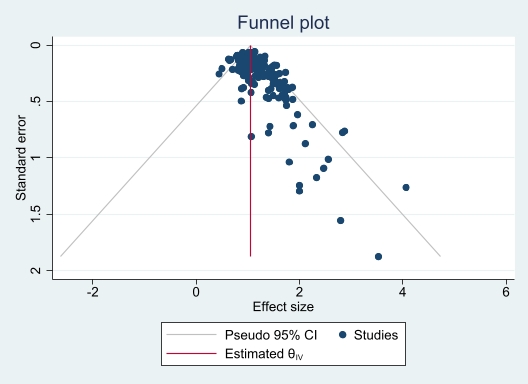


**Supplementary Figure S2:** Forest plot of the effectiveness of using anti-biotics at the time of embryo transfer on reproductive outcomes.

1. Clinical pregnancy

**Supplementary Figure S3:** Forest plot of the effectiveness of using Atosiban at the time of embryo transfer on reproductive outcomes.

1. Clinical pregnancy

**
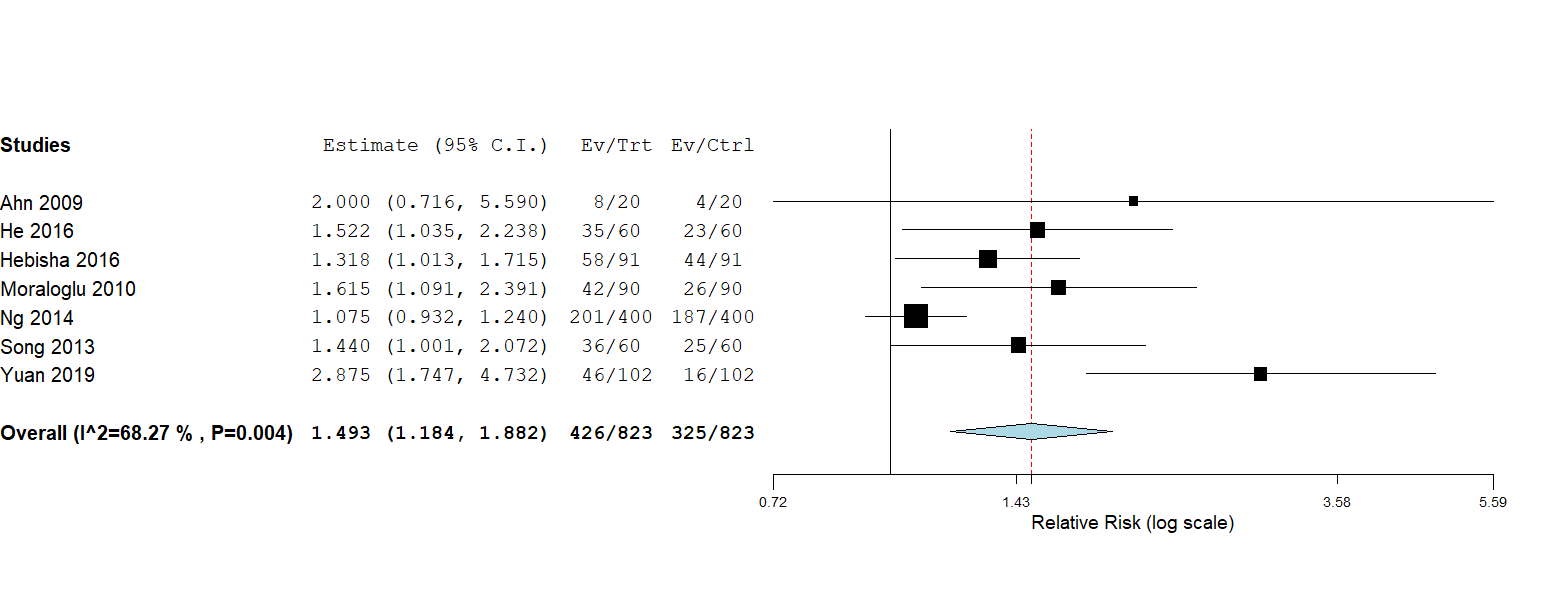
**

1. Biochemical pregnancy


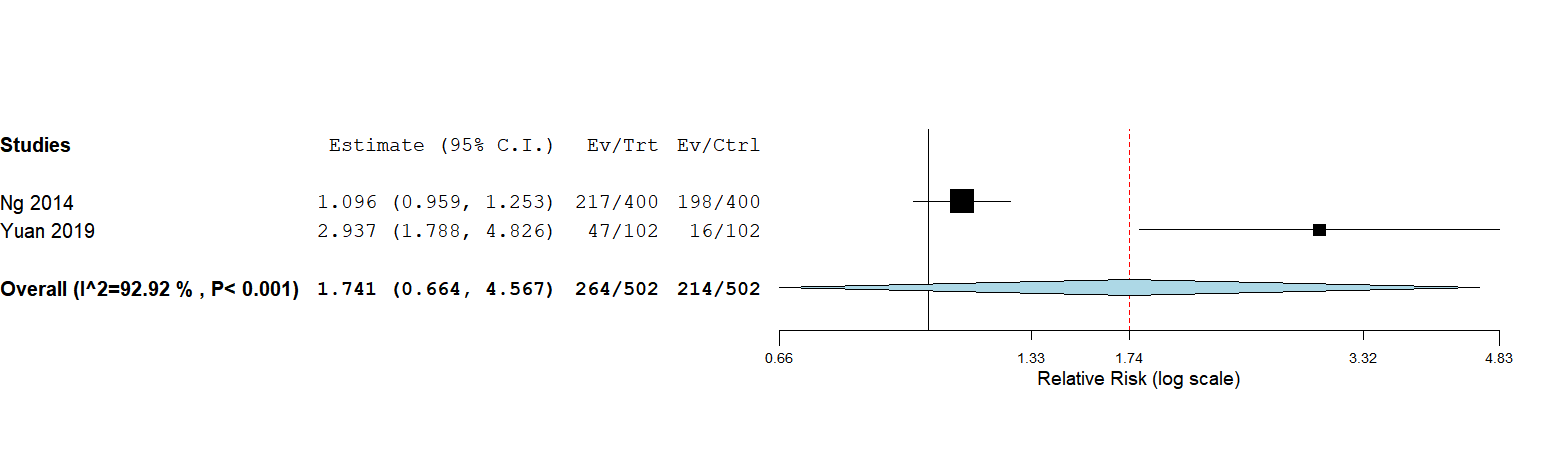


1. Ongoing pregnancy


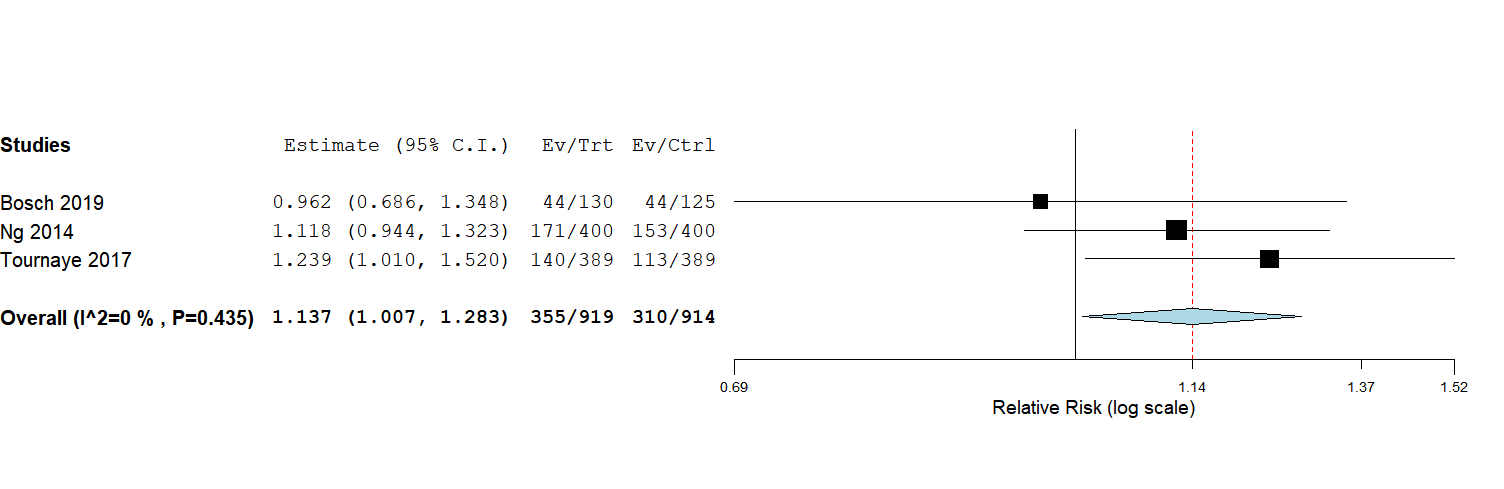


1. Miscarriage


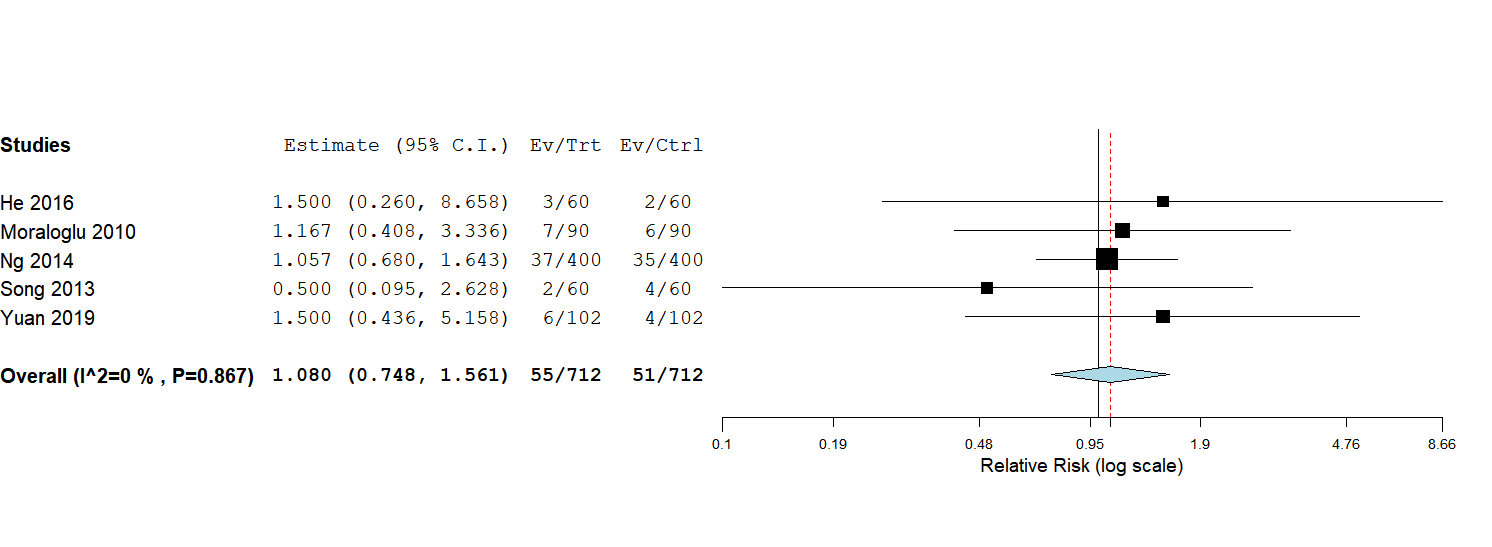


1. subgroup analysis by Atosiban dose on clinical pregnancy


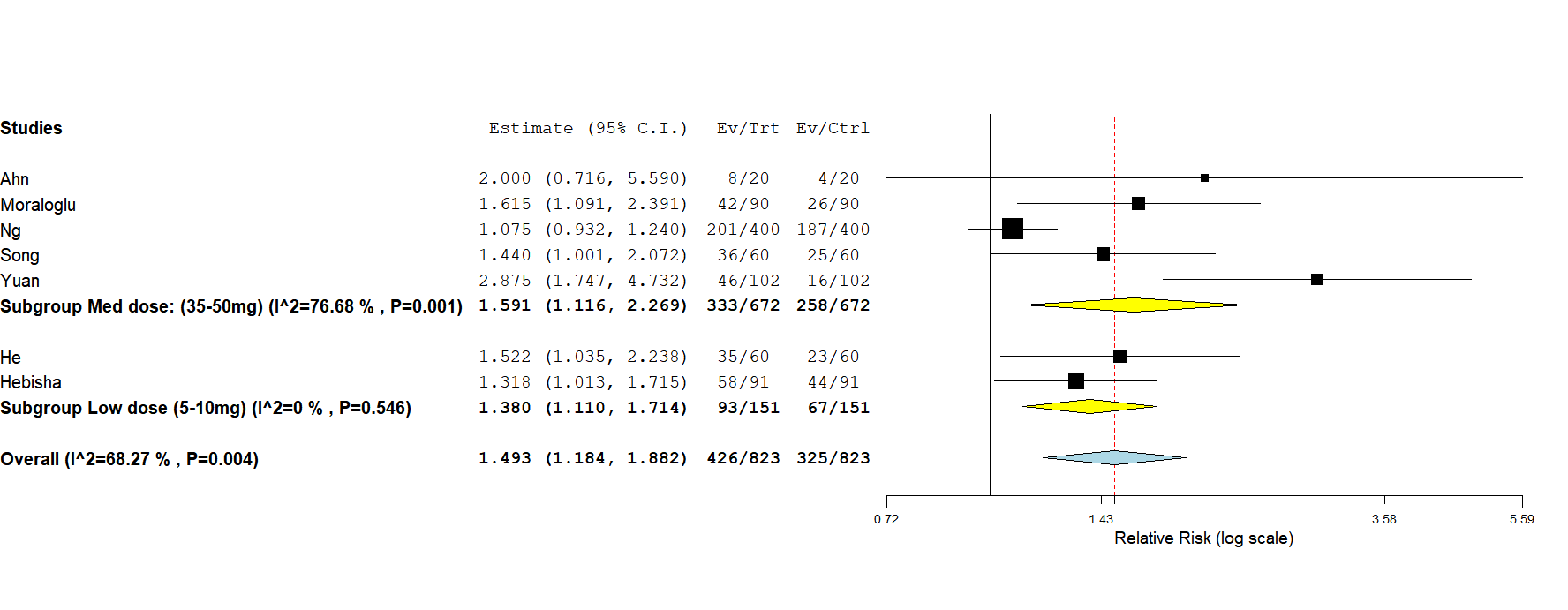


**Supplementary Figure S4:** Forest plot of the effectiveness of using NSAIDs at the time of embryo transfer on reproductive outcomes.

a: clinical pregnancy


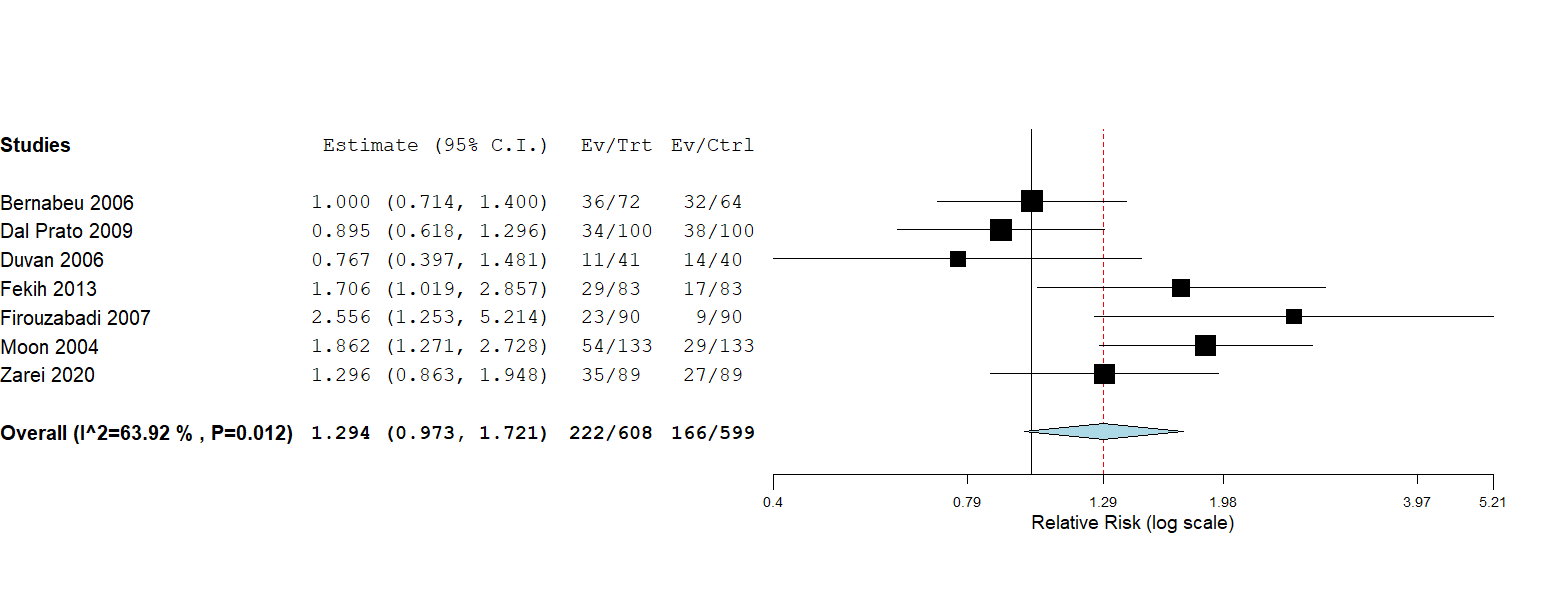


b: biochemical pregnancy


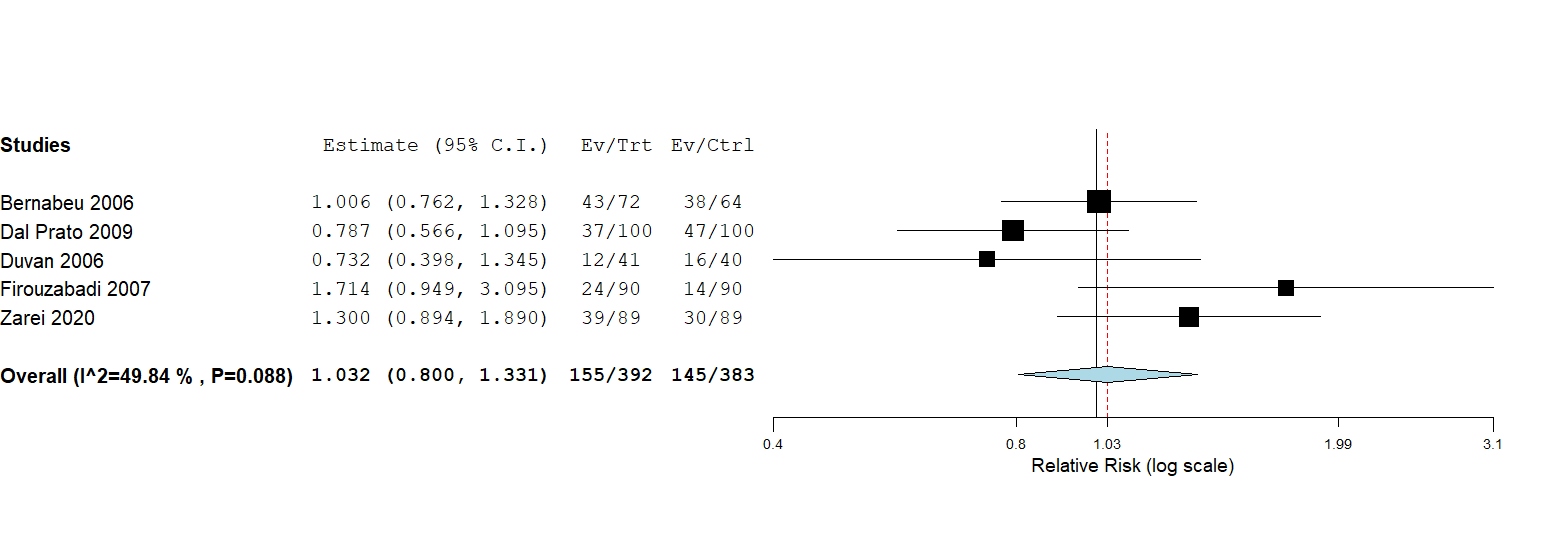


c: miscarriage


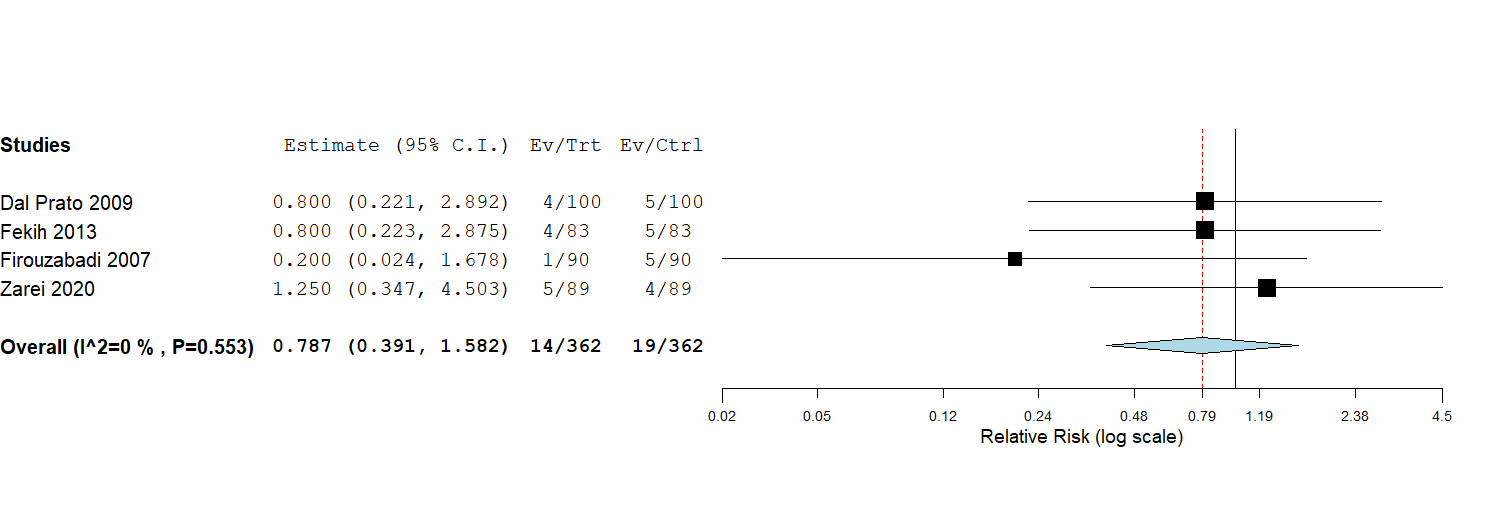


d: subgroup analysis by type of NSAID on clinical pregnancy


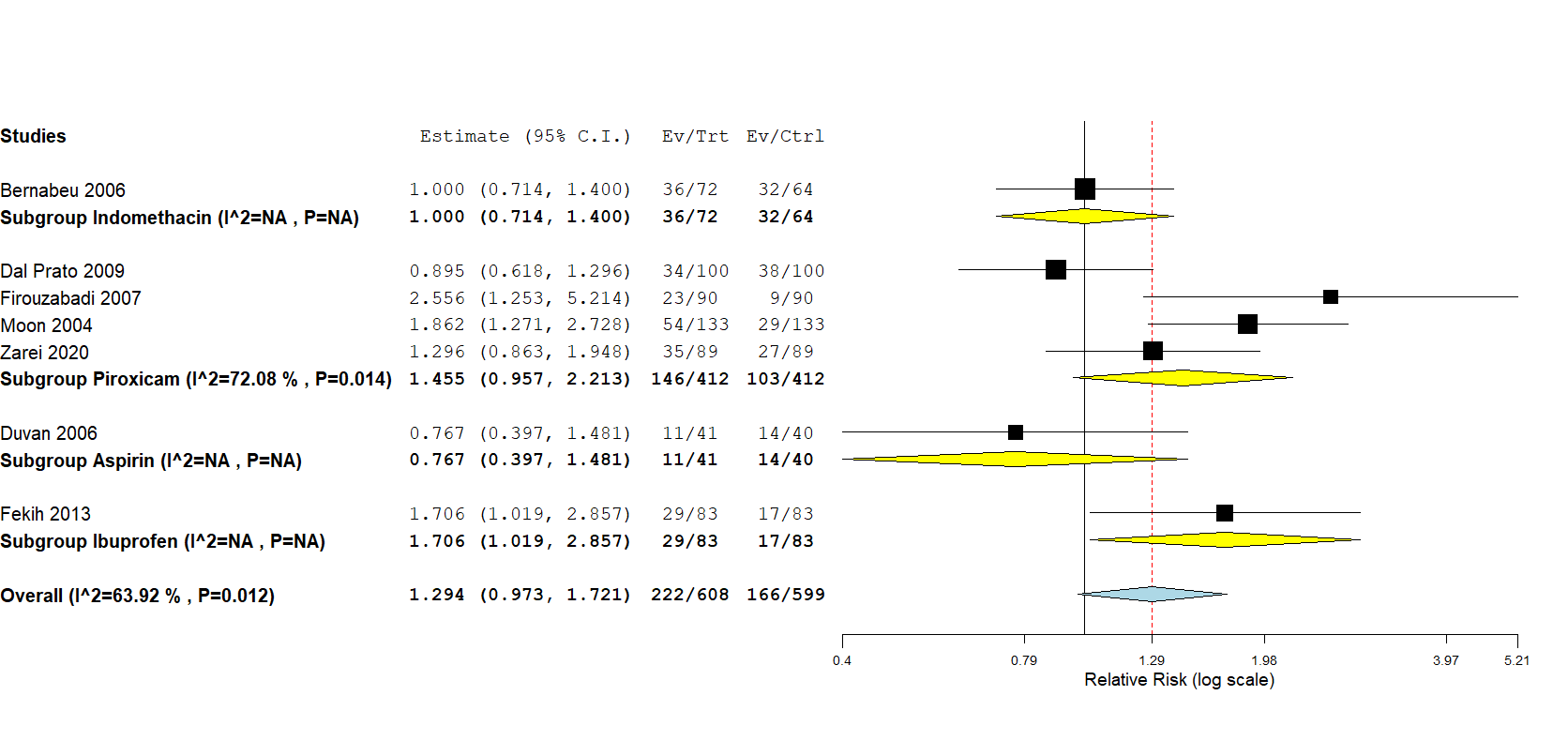


**Supplementary Figure S5:** Forest plot of the effectiveness of using hCG at the time of embryo transfer on reproductive outcomes.

a: clinical pregnancy


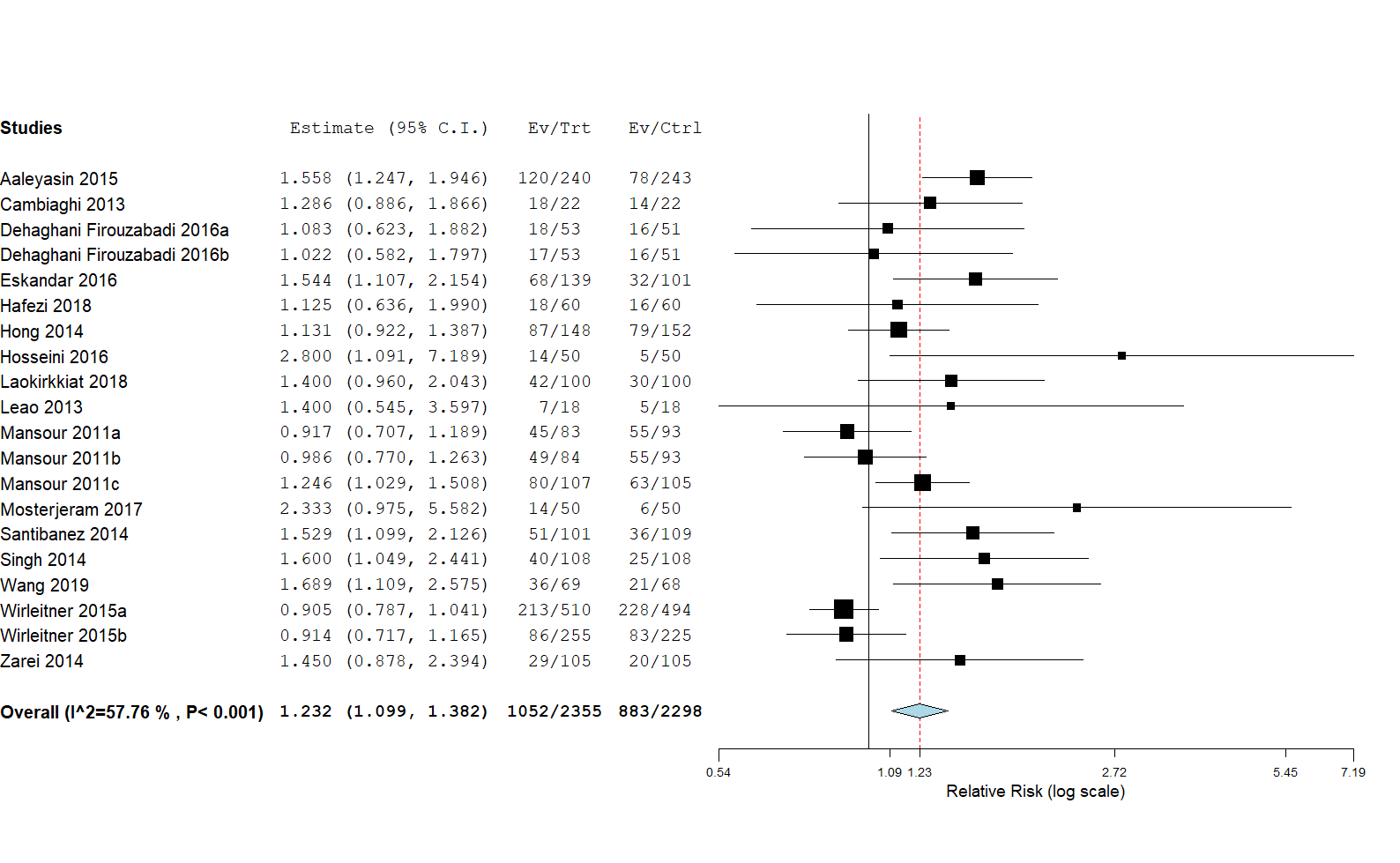
b: biochemical pregnancy


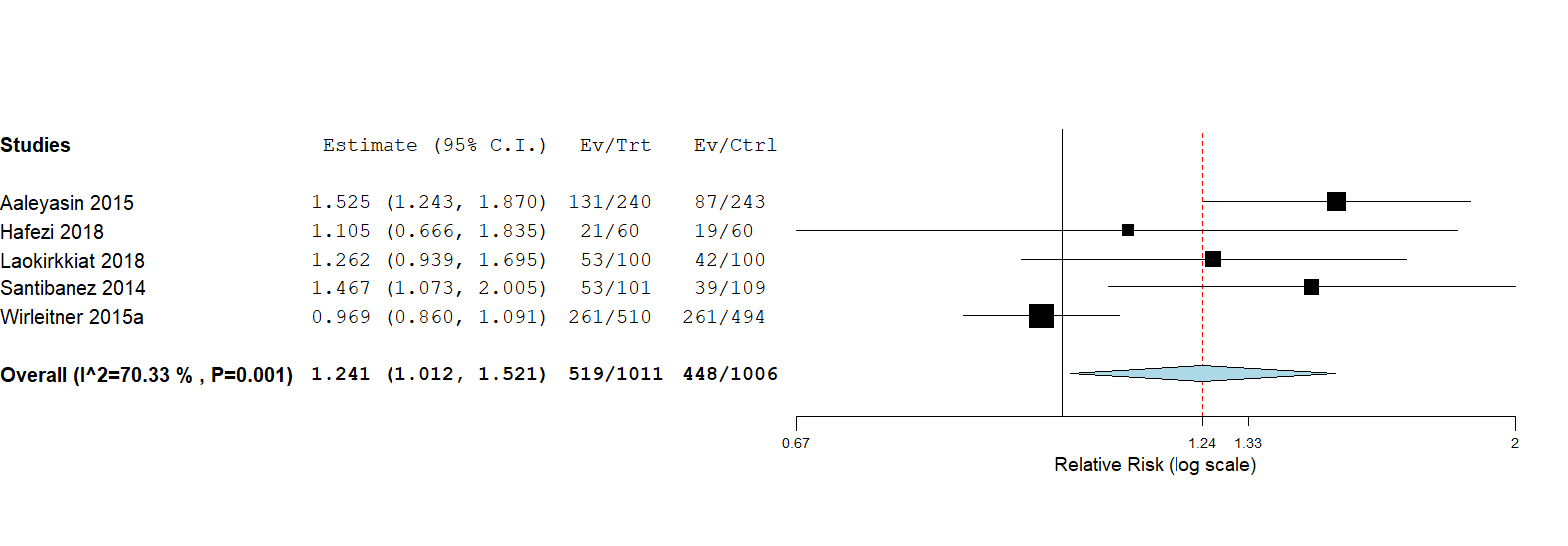


c: ongoing pregnancy


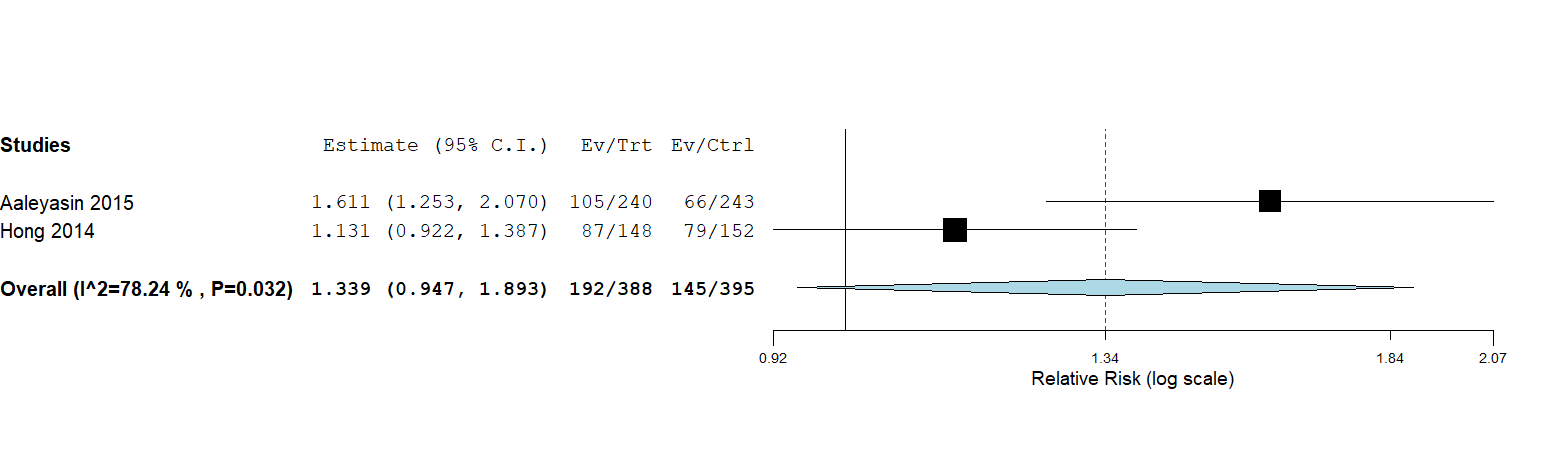


d: miscarriage


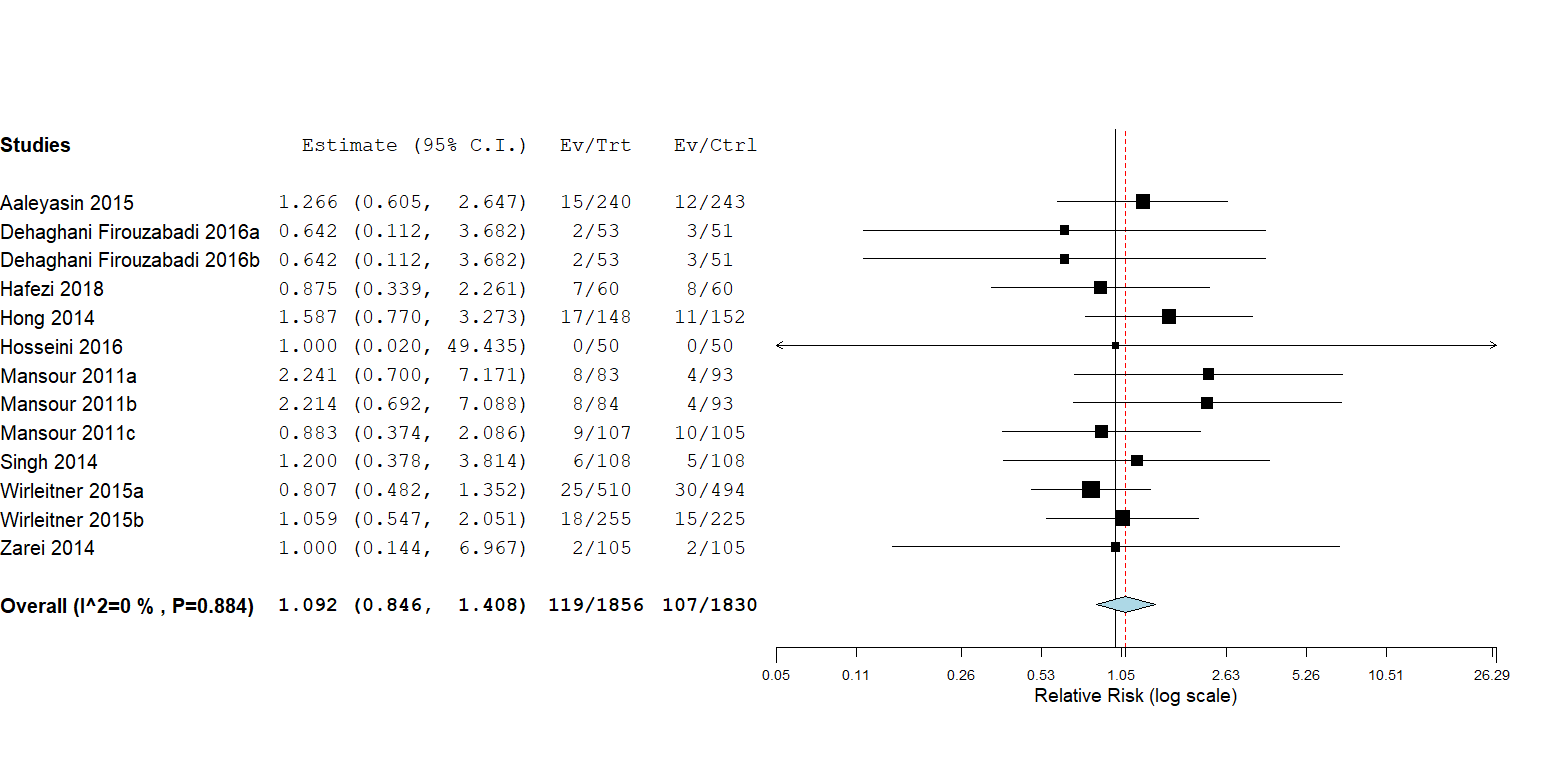


e: live birth


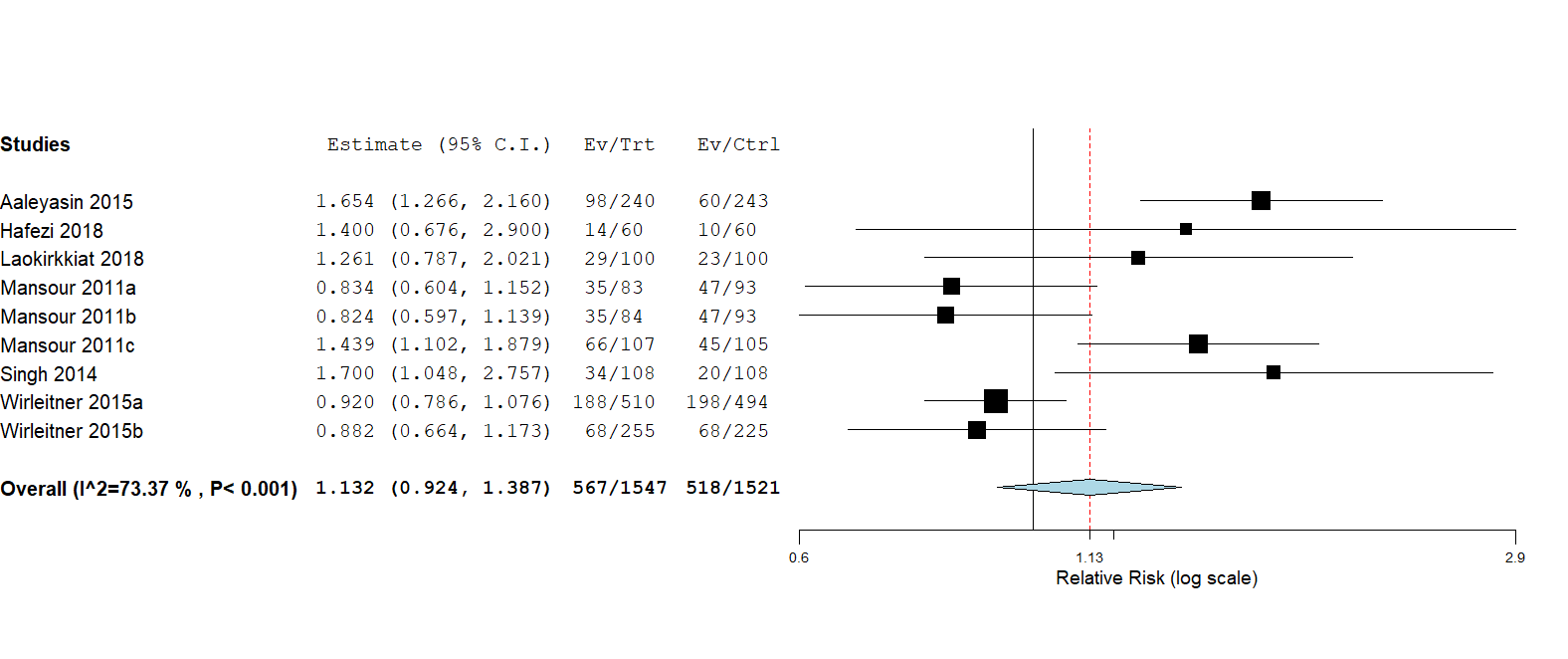


f: subgroup analysis by hCG dose on clinical pregnancy


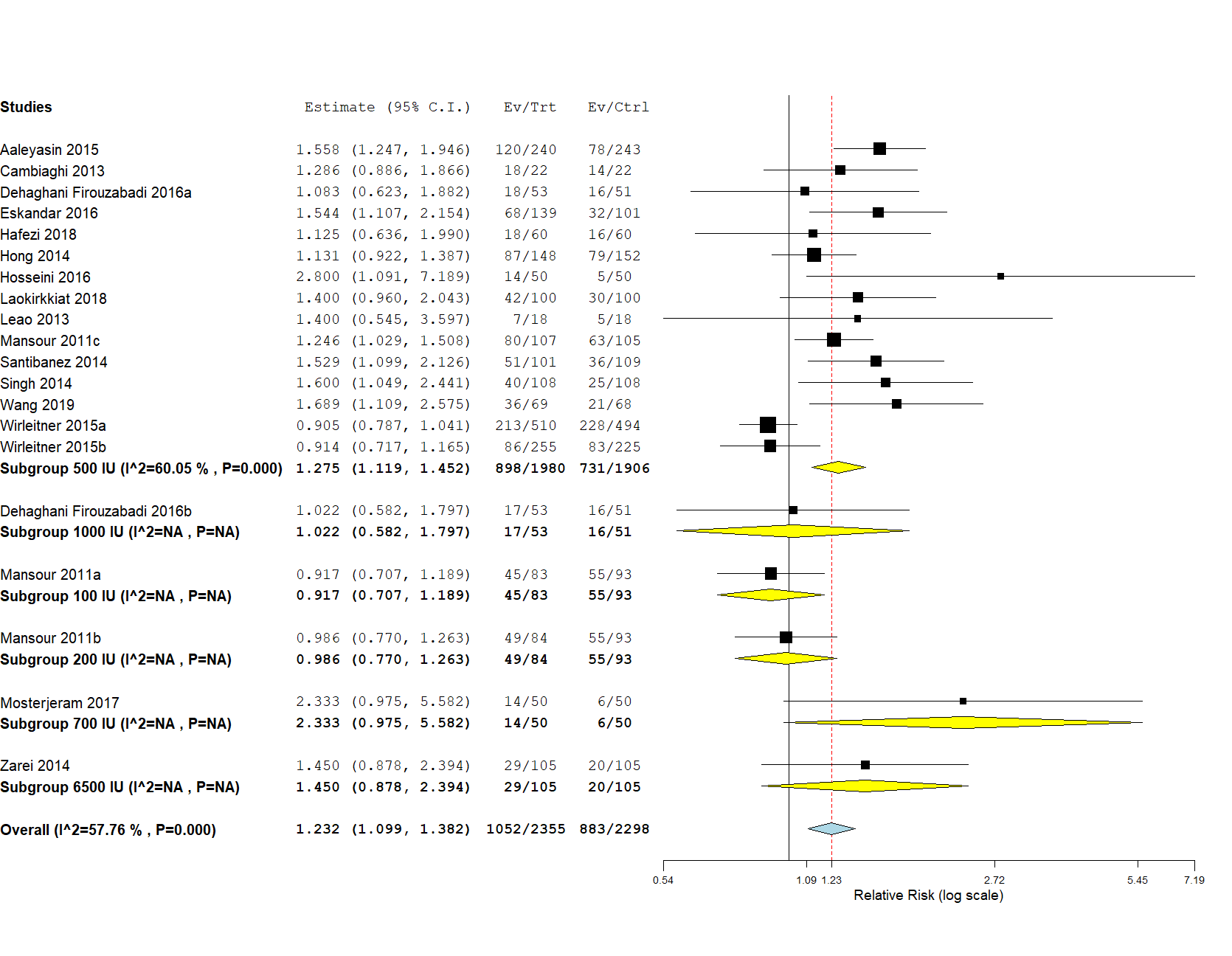


g: subgroup analysis by comparison (control injection or no treatment) on clinical pregnancy


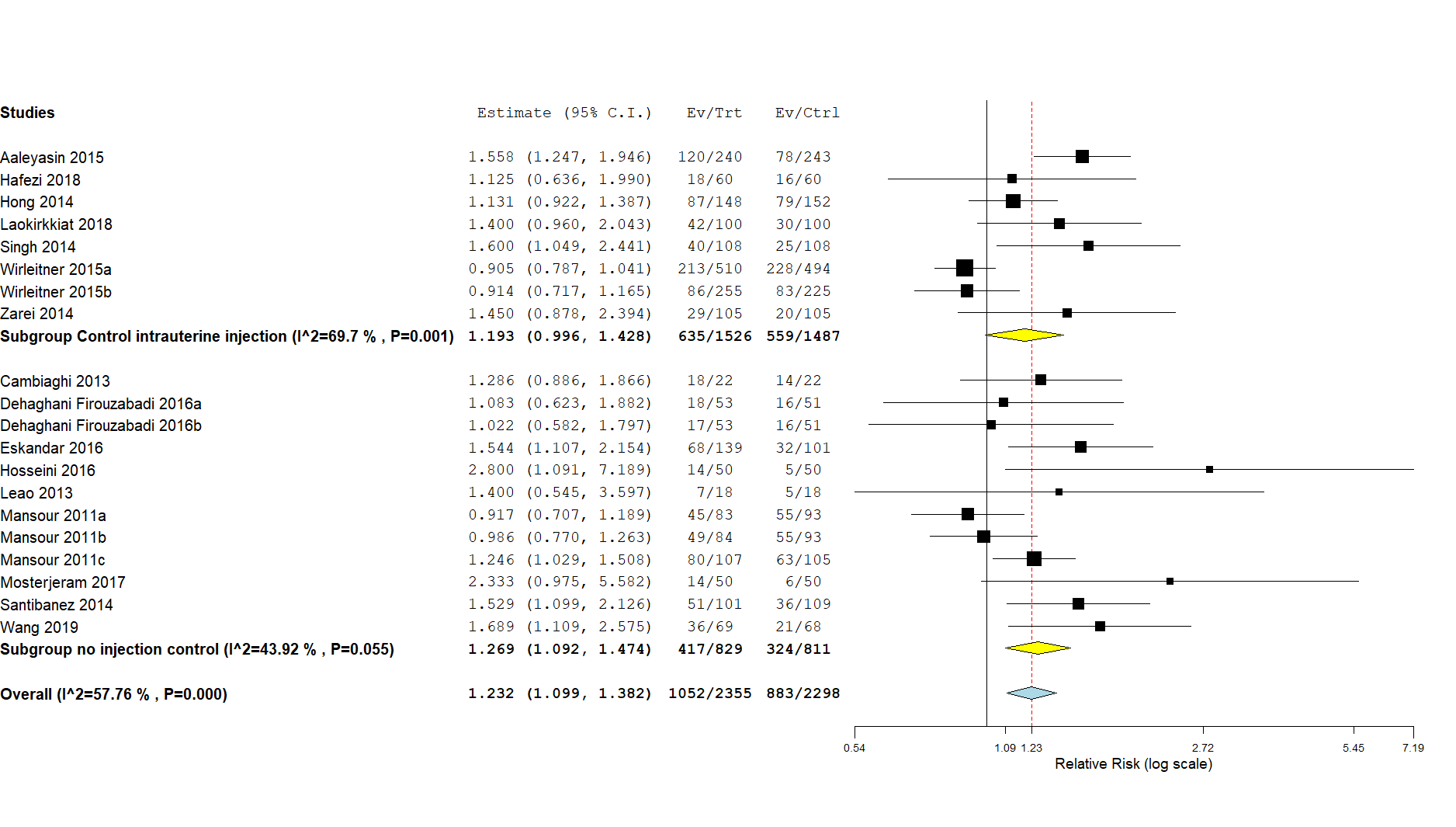


**Supplementary Figure S6:** Forest plot of the effectiveness of using hyaluronic acid at the time of embryo transfer on reproductive outcomes.

a: clinical pregnancy


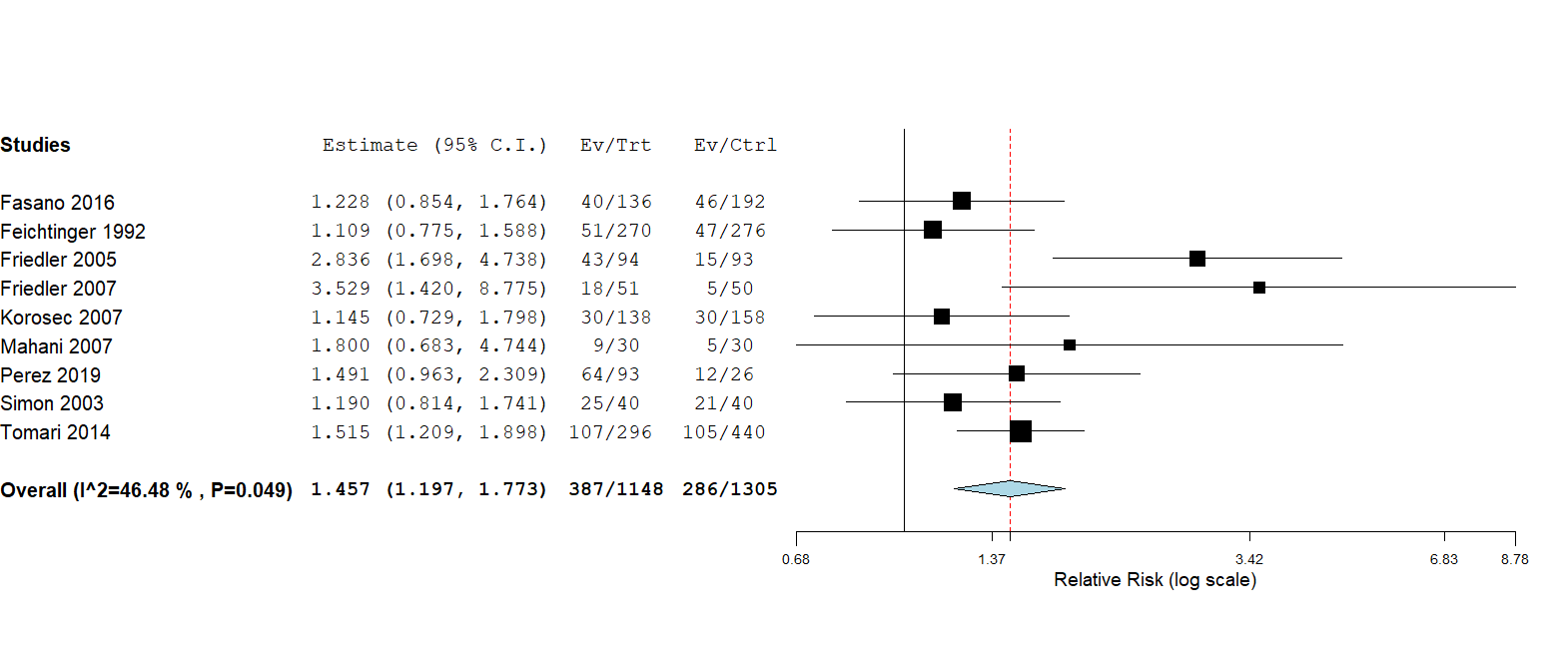


b: ongoing pregnancy


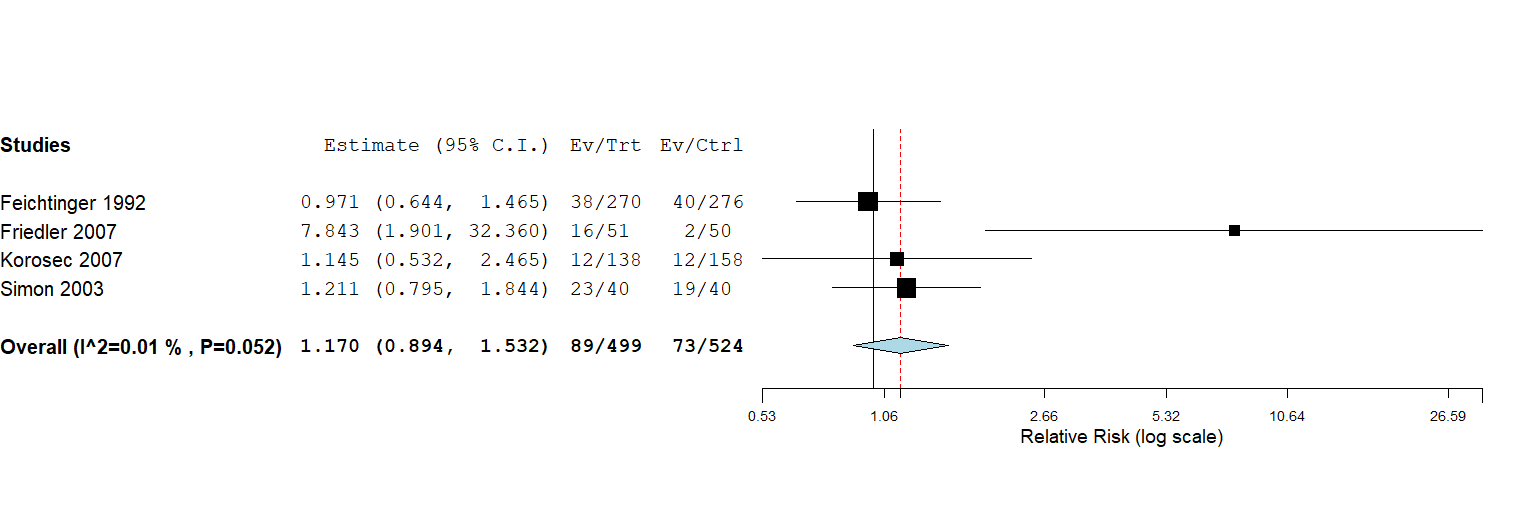


c: live birth


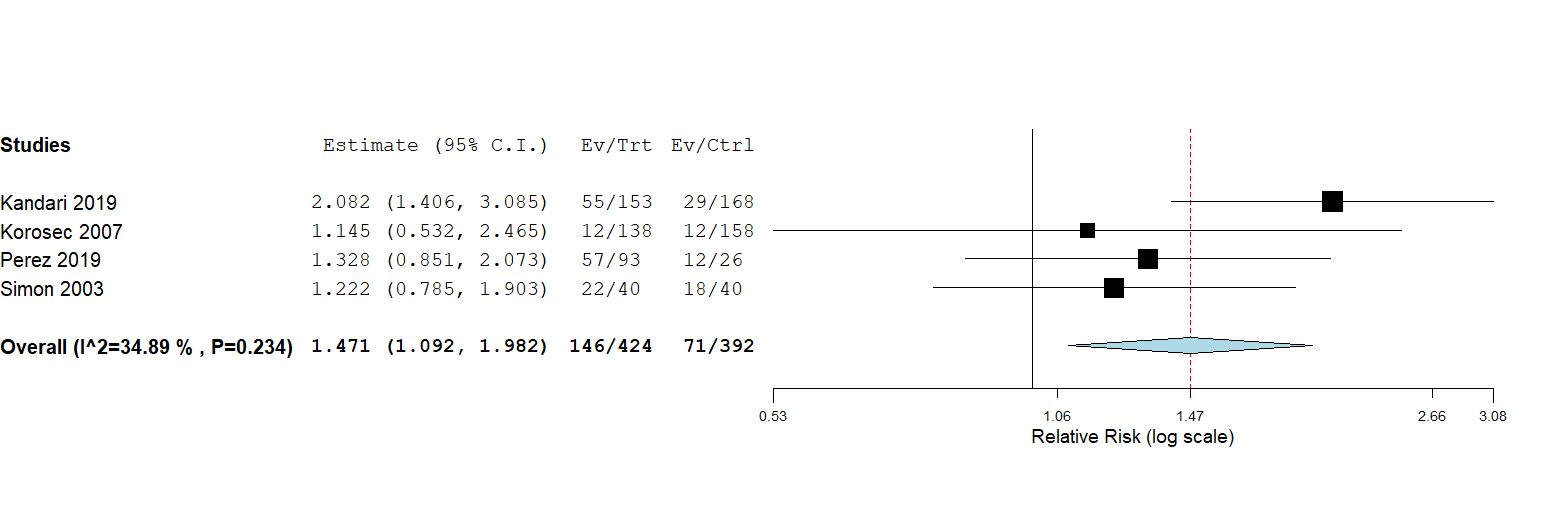


**Supplementary Figure S7:** Forest plot of the effectiveness of high vs low doses of hyaluronic acid at the time of embryo transfer on reproductive outcomes.

a: clinical pregnancy


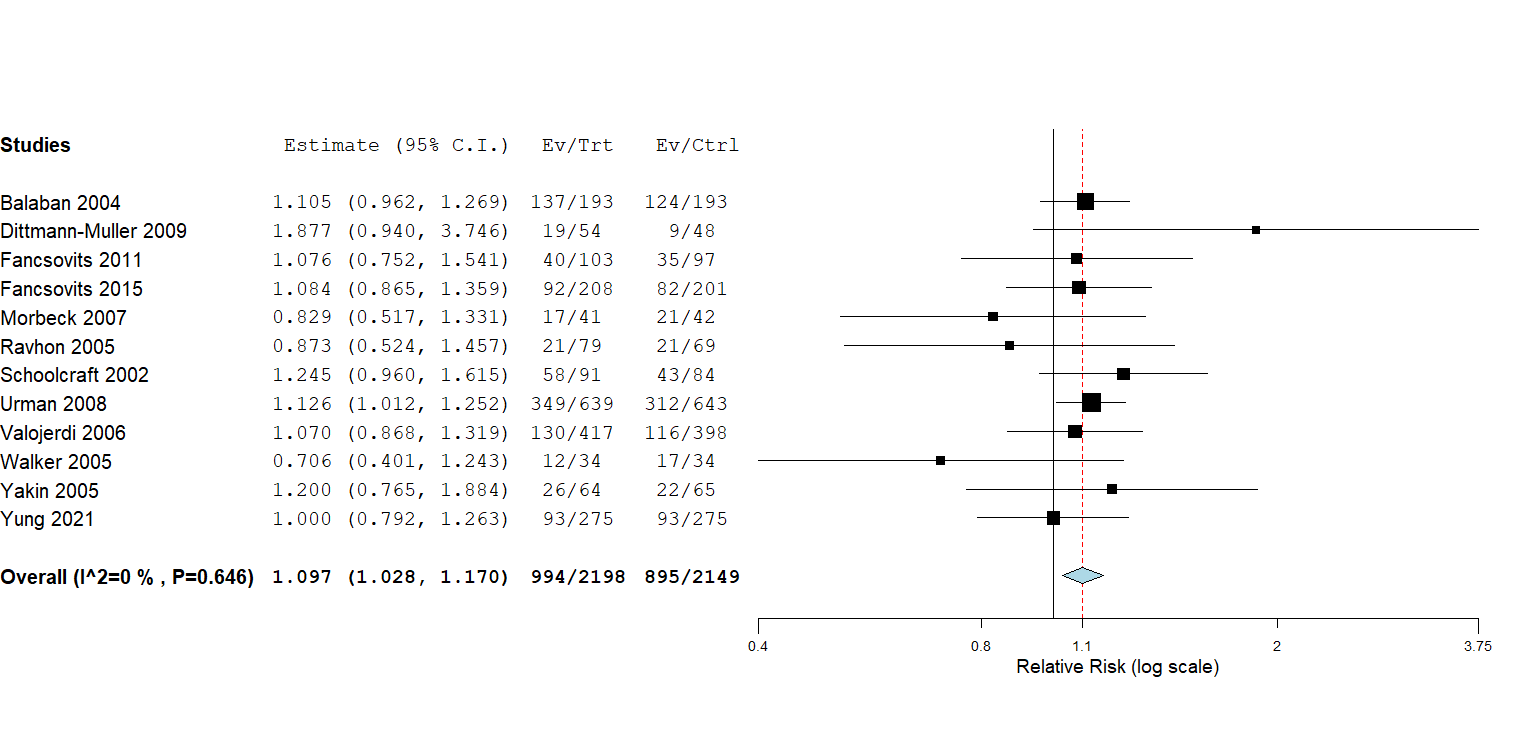


b: ongoing pregnancy


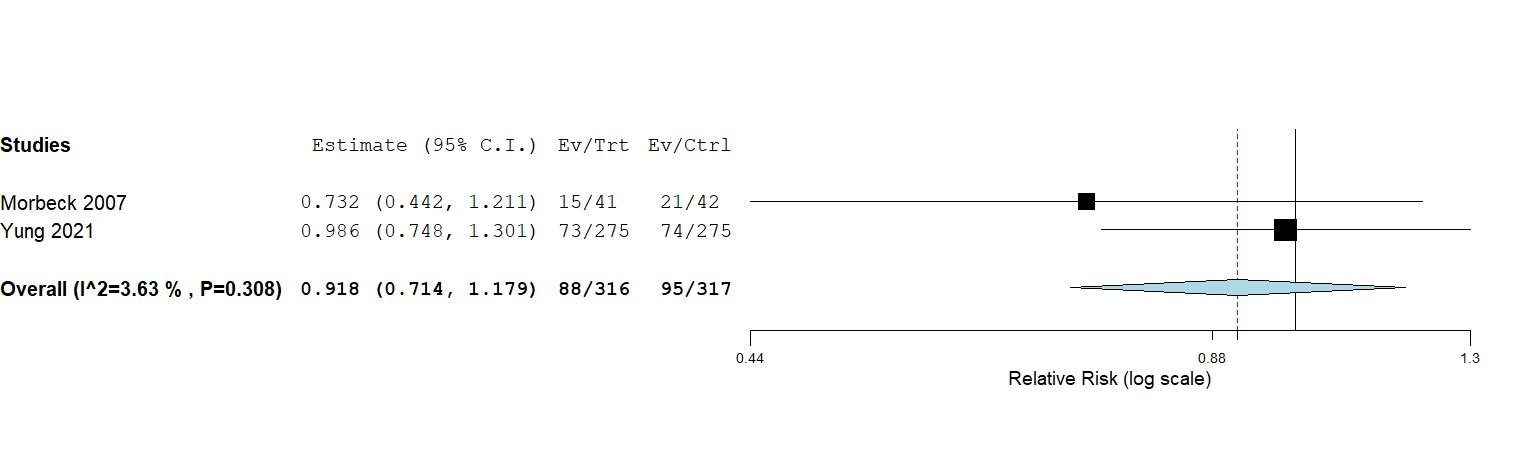


c: live birth


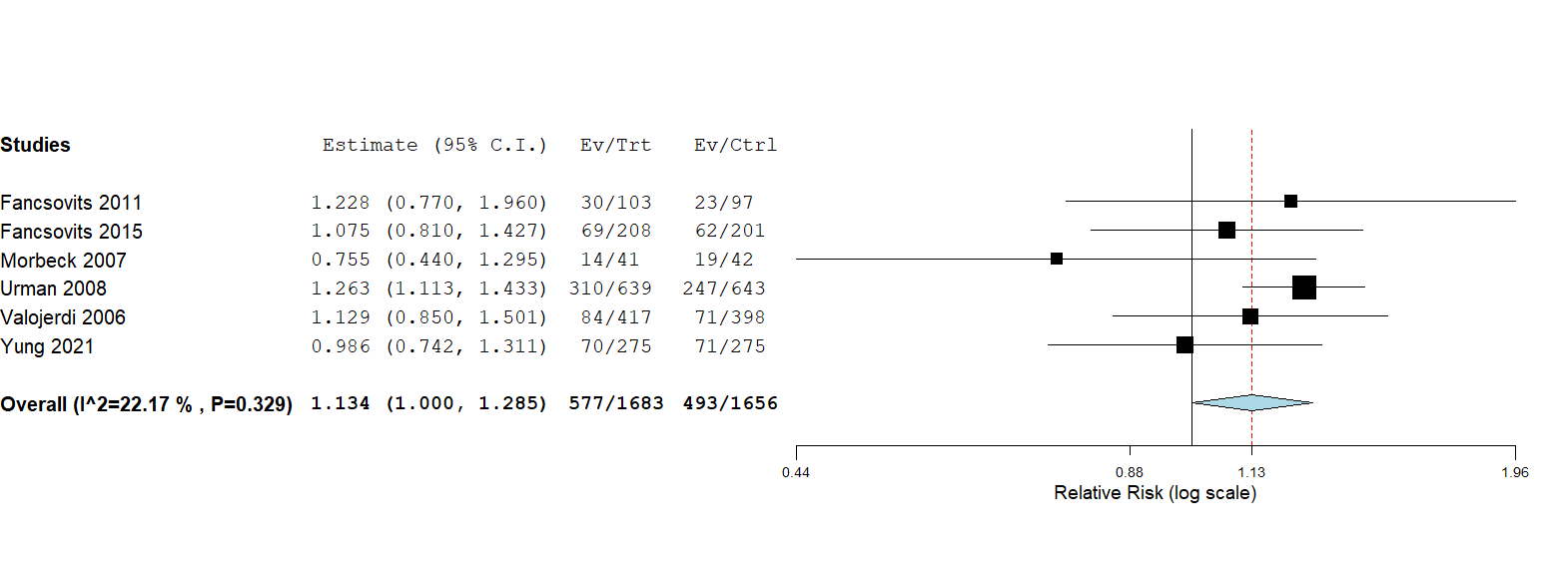


**Supplementary Figure S8:** Forest plot of the effectiveness of using G-CSF at the time of embryo transfer on clinical pregnancy


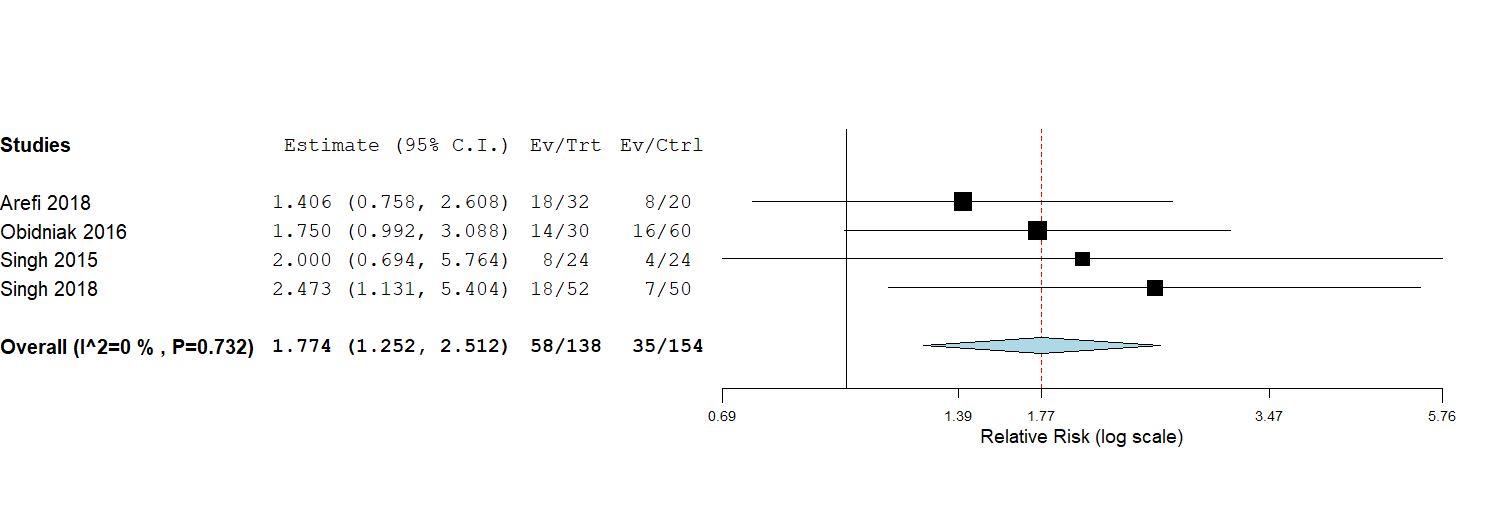


**Supplementary Figure S9:** Forest plot of the effectiveness of using seminal fluid at the time of embryo transfer on biochemical pregnancy


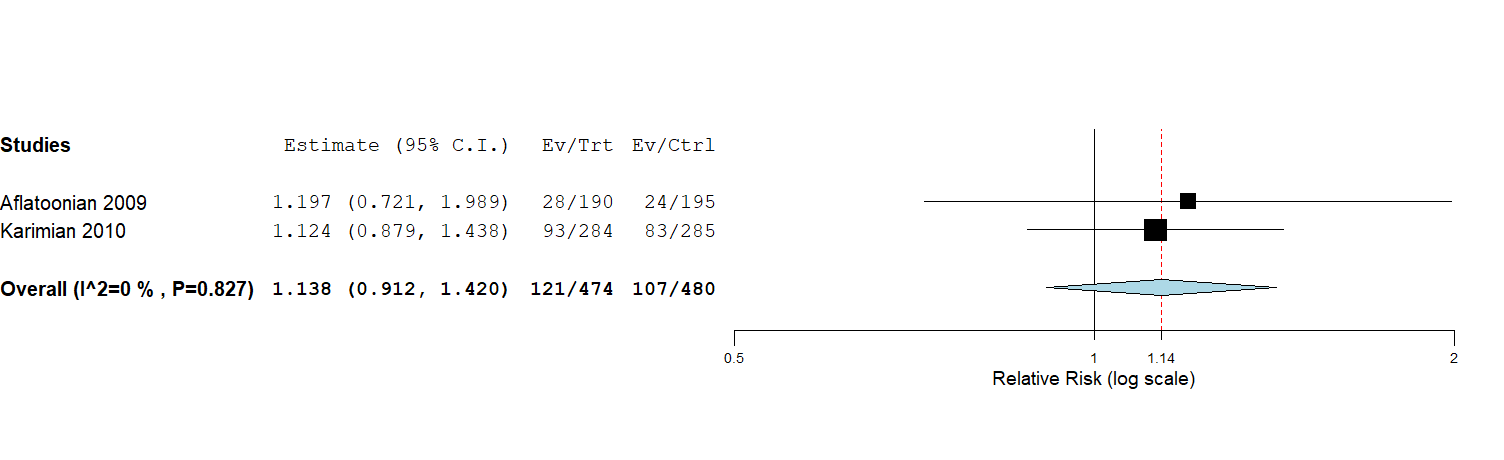


**Supplementary Figure S10:** Forest plot of the effectiveness of using ultrasound at the time of embryo transfer on reproductive outcomes.

a: clinical pregnancy


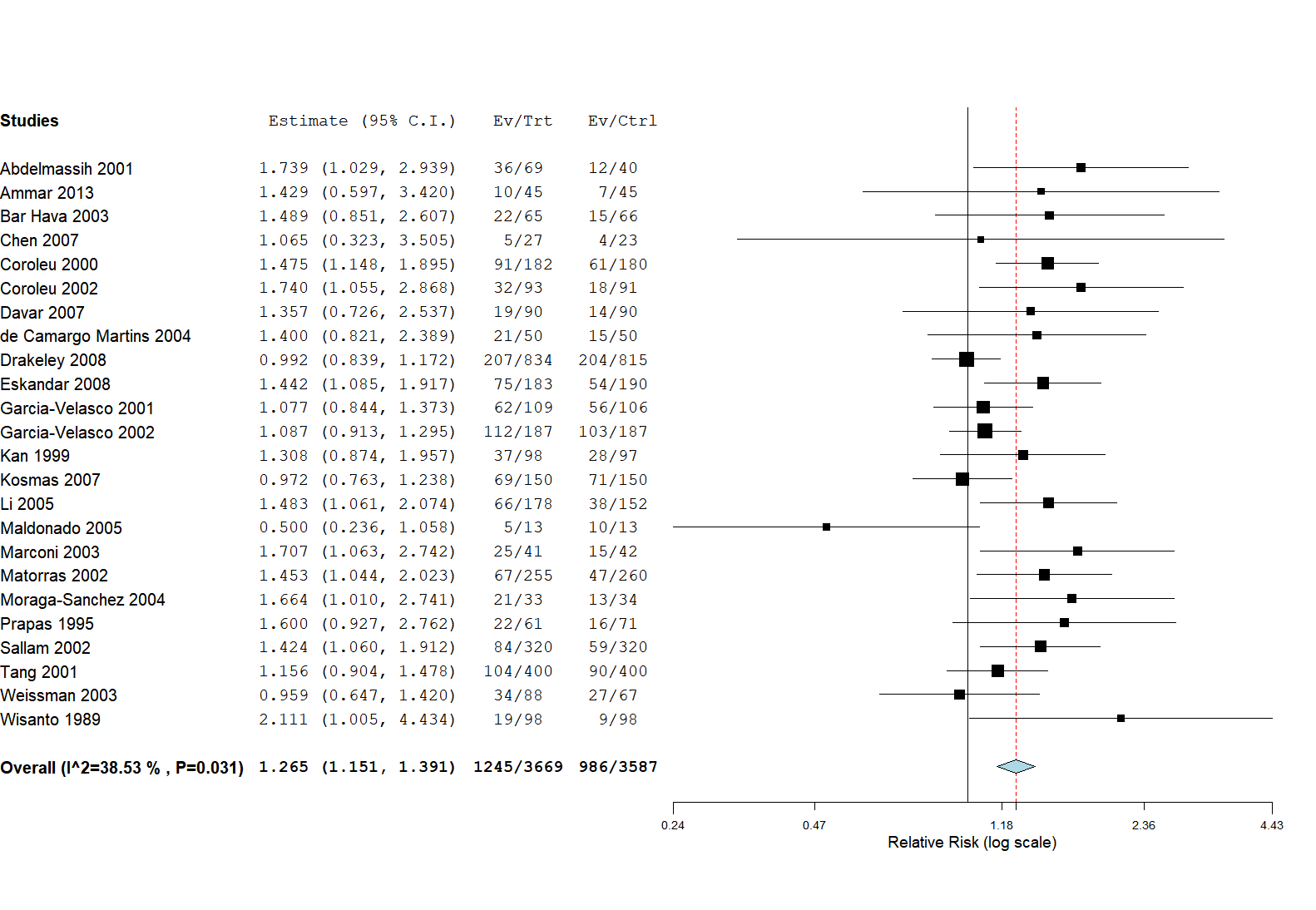


b: biochemical pregnancy


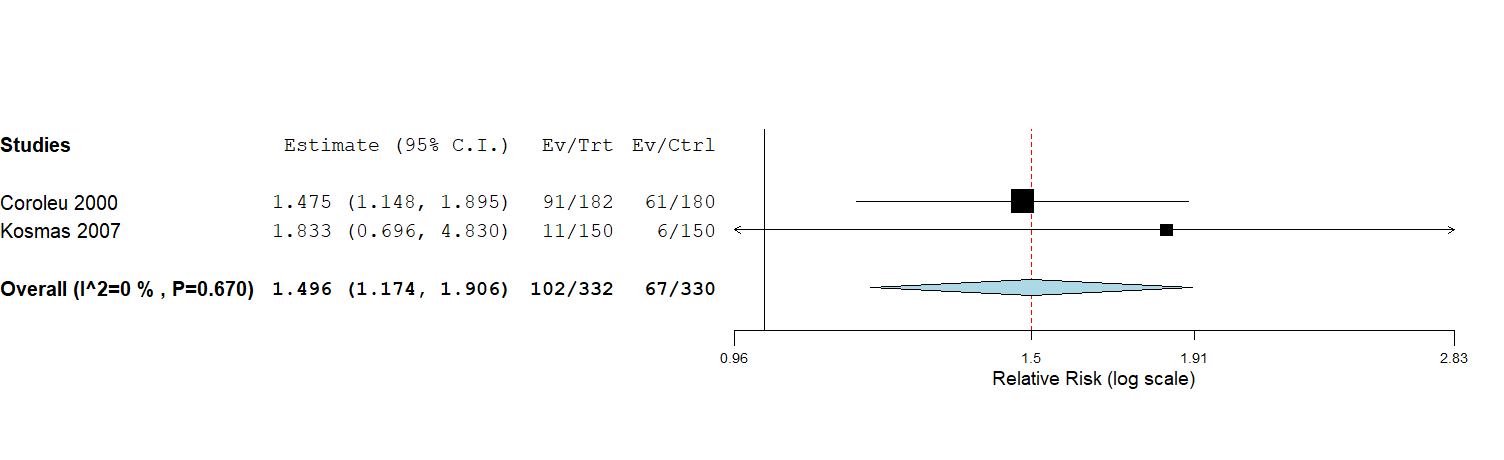


c: ongoing pregnancy


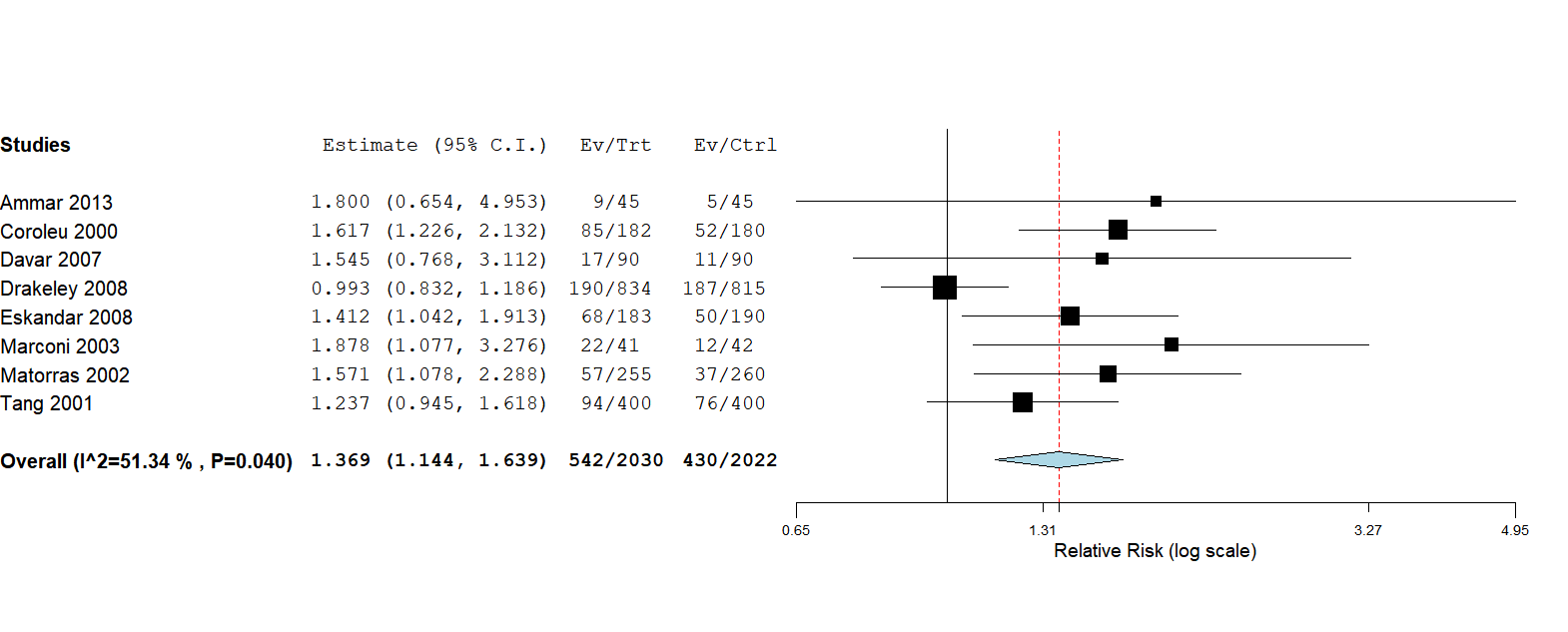


d: miscarriage


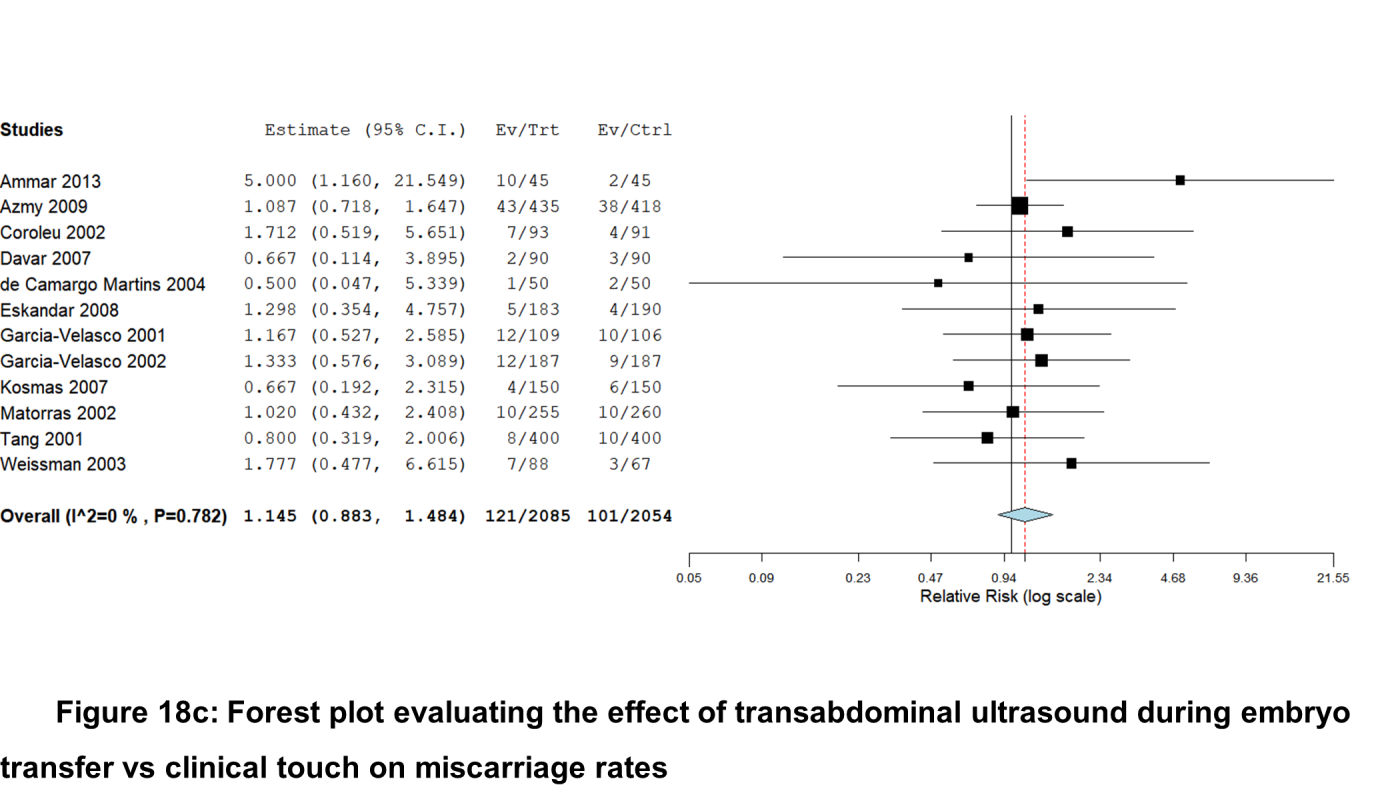


e: live birth


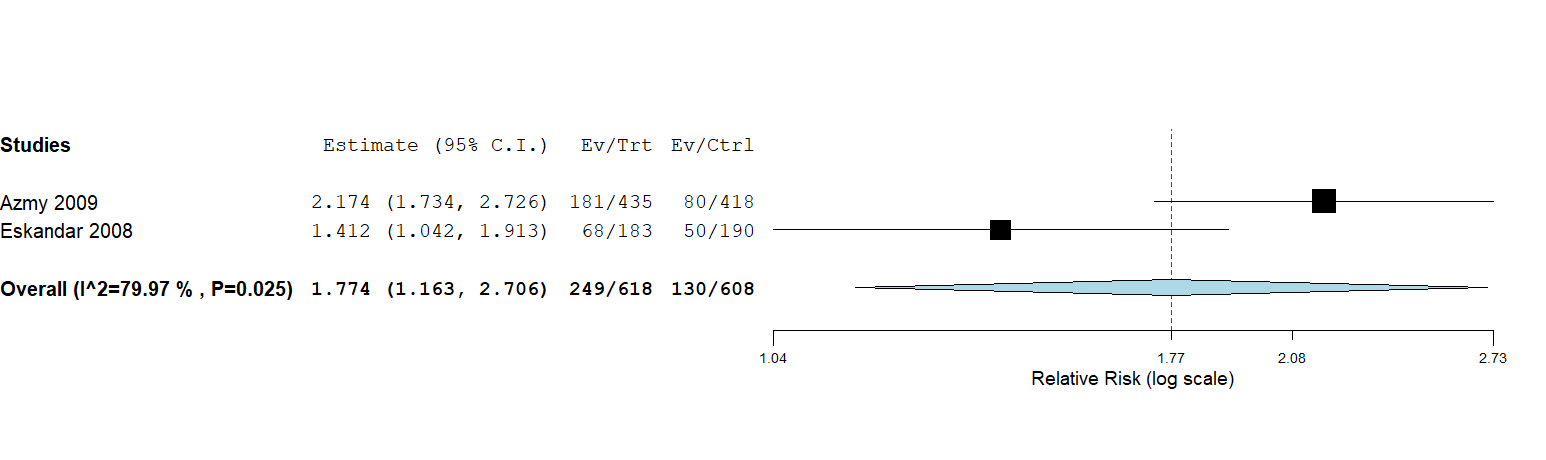


f: subgroup analysis by ultrasound route (TA vs TV) on clinical pregnancy


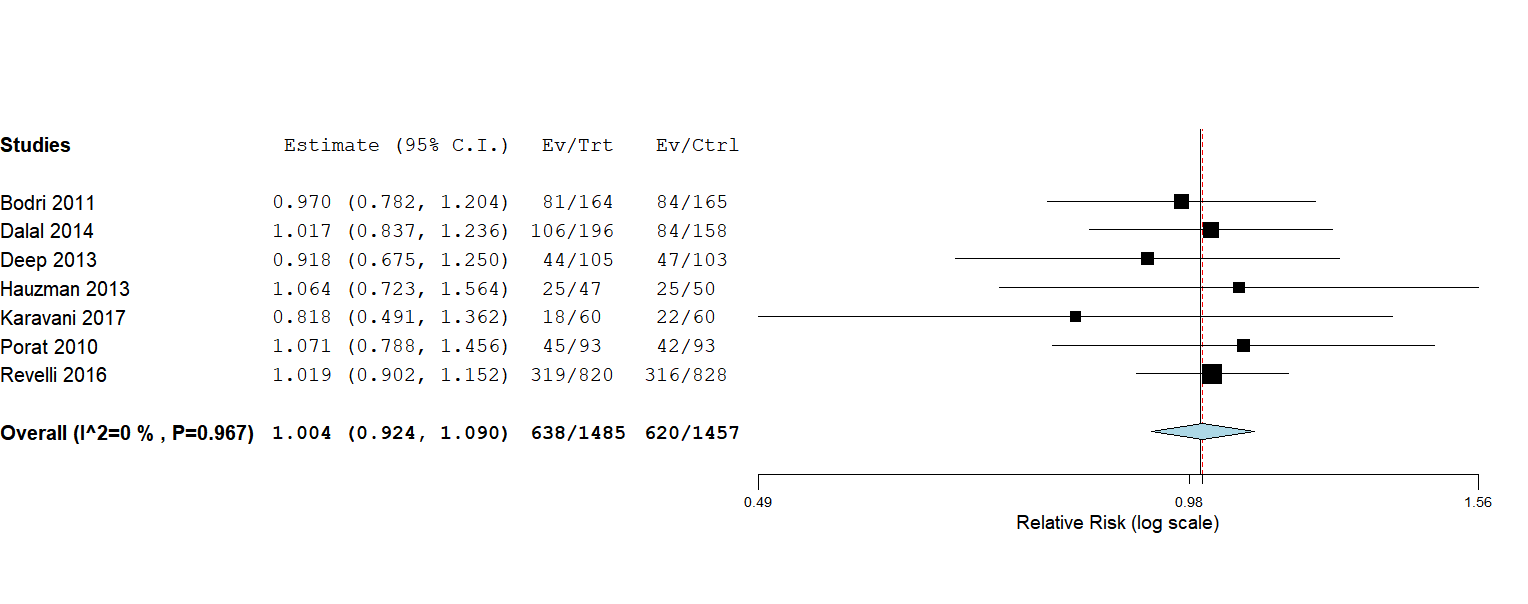


g: subgroup analysis by ultrasound route (TA vs TV) on biochemical pregnancy


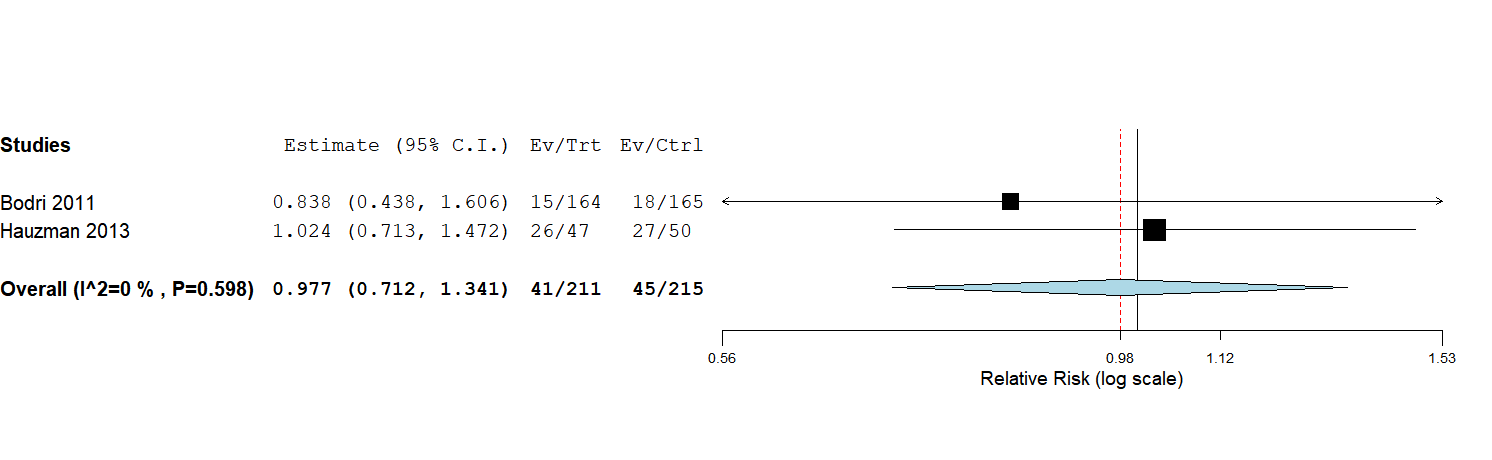


h: subgroup analysis by ultrasound route (TA vs TV) on ongoing pregnancy


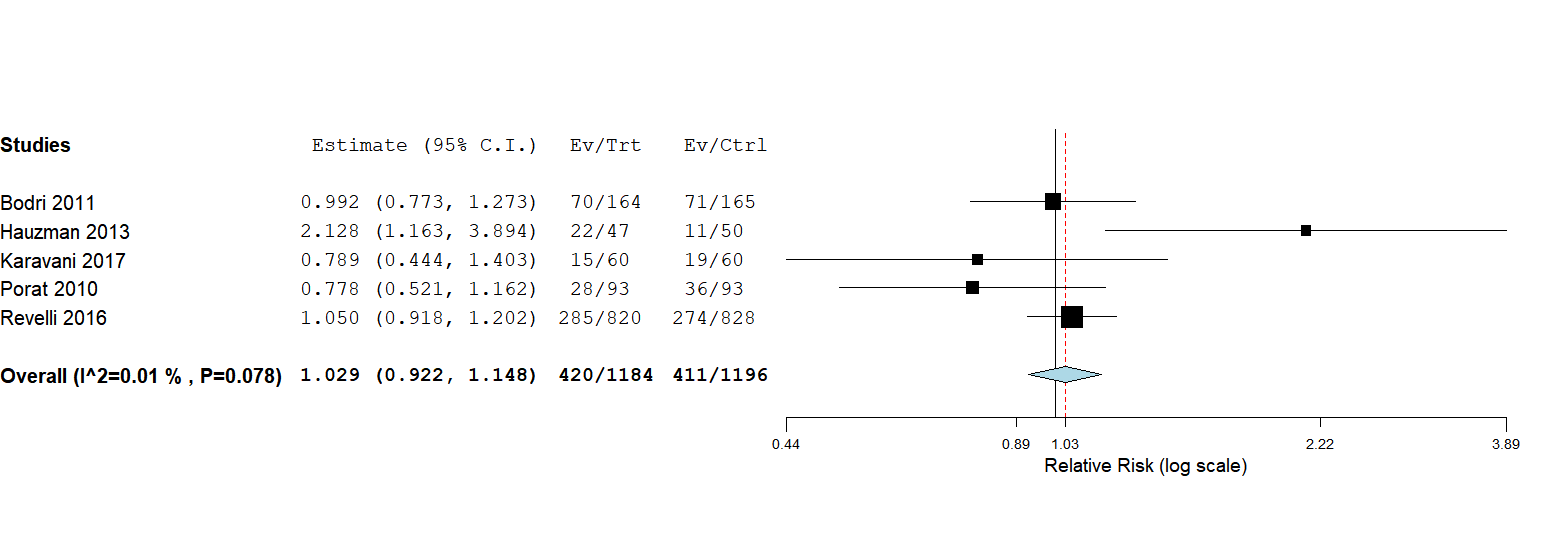


i: subgroup analysis by ultrasound route (TA vs TV) on miscarriage


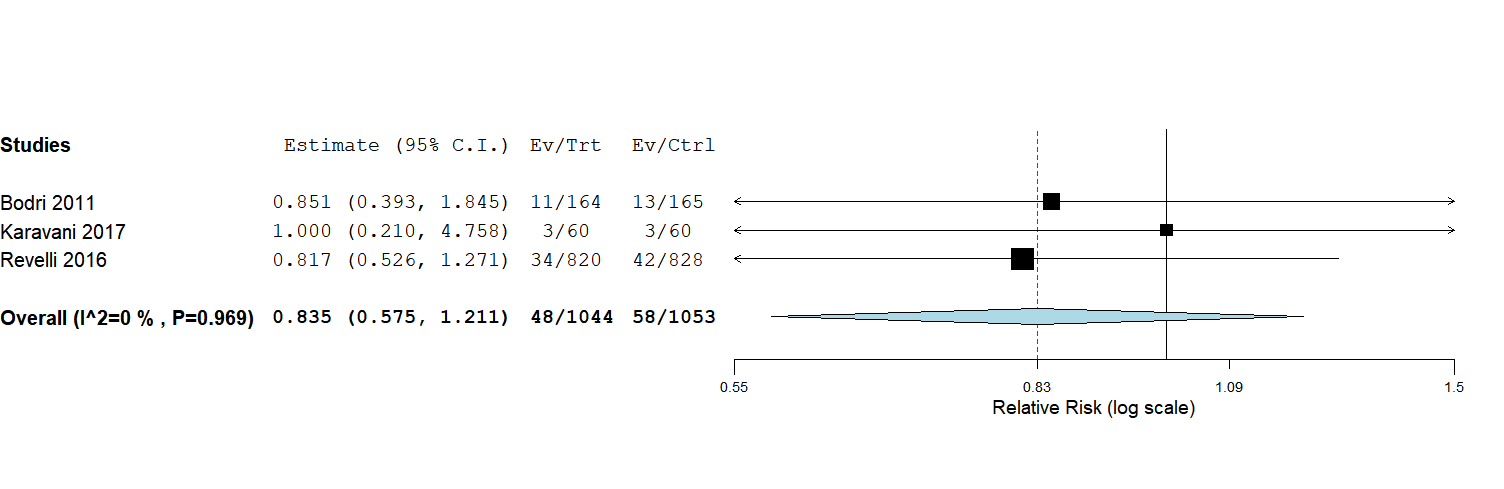


**Supplementary Figure S11:** Forest plot of the effectiveness of bladder fullness at the time of embryo transfer on reproductive outcomes.

a: clinical pregnancy


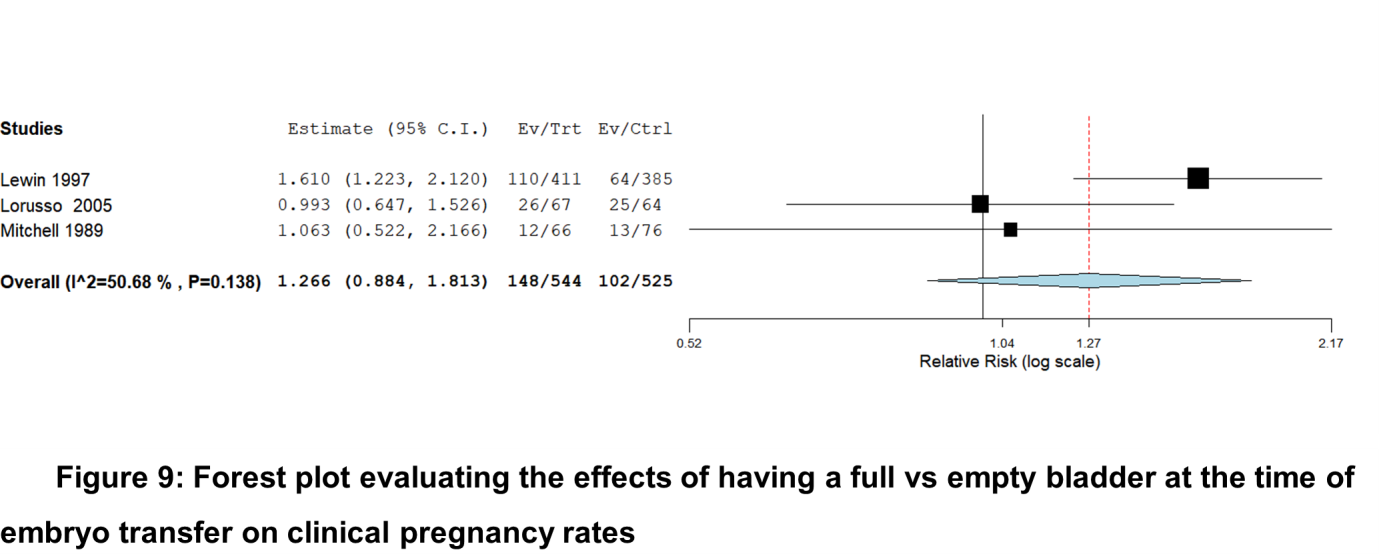


b: ongoing pregnancy


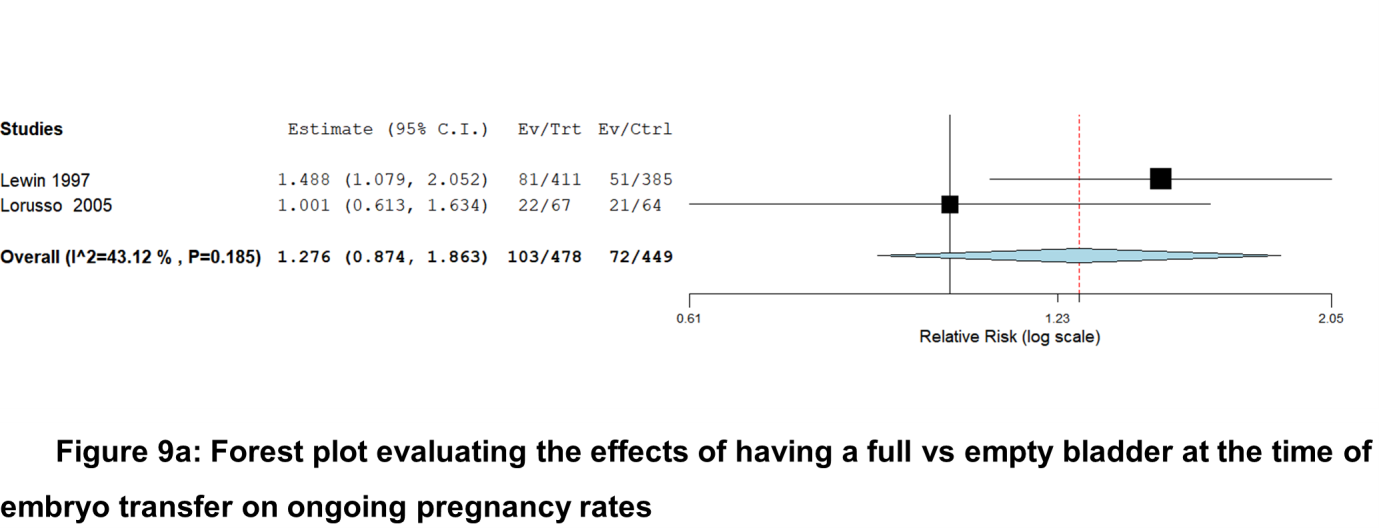


**Supplementary Figure S12:** Forest plot of the effectiveness of pressure on cervix at the time of embryo transfer on clinical pregnancy


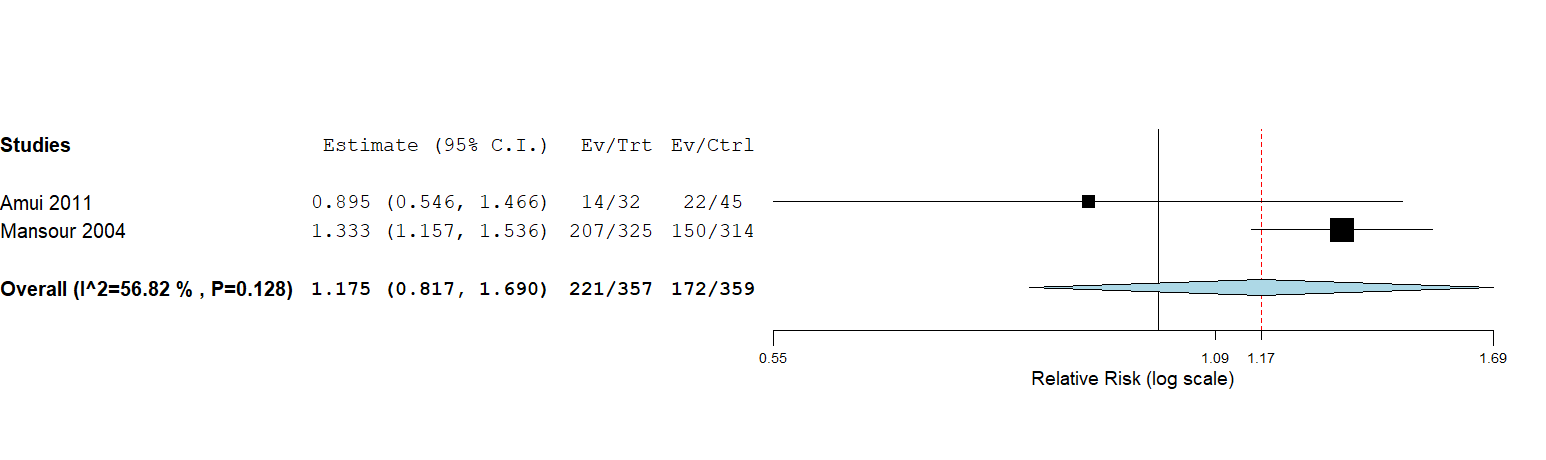


**Supplementary Figure S13:** Forest plot of the effectiveness of cervical mucus removal at the time of embryo transfer on reproductive outcomes.

a: clinical pregnancy


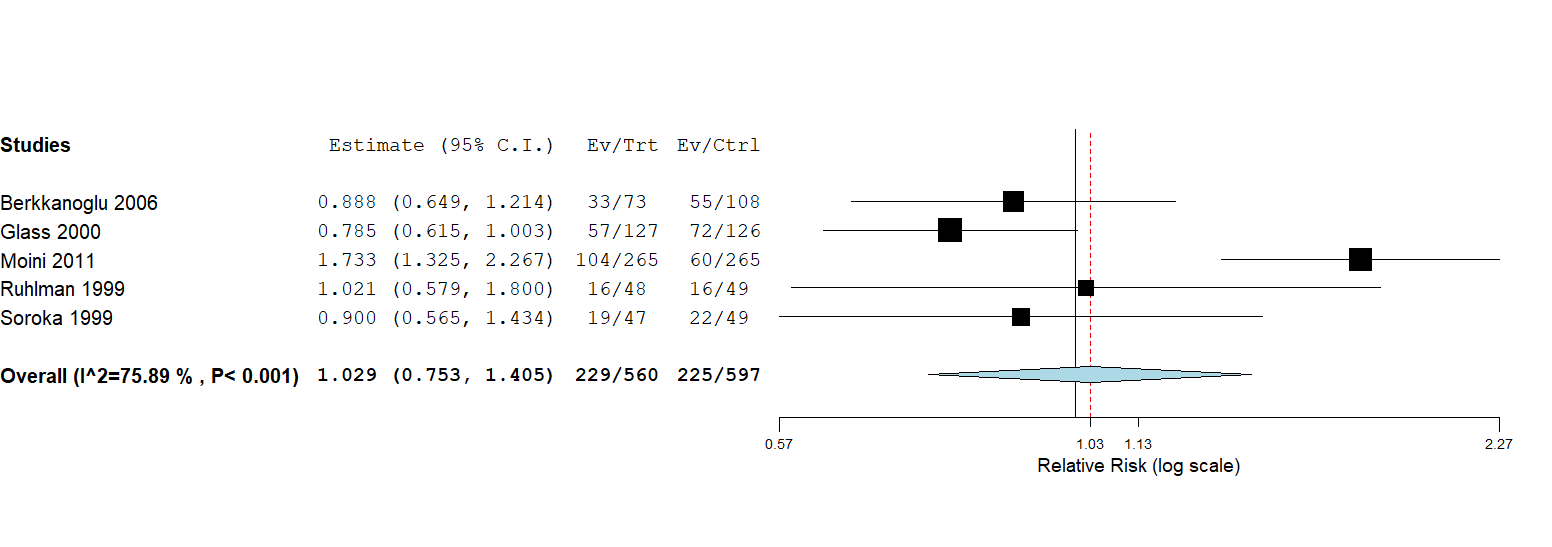


b: biochemical pregnancy

c: ongoing pregnancy


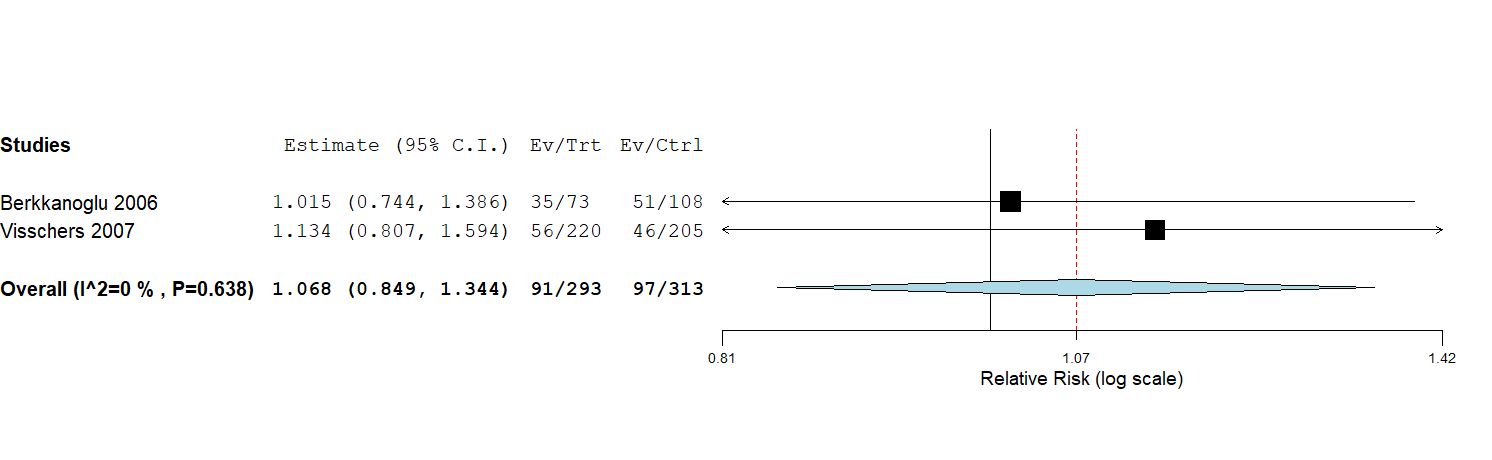


d: miscarriage


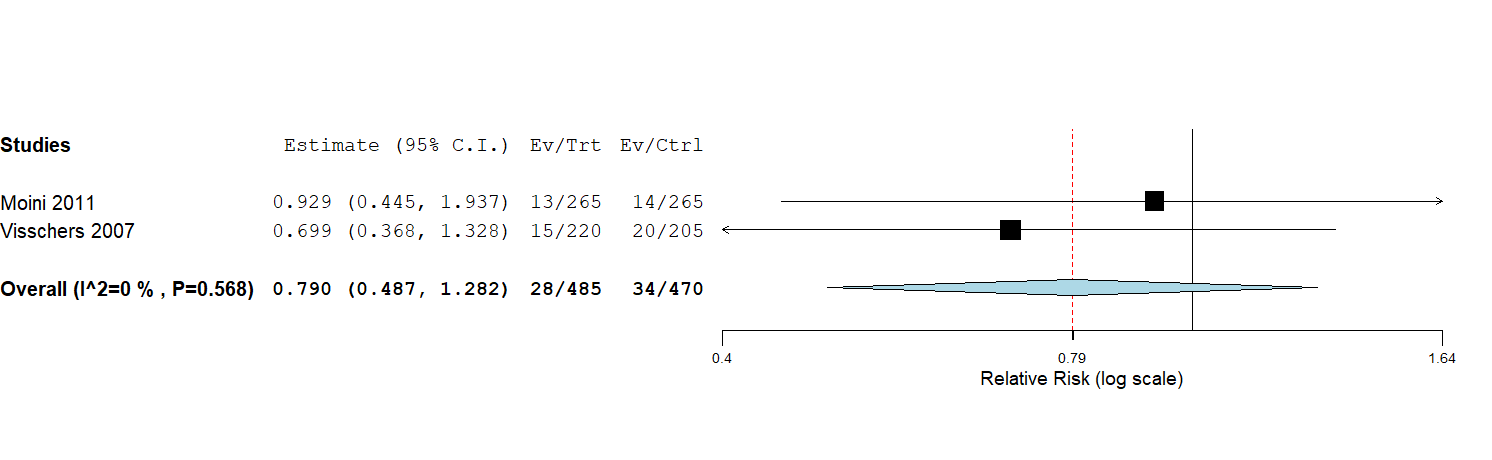


e: live birth


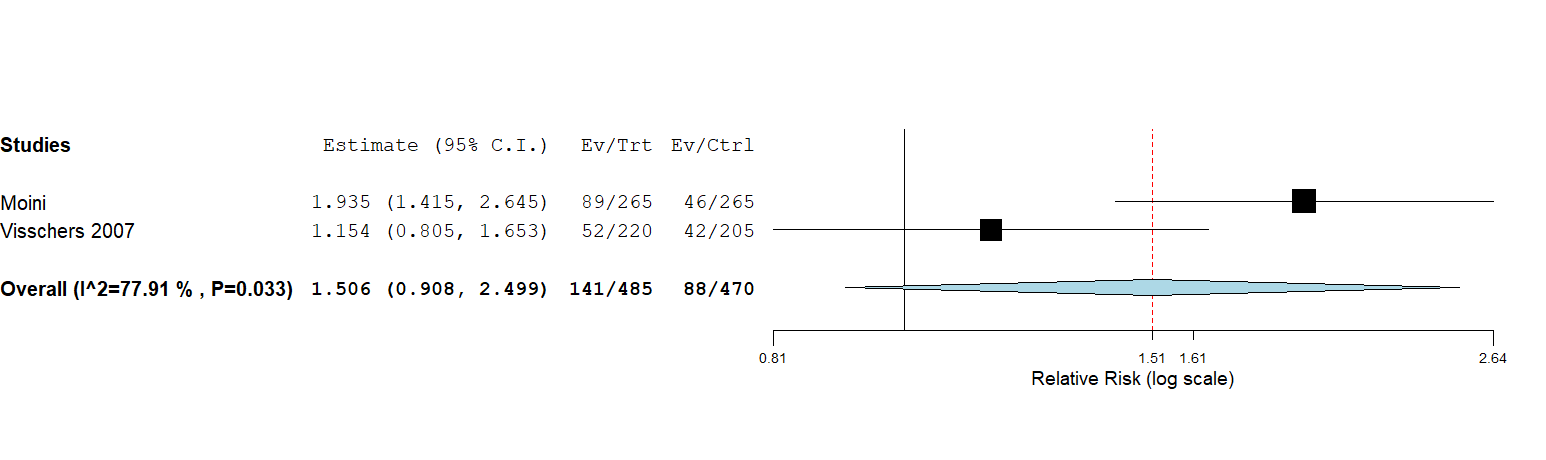


**Supplementary Figure S14:** Forest plot of the effectiveness of using Soft vs hard embryo transfer catheters on reproductive outcomes.

a: clinical pregnancy


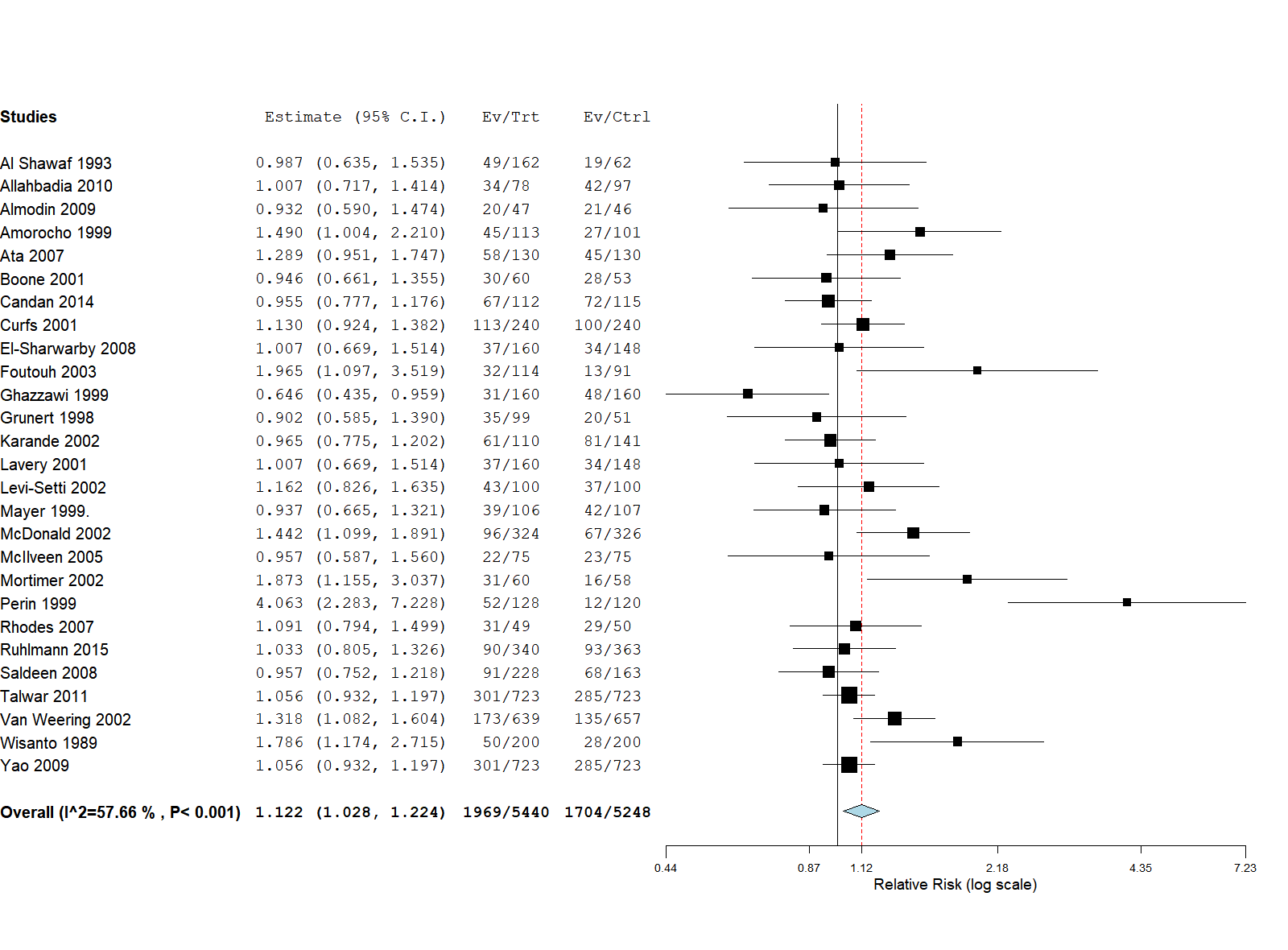


b: ongoing pregnancy


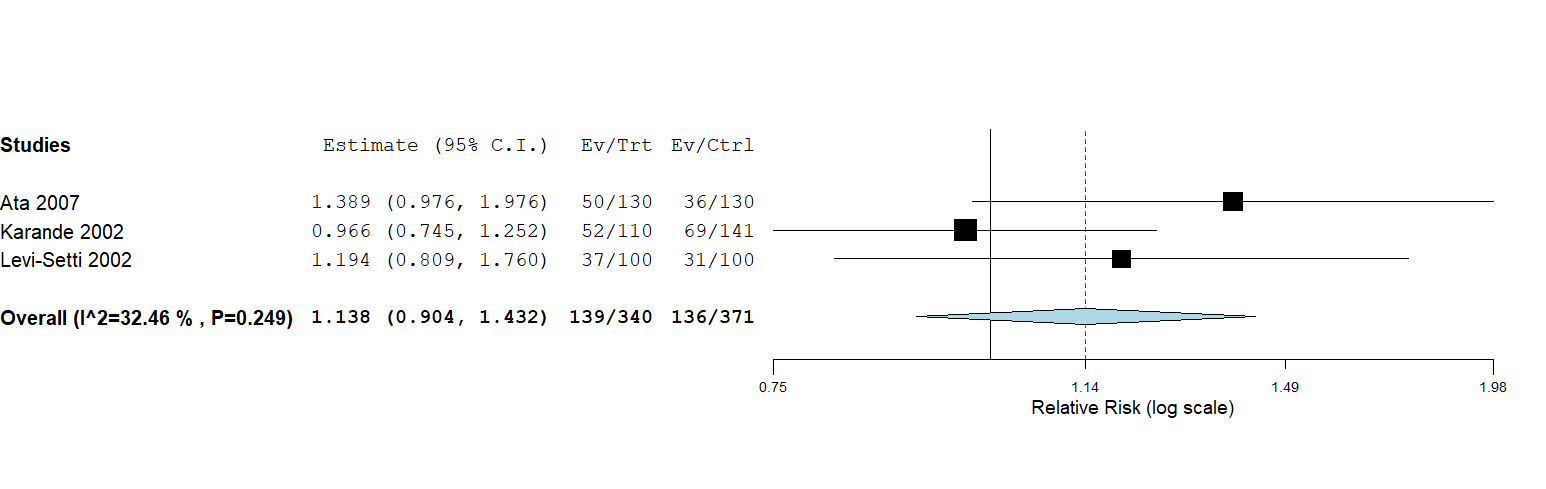


c: live birth


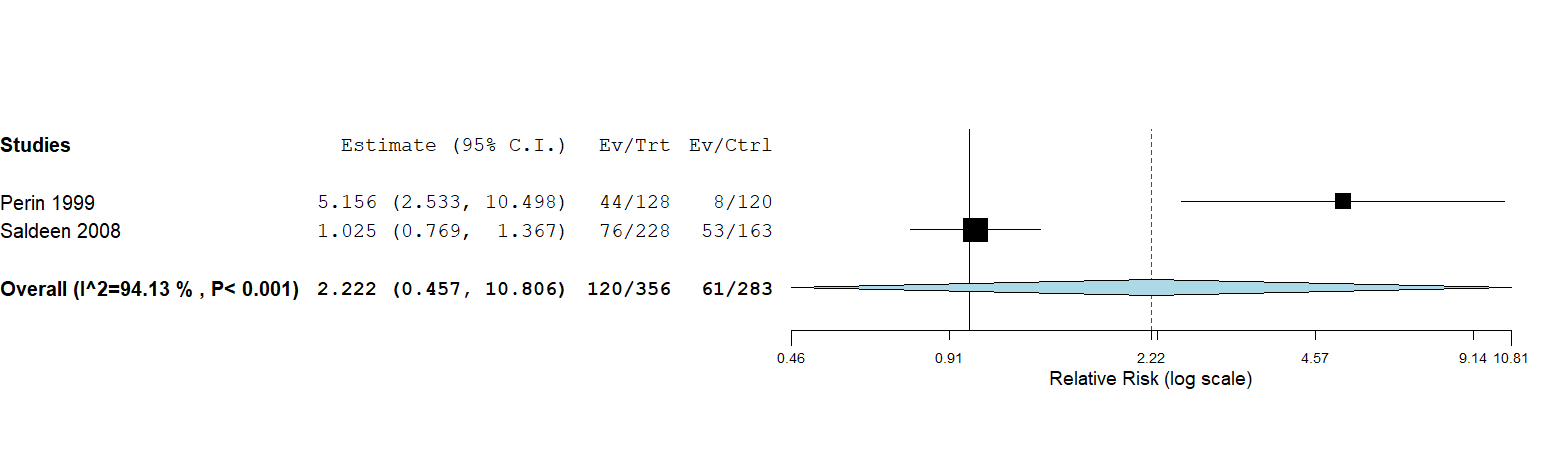


**Supplementary Figure S15:** Forest plot of the effectiveness of using air vs fluid in the embryo transfer catheter on reproductive outcomes.

a: clinical pregnancy


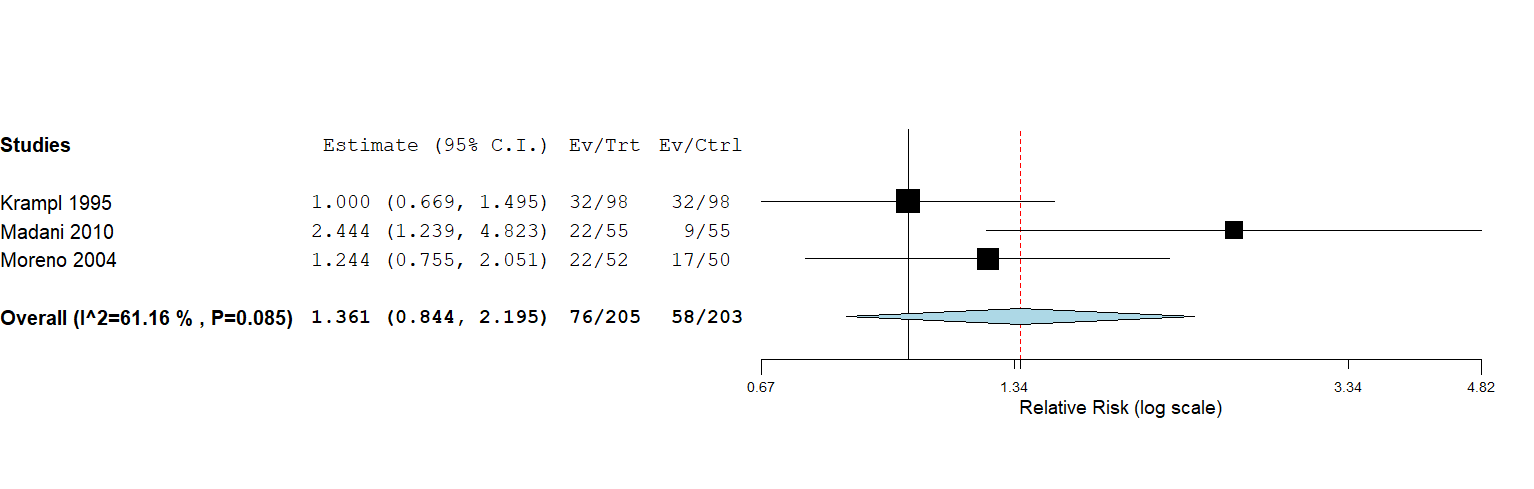


**Supplementary Figure S16:** Forest plot of the effectiveness of early vs delayed catheter withdrawal at the time of embryo transfer on clinical pregnancy.


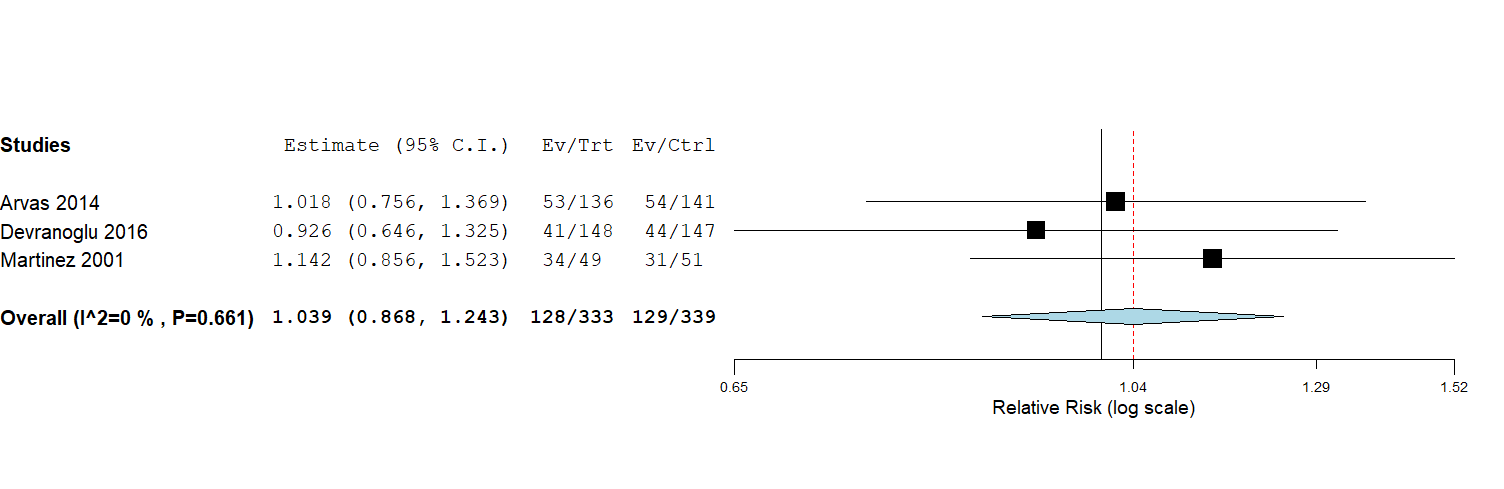


**Supplementary Figure S17:** Forest plot of the effect of the site of embryo deposition at the time of embryo transfer on reproductive outcomes.

a: clinical pregnancy


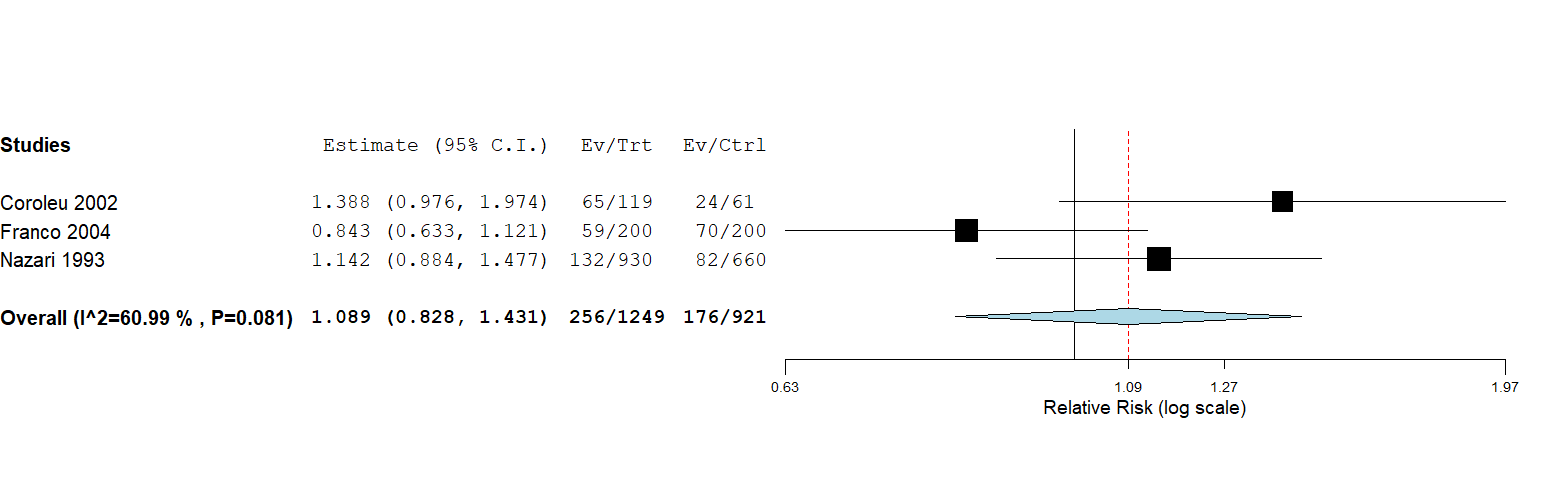


b: ongoing pregnancy


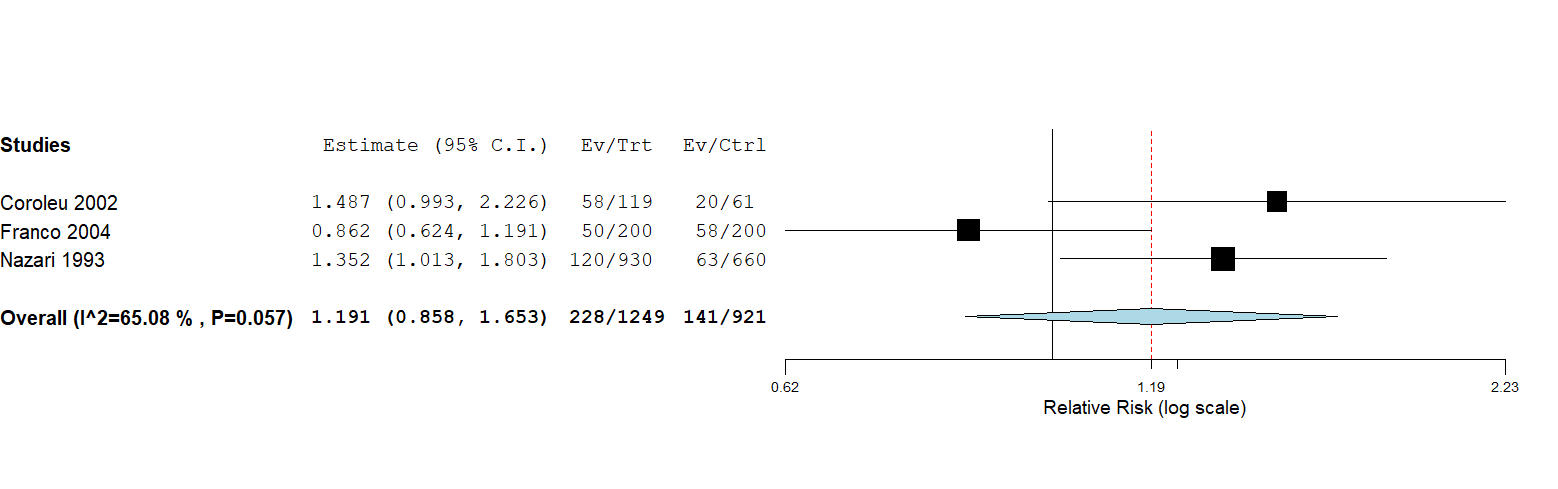


c: miscarriage


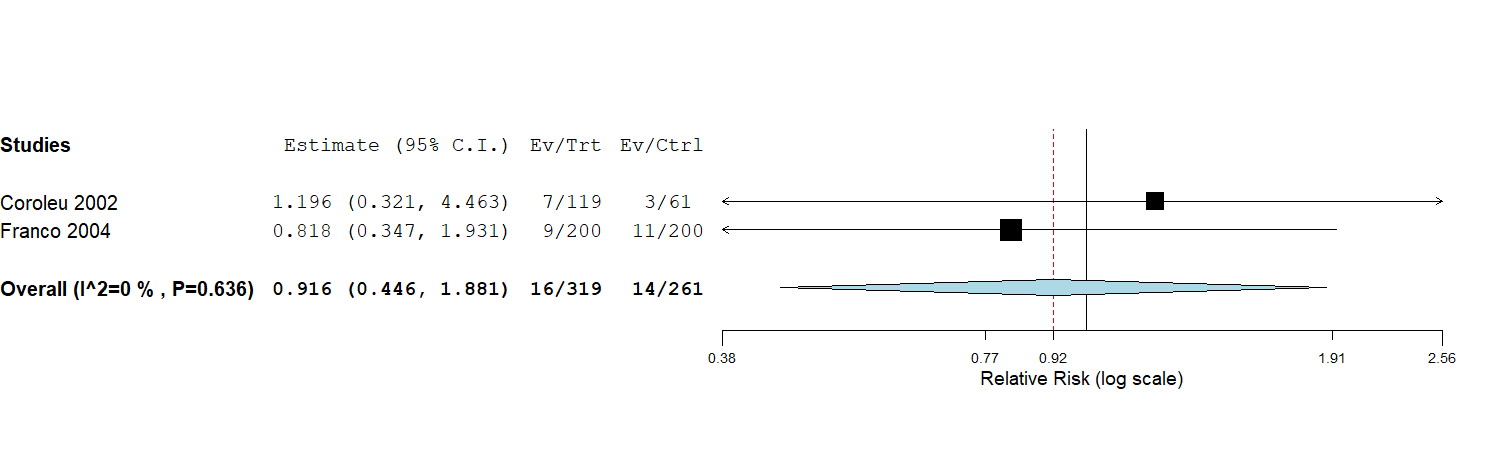


d: live birth


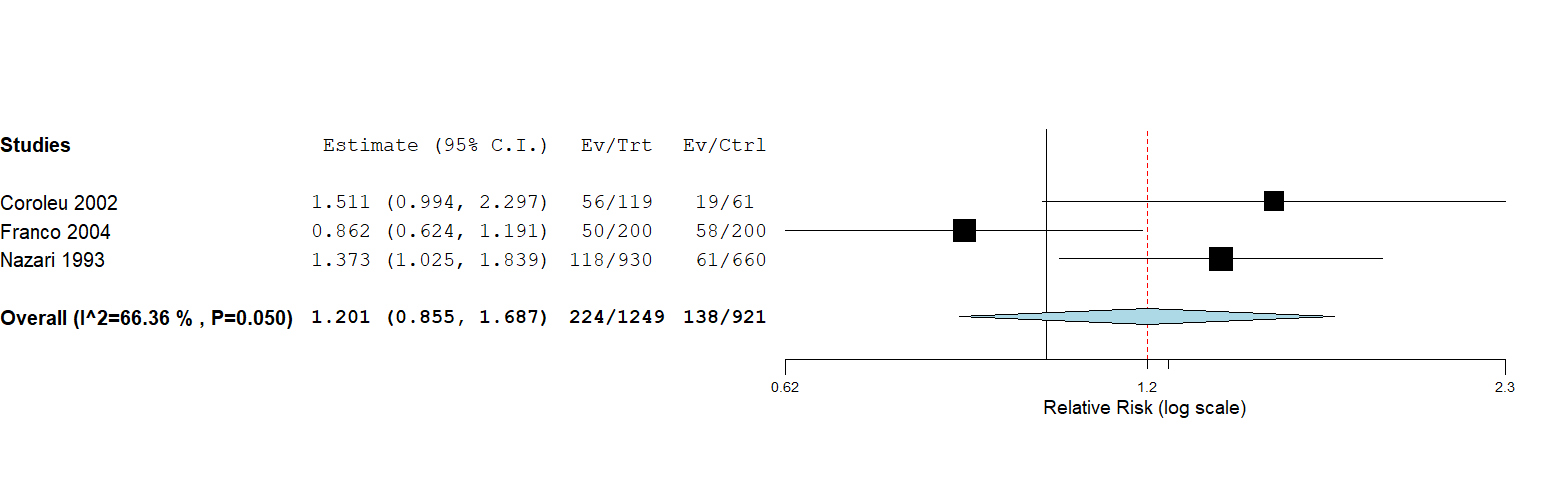


**Supplementary Figure S18:** Forest plot of the effectiveness of bed rest following embryo transfer on reproductive outcomes.

a: clinical pregnancy


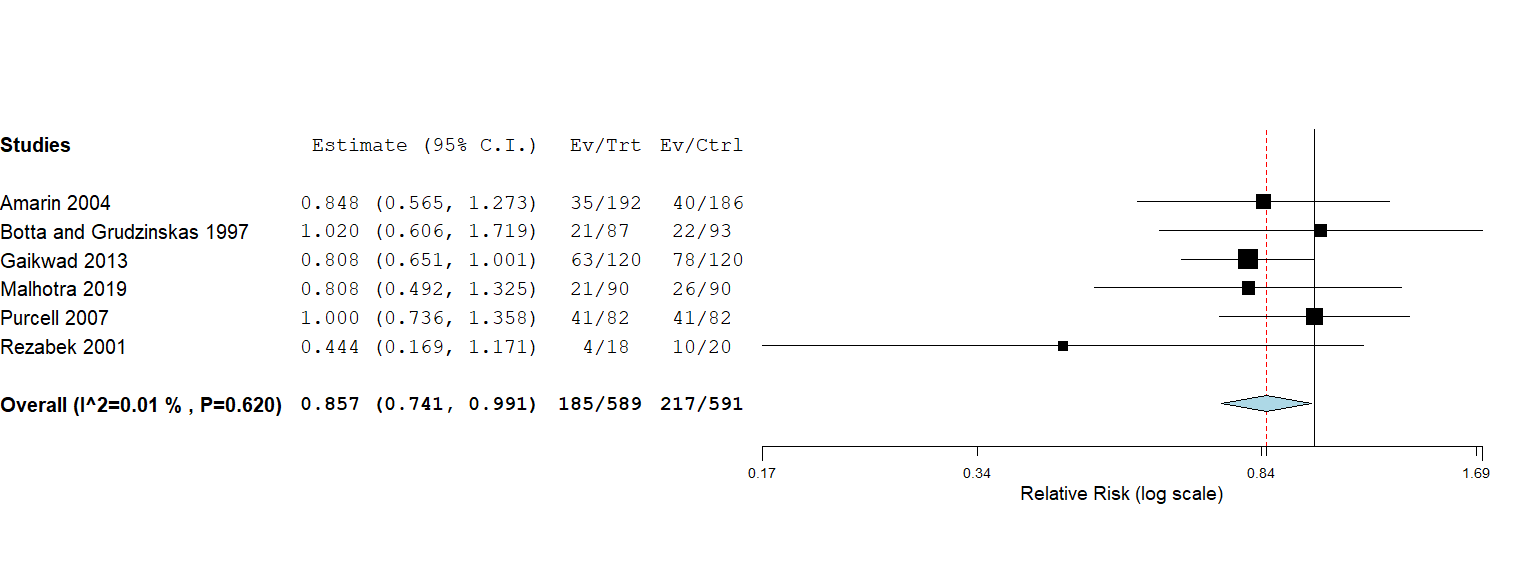


b: biochemical pregnancy


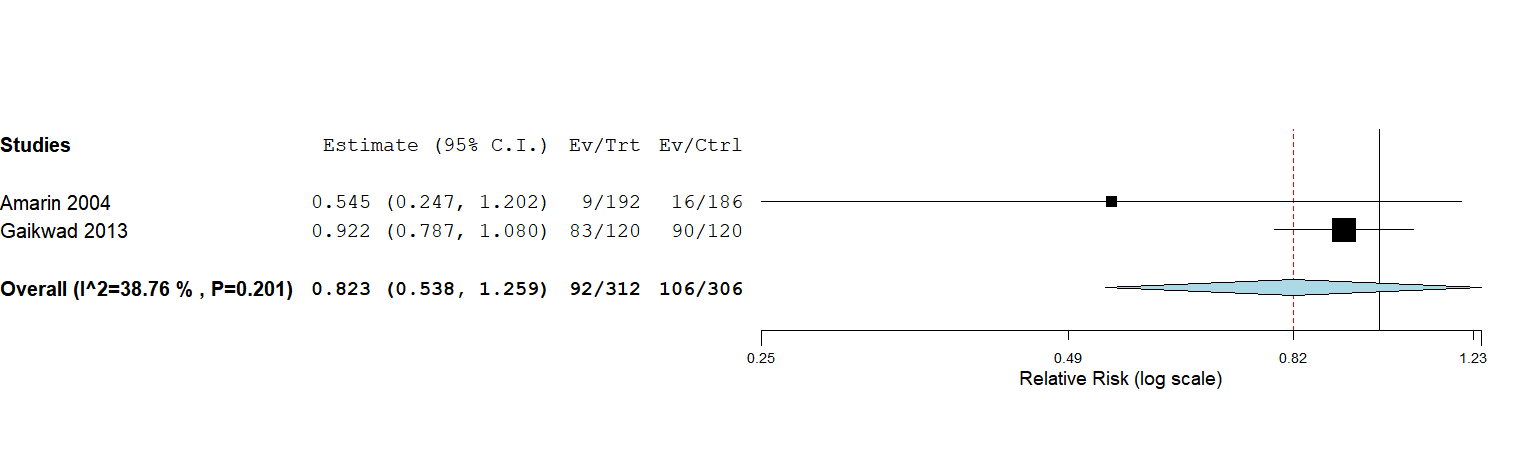


c: ongoing pregnancy


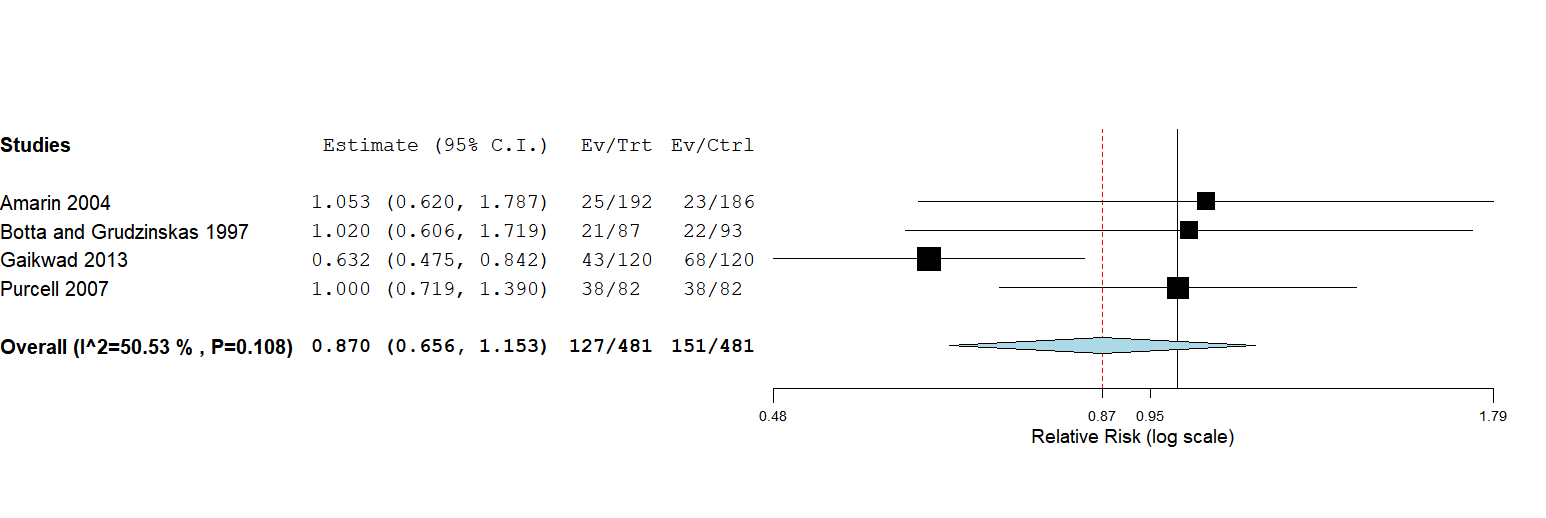


d: miscarriage


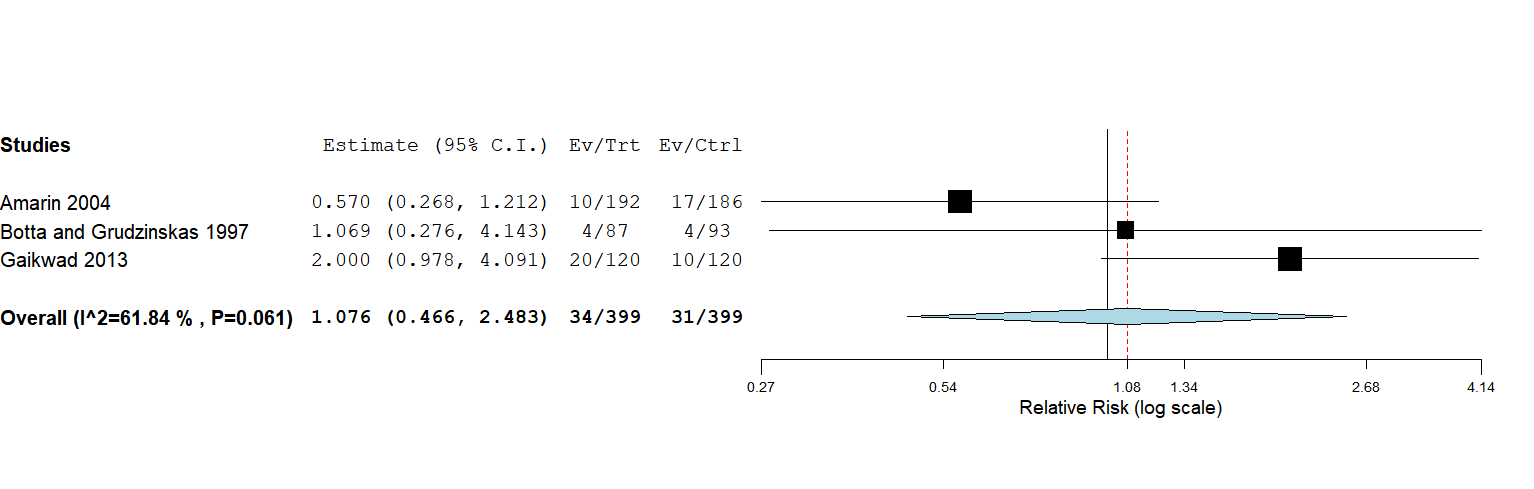


e: live birth


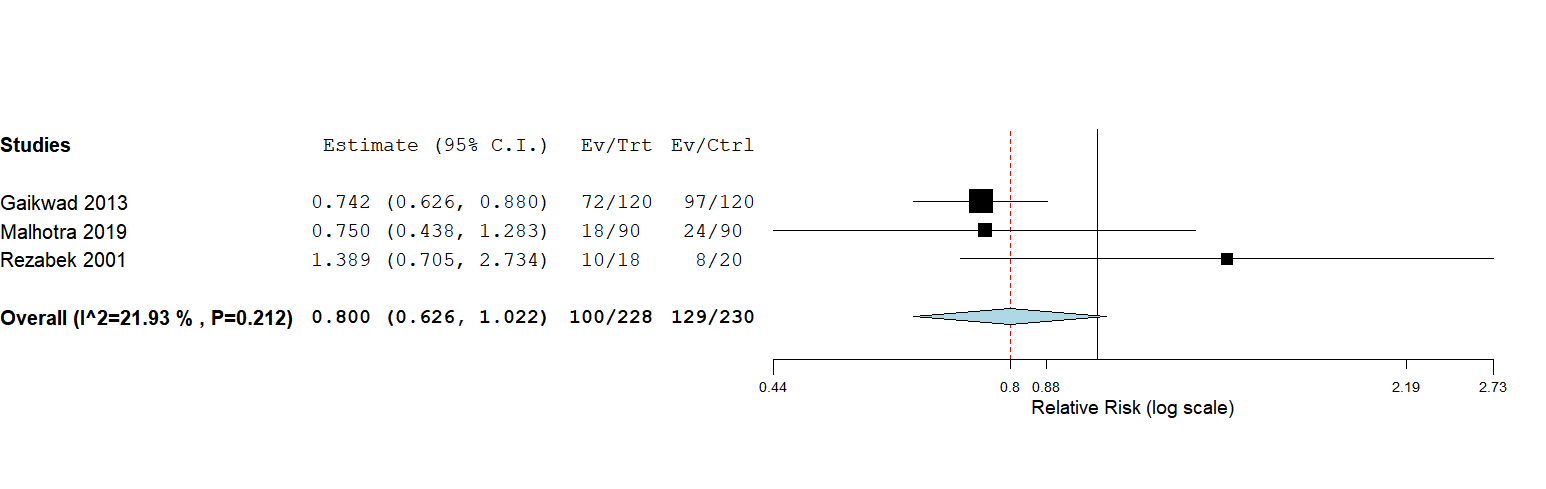


**Supplementary Figure S19:** Forest plot comparing the difference in reproductive outcomes following embryo transfer performed by nurse vs doctor.

a: clinical pregnancy


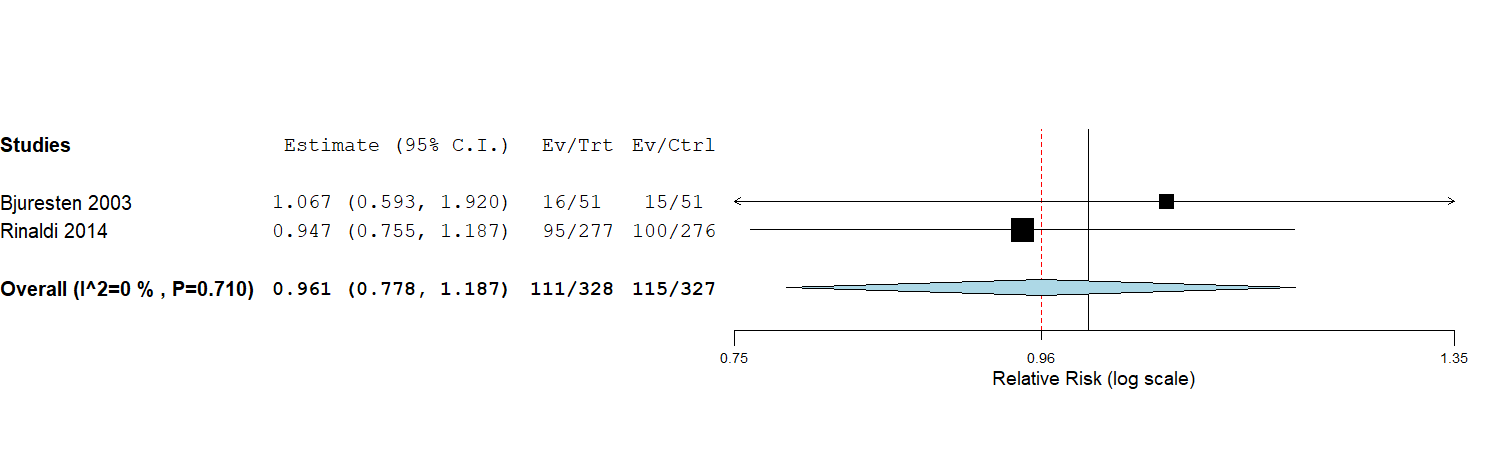


**Supplementary Figure S20:** Forest plot of the effectiveness of acupuncture vs shame acupuncture

performed before embryo transfer on reproductive outcomes.

a: clinical pregnancy


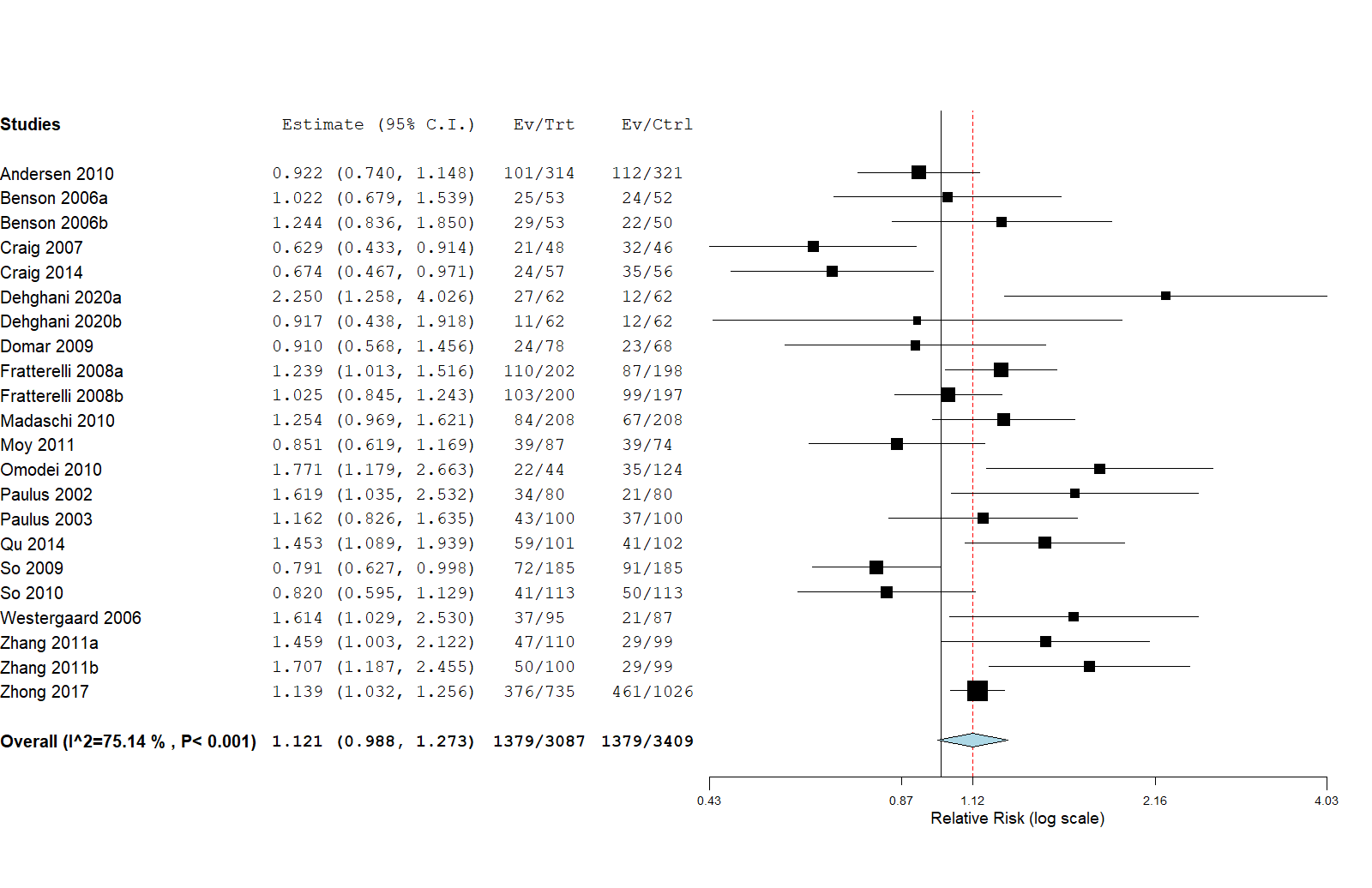


b: biochemical pregnancy


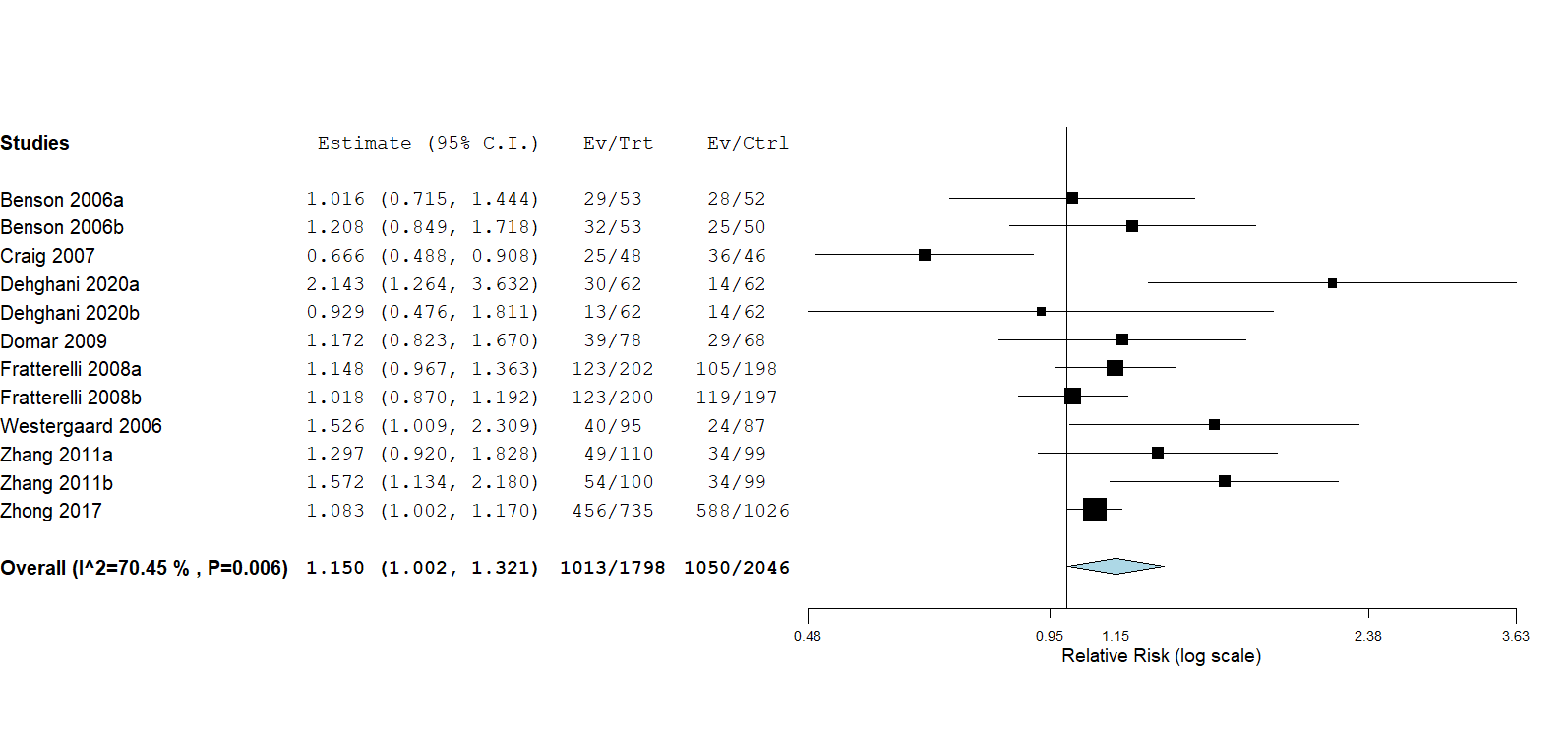


c: ongoing pregnancy


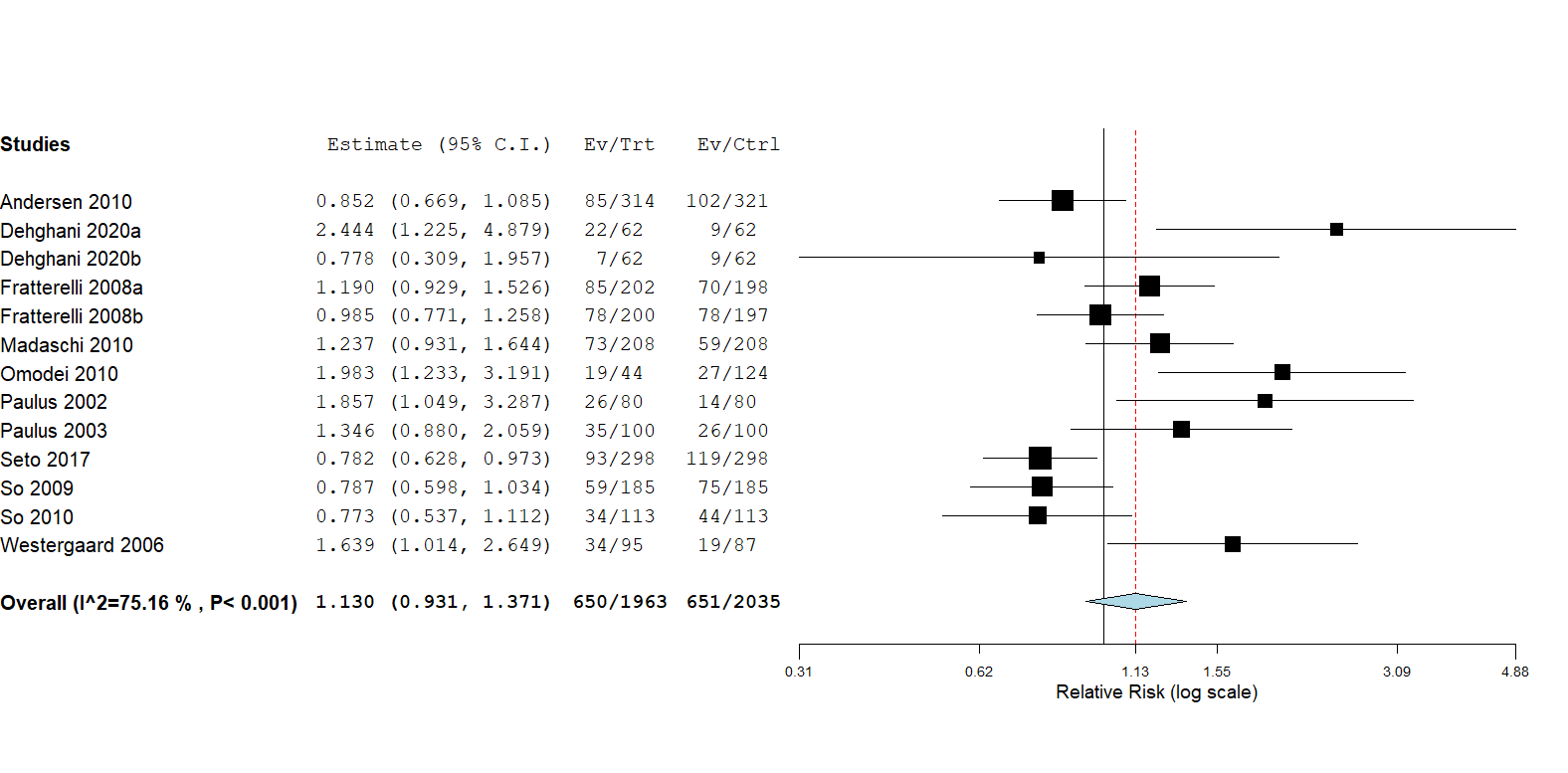


d: miscarriage


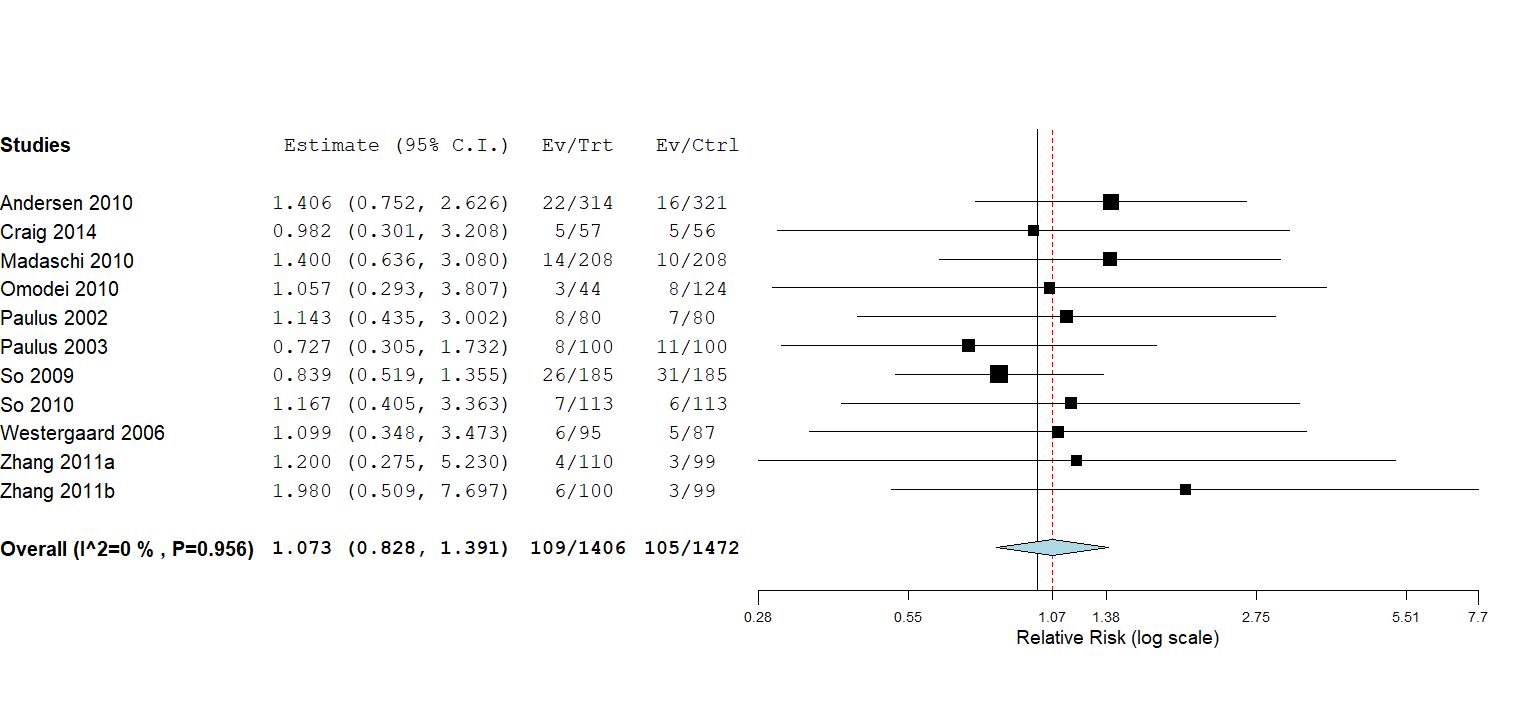


e: live birth


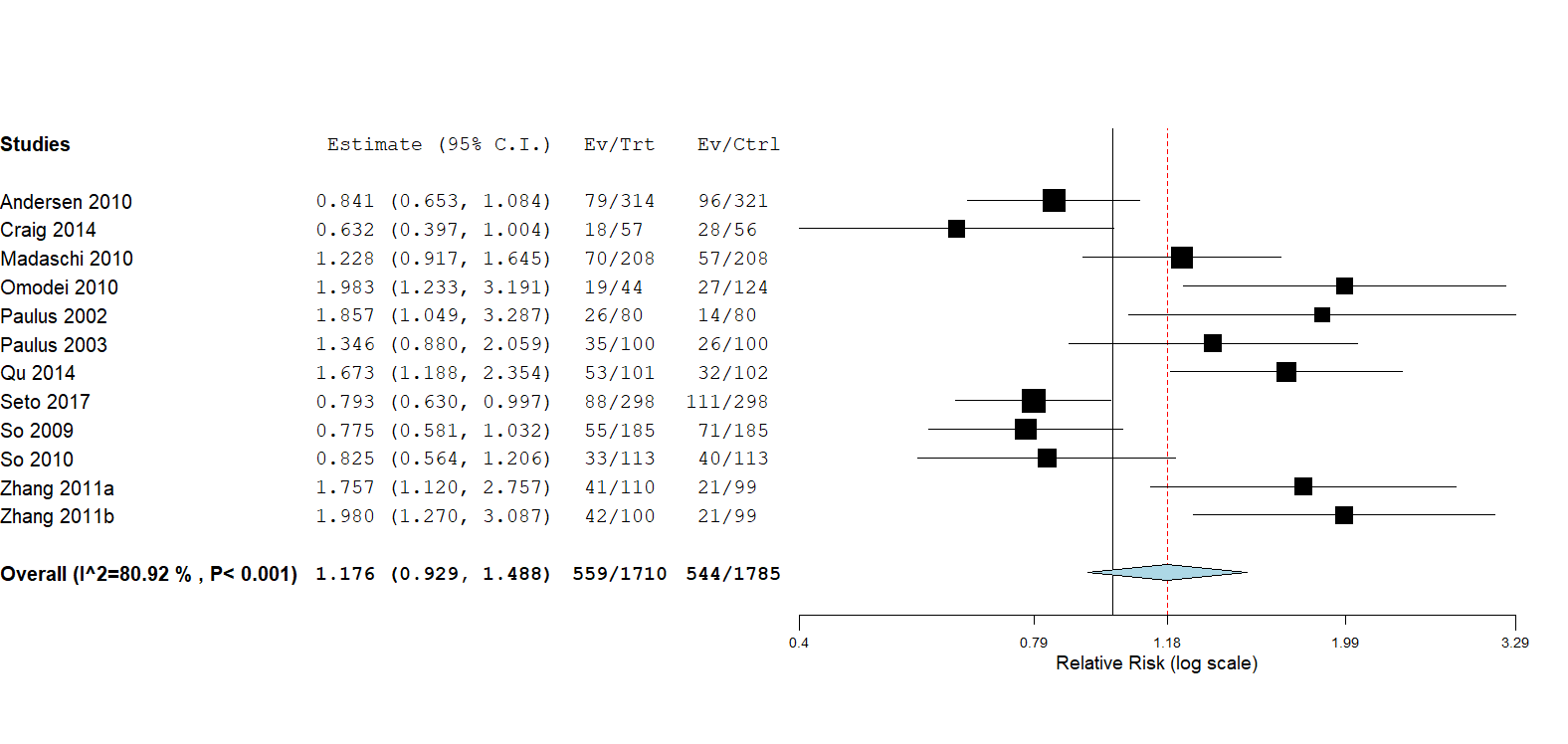


f: subgroup analysis by type of acupuncture on clinical pregnancy


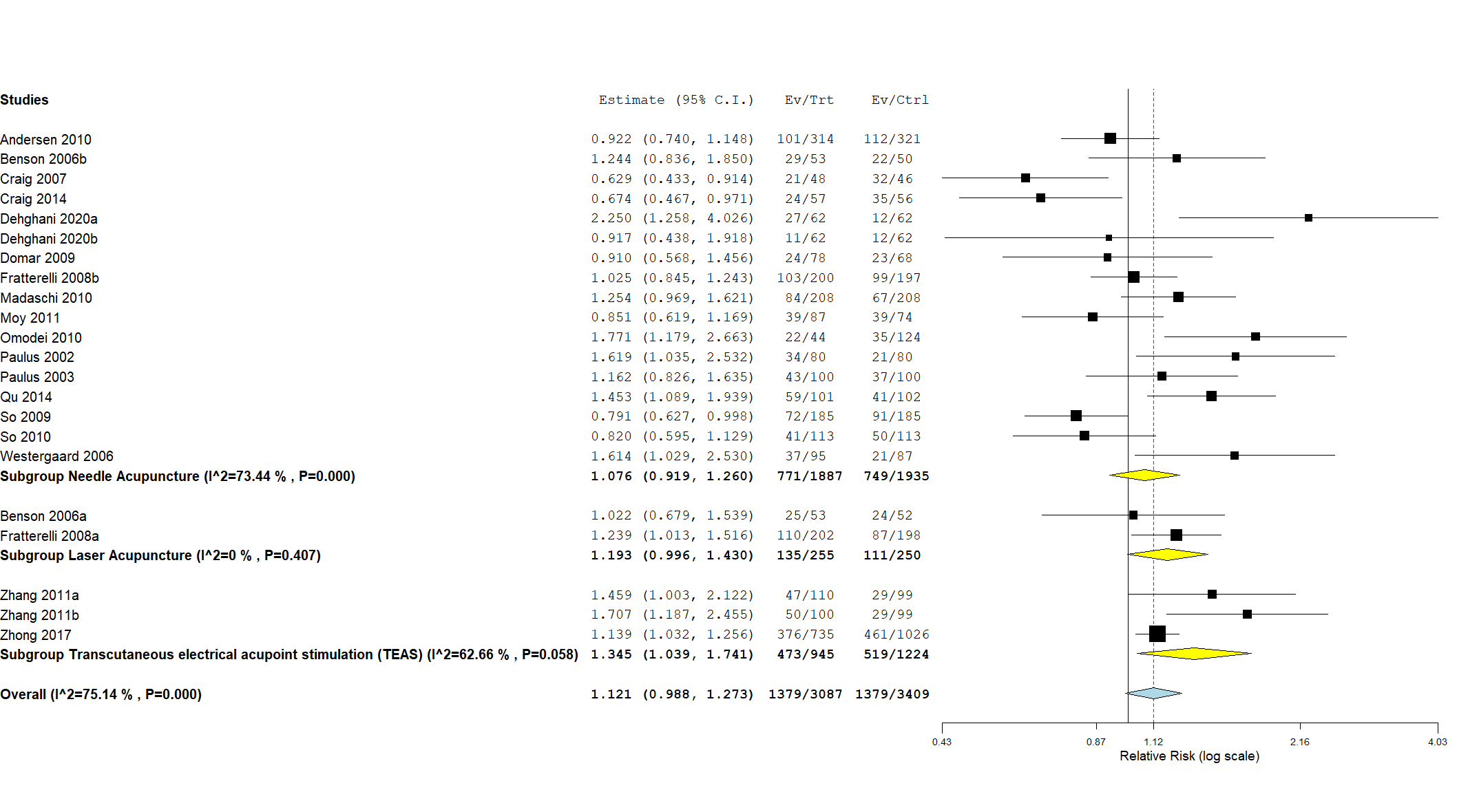


g: subgroup analysis by comparison (sham acupuncture or no treatment) on clinical pregnancy


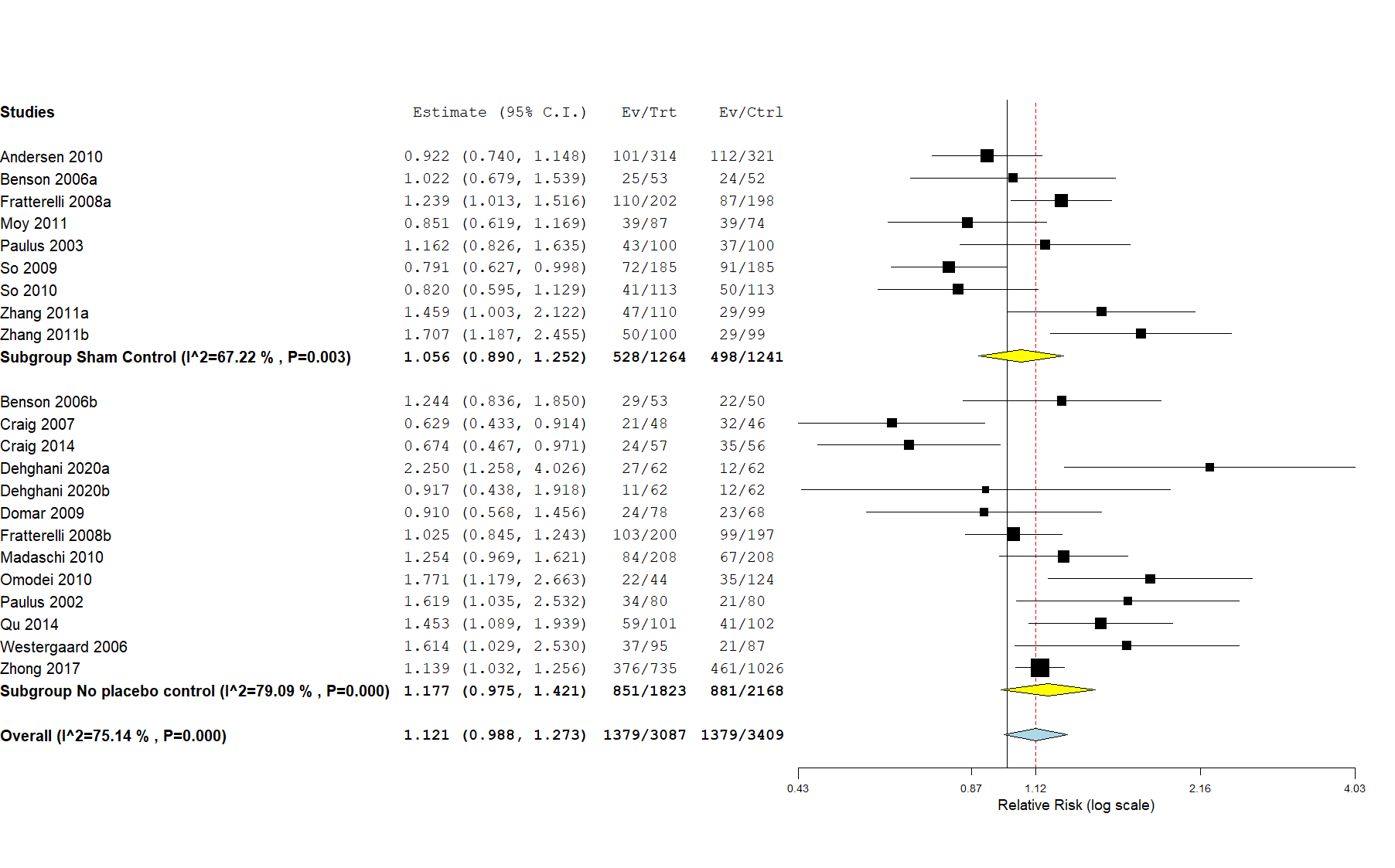


**Supplementary Figure S21:** Forest plot of the effectiveness of music at the time of embryo transfer on clinical pregnancy


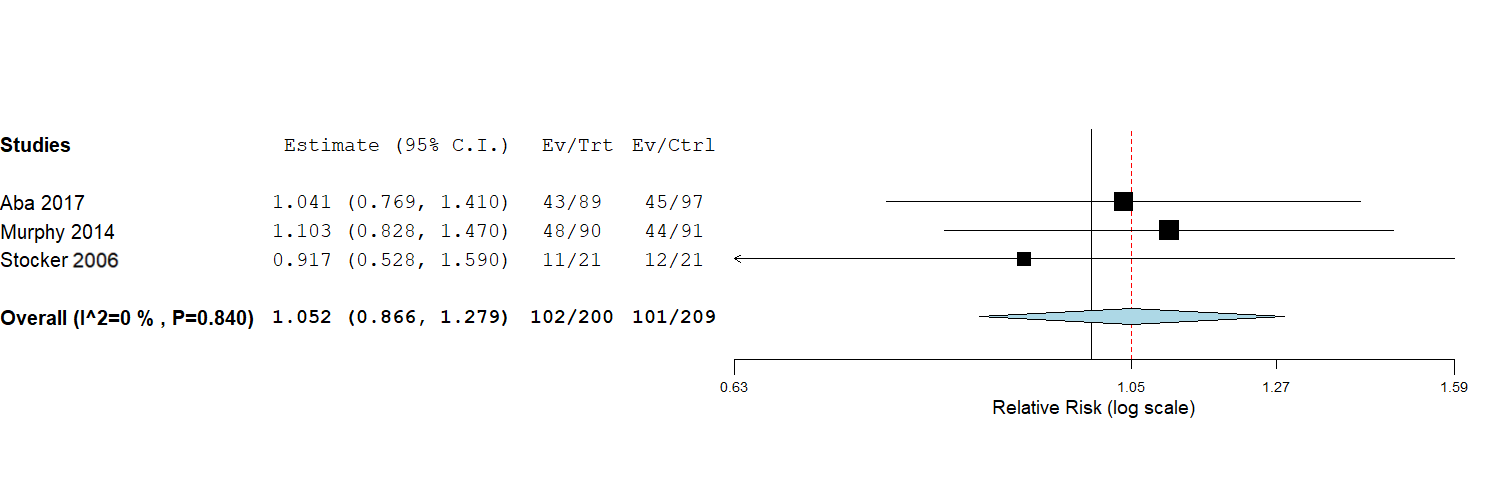


**Supplementary Figure S22:** Forest plot of the effectiveness of mindfulness at the time of embryo transfer on reproductive outcomes.

a: clinical pregnancy


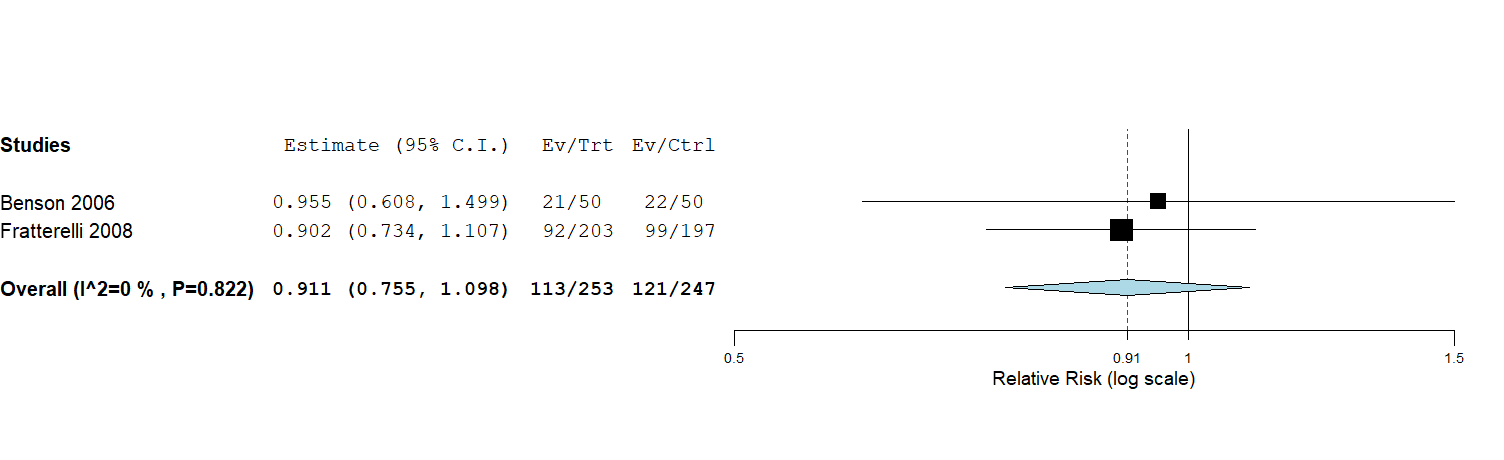


b: biochemical pregnancy


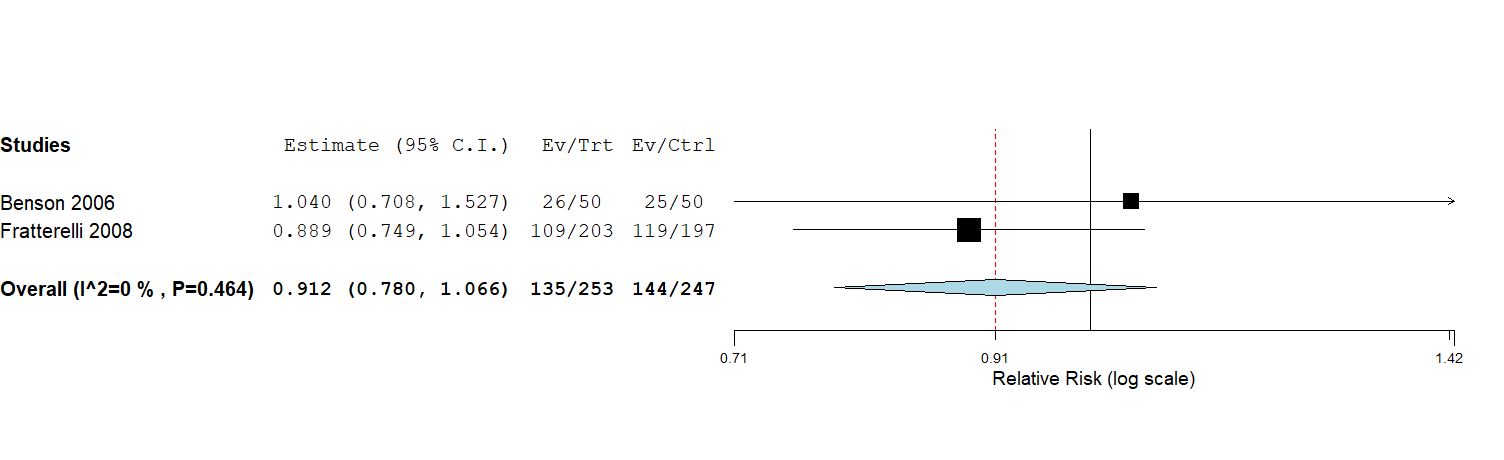


**Supplementary Appendix S1:** Literature search strategy

Employed MeSh Terms and keywords per search strategy:

Generic search terms

Search for pharmacological interventions

Studies included in previous systematic reviews

3750 citations

3685 de-duplicated citations

Screening bibliographies

catheter, soft, hard, ultrasound, echography, guidance, fundus, uterine contraction, catheter, soft, hard, echogenic, bent, straight, bed rest, mindfulness, relaxation, meditation, music, cervix, cervical mucus, pressure, bladder, operator, nurse, doctor, physician

anti-biotic, antibiotic, calcium channel blocker, nifedipine, atosiban, oxytocin inhibitor, oxytocin antagonist, prostaglandin, anticholinergic, Human chorionic gonadotropin, hcg, beta hcg, bhcg, Nonsteroidal, anti-inflammatory NSAIDs, hyaluronic acid, embryo glue, Granulocyte colony-stimulating factor GCSF, seminal fluid

Search for non-pharmacological interventions

Embryo, transfer, deposition, fetus, blastocyst, cleavage, assisted conception, assisted reproduction technology, ART, in vitro fertili?ation, IVF, intra-cytoplasmic sperm injection, ICSI, women, female

**Bibliography:**

1. Andersen D, Løssl K, Andersen AN, Fürbringer J, Bach H, Simonsen J, et al. Acupuncture on the day of embryo transfer: a randomized controlled trial of 635 patients. Reproductive BioMedicine Online. 2010 Sep 1;21(3):366–72.

2. Benson MR, Elkind-Hirsch KE, Theall A, Fong K, Hogan RB, Scott RT. P-18: Impact of acupuncture before and after embryo transfer on the outcome of in vitro fertilization cycles: A prospective single blind randomized study. Fertility and Sterility. 2006 Sep 1;86(3):S135.

3. Craig LB, Criniti AR, Hansen KR, Marshall LA, Soules MR. Acupuncture lowers pregnancy rates when performed before and after embryo transfer. Fertility and Sterility. 2007 Sep 1;88:S40.

4. Craig L, Rubin L, Peck J, Anderson M, Marshall L, Soules M. Acupuncture performed before and after embryo transfer: a randomized controlled trial. J Reprod Med. 2014;59:313–20.

5. Dehghani AS, Homayouni K, Kanannejad Z, Kanannejad Z. The effect of acupuncture on the day of embryo transfer on the in vitro fertilization outcomes: An RCT. International Journal of Reproductive BioMedicine. 2020 Mar 1;18(3):209–14.

6. Domar AD, Meshay I, Kelliher J, Alper M, Powers RD. The impact of acupuncture on in vitro fertilization outcome. Fertility and Sterility. 2009 Mar;91(3):723–6.

7. Fratterelli JL, Leondires MR, Fong K, Theall A, Locatelli S, Scott RT. Laser acupuncture before and after embryo transfer improves ART delivery rates: results of a prospective randomized double-blinded placebo controlled five-armed trial involving 1000 patients. Fertility and Sterility. 2008 Sep 1;90:S105.

8. Madaschi C, Braga D, Rde CF, Iaconelli A, Borges E. Effect of acupuncture on assisted reproduction treatment outcomes. Acupunct Med. 2010;28:180–4.

9. Moy I, Milad M, Barnes R, Confino E, Kazer R, Zhang X. Randomized controlled trial: effects of acupuncture on pregnancy rates in women undergoingin vitro fertilization. Fertil Steril. 2011;95:583–7.

10. Ng EHY, So EWS, Li RHW, Yeung WSB, Ho PC. O-176 Effect of non-invasive acupuncture on the pregnancy outcome of IVF treatmentóa randomized controlled study. Human Reproduction. 2011 Jan 1;26(suppl_1):i69–71.

11. Omodei U, Piccioni G, Tombesi S, Dordoni D, Fallo L, Ghilardi F. Effect of acupuncture on rates of pregnancy among women undergoingin vitro fertilization. Fertil Steril. 2010;94:P-266.

12. Paulus W, Zhang M, Strehler E, El-Danasouri I, Sterzik K. Influence of acupuncture on the pregnancy rate in patients who undergo assisted reproduction therapy. Fertil Steril. 2002;77:721–4.

13. Paulus W, Zhang M, Strehler E, Seybold B, Sterzik K. Placebo-controlled trial of acupuncture effects in assisted reproduction therapy. Hum Reprod. 2003;18:18–9.

14. Qu F, Zhang D, Chen L-T, Wang F-F, Pan J-X, Zhu Y-M, et al. Auricular Acupressure Reduces Anxiety Levels and Improves Outcomes of in Vitro Fertilization: A Prospective, Randomized and Controlled Study. Scientific Reports. 2014 May 22;4.

15. Seto MTY, Cheung KW, Lo TK, Ng EHY. Pregnancy outcomes of women randomized to receive real versus placebo acupuncture on the day of fresh or frozen-thawed embryo transfer. European Journal of Obstetrics and Gynecology and Reproductive Biology. 2017 Nov 1;218:119–22.

16. So E, Ng E, Wong Y, Lau E, Yeung W, Ho P. A randomized double blind comparison of real and placebo acupuncture in IVF treatment. Hum Reprod. 2009;24:341–8.

17. So E, Ng E, Wong Y, Yeung W, Ho P. Acupuncture for frozen-thawed embryo transfer cycles: a double-blind randomized controlled trial. Reprod Biomed Online. 2010;20:814–21.

18. Westergaard L, Mao Q, Krogslund M, Sandrini S, Lenz S, Grinsted J. Acupuncture on the day of embryo transfer significantly improves the reproductive outcome in infertile women: a prospective, randomized trial. Fertil Steril. 2006;85:1341–6.

19. Zhang R, Feng X-J, Guan Q, Cui W, Zheng Y, Sun W, et al. Increase of success rate for women undergoing embryo transfer by transcutaneous electrical acupoint stimulation: a prospective randomized placebo-controlled study. Fertility and Sterility. 2011 Oct 1;96(4):912–6.

20. Zhong J, Zhang L. [Transcutaneous electrical acupoint stimulation for pregnancy of in vitro fertilization-embryo transfer]. Zhongguo zhen jiu = Chinese acupuncture & moxibustion. 2017;37(3):253–5.

21. Krampl E, Zegermacher G, Eichler C, Obruca A, Strohmer H, Feichtinger W. Air in the uterine cavity after embryo transfer. Fertility and Sterility. 1995 Feb 1;63(2):366–70.

22. Madani T, Ashrafi M, Jahangiri N, Abadi AB, Lankarani N. Improvement of pregnancy rate by modification of embryo transfer technique: A randomized clinical trial. Fertility and Sterility. 2010 Nov;94(6):2424–6.

23. Moreno V, Balasch J, Vidal E, Calafell JM, Cívico S, Vanrell JA. Air in the transfer catheter does not affect the success of embryo transfer. Fertility and Sterility. 2004 May;81(5):1366–70.

24. Brook N, Khalaf Y, Coomarasamy A, Edgeworth J, Braude P. A randomized controlled trial of prophylactic antibiotics (co-amoxiclav) prior to embryo transfer. Human Reproduction. 2006 Nov 1;21(11):2911–5.

25. Peikrishvili R, Evrard B, Pouly JL, Janny L. L’antibiothérapie prophylactique (amoxicilline + acide clavulanique) avant transfert pour fécondation in vitro est inutile: Résultats d’une étude randomisée. Journal de Gynécologie Obstétrique et Biologie de la Reproduction. 2004 Dec 1;33(8):713–9.

26. Ahn J, Kim C, Kim S, Jeon G, Kim S, Chae H. Effects of Administration of Oxytocin Antagonist on Implantation and Pregnancy Rates in Patients with Repeated Failure of IVF/ICSI Treatment -Clinical and Experimental Reproductive Medicine. Korean Journal of Reproductive Medicine. 2009;36:275–81.

27. Bosch E, Aasted H, Klein BM, Arce J-C. A randomized, double-blind, placebo-controlled, multi-center, phase 2 trial to investigate the effect of barusiban on implantation in IVF/ICSI patients. Fertility and Sterility. 2019 Sep 1;112(3):e179.

28. He Y, Wu H, He X, Xing Q, Zhou P, Cao Y, et al. Administration of atosiban in patients with endometriosis undergoing frozen–thawed embryo transfer: a prospective, randomized study. Fertility and Sterility. 2016 Aug 1;106(2):416–22.

29. Hebisha SA, Aboelazm BA, Adel HM, Ahmed AI. Impact of the oxytocin receptor antagonist (ATOSIBAN) administered shortly before embryo transfer on pregnancy outcome after intracytoplasmic sperm injection (ICSI). Fertility and Sterility. 2016 Sep 1;106(3):e88–9.

30. Moraloglu O, Tonguc E, Var T, Zeyrek T, Batioglu S. Treatment with oxytocin antagonists before embryo transfer may increase implantation rates after IVF. Reproductive BioMedicine Online. 2010 Sep;21(3):338–43.

31. Ng EHY, Li RHW, Chen L, Lan VTN, Tuong HM, Quan S. A randomized double blind comparison of atosiban in patients undergoing IVF treatment. Human Reproduction. 2014 Jun 26;29(12):2687–94.

32. Song Z-R, Zhao X-H, Bai X-H, Lu Y-H, Zhang H-J, Wang Y-X, et al. Application of oxytocin antagonists in thaw embryo transfer. Zhonghua Fu Chan Ke Za Zhi. 2013;48(9):667–70.

33. Visnova H, Tournaye HJ, Humberstone A, Terrill P, Macgregor L, Loumaye E. A placebo-controlled, randomized, double-blind, phase 3 study assessing ongoing pregnancy rates after single oral administration of a novel oxytocin receptor antagonist, nolasiban, prior to single embryo transfer. Fertility and Sterility. 2018 Sep 1;110(4):e45.

34. Yuan C, Song H, Fan L, Su S, Dong B. The Effect of Atosiban on Patients With Difficult Embryo Transfers Undergoing In Vitro Fertilization–Embryo Transfer. Reproductive Sciences. 2019 Dec 1;26(12):1613–7.

35. Amarin ZO, Obeidat BR. Bed rest versus free mobilisation following embryo transfer: a prospective randomised study. BJOG: An International Journal of Obstetrics & Gynaecology. 2004 Nov 1;111(11):1273–6.

36. Botta G, Grudzinskas G. Is a prolonged bed rest following embryo transfer useful? Human Reproduction [Internet]. 1997 Nov 1 [cited 2021 Aug 9];12(11):2489–92. Available from: https://academic.oup.com/humrep/article/12/11/2489/664900

37. Gaikwad S, Garrido N, Cobo A, Pellicer A, Remohi J. Bed rest after embryo transfer negatively affects in vitro fertilization: a randomized controlled clinical trial. Fertility and Sterility. 2013 Sep 1;100(3):729-735.e2.

38. Malhotra N, Sarkar P. Pregnancy outcome after bed rest versus early ambulation following embryo transfer during IVF/ICSI cycles-a randomised controlled study. Fertility and Sterility. 2019 Sep 1;112(3):e146.

39. Purcell KJ, Schembri M, Telles TL, Fujimoto VY, Cedars MI. Bed rest after embryo transfer: a randomized controlled trial. Fertility and Sterility. 2007 Jun 1;87(6):1322–6.

40. Rezábek K, Koryntova D, Zivny J. Does bedrest after embryo transfer cause a worse outcome in in vitro fertilization? Ceska Gynekologie. 2001;66(3):175–8.

41. Arvas A, Karacan M, Ulug M, Cebi Z, Berberoglugil M, Batukan M, et al. A 20-second wait or immediate withdrawal of the catheter following ultrasound-guided blastocyst transfer. Fertility and Sterility. 2014 Sep 1;102(3):e30.

42. Devranoǧlu B, Özdamar Ö, Caklroǧlu Y, Küçükbaş M, Eken MK, Doǧer E. The Timing of Embryo Transfer Catheter Removal: Should It be Delayed or Done Immediately? A Prospective Randomized Trial. Gynecologic and Obstetric Investigation. 2018 Jan 1;83(1):29–34.

43. Martínez F, Coroleu B, Parriego M, Carreras O, Belil I, Parera N, et al. Ultrasound-guided embryo transfer: immediate withdrawal of the catheter versus a 30 second wait. Human Reproduction. 2001 May 1;16(5):871–4.

44. Berkkanoglu M, Isikoglu M, Seleker M, Ozgur K. Flushing the endometrium prior to the embryo transfer does not affect the pregnancy rate. Reproductive BioMedicine Online. 2006 Jan 1;13(2):268–71.

45. Glass KB, Green CA, Fluker MR, Schoolcraft WB, McNamee PI, Meldrum DR. Multicenter Randomized Controlled Trial of Cervical Irrigation at the Time of Embryo Transfer. Fertility and Sterility. 2000 Sep 1;74(3):S31.

46. Moini A, Kiani K, Bahmanabadi A, Akhoond M, Akhlaghi A. Improvement in pregnancy rate by removal of cervical discharge prior to embryo transfer in ICSI cycles: A randomised clinical trial. Australian and New Zealand Journal of Obstetrics and Gynaecology. 2011 Aug;51(4):315–20.

47. Ruhlman C, Bisoli C, Terrado G, Rolla ED, Nicholson RE, Gnocchi D. The inefficacy of cervical mucus aspiration prior to embryo transfer: A prospective randomized trial [abstract]. ASRM/CFAS Conjoint Annual Meeting. Fertility and Sterility. 1999;71(1):154.

48. Soroka D, Wells G, Kotaba DD. Does the aspiration of cervical mucus prior to embryo transfer in women undergoing in vitro fertilization and embryo transfer improve pregnancy rates? Fertility and Sterility. 1999;72(S102).

49. Visschers B, Bots R, Peeters M, Mol B, van Dessel H. Removal of cervical mucus: effect on pregnancy rates in IVF/ICSI. RBM Online. 2007;15(3):310–5.

50. Al-Shawaf T, Dave R, Harper J, Linehan D, Riley P, Craft I. Transfer of embryos into the uterus: How much do technical factors affect pregnancy rates? Journal of Assisted Reproduction and Genetics. 1993 Jan;10(1):31–6.

51. Allahbadia GN, Kadam K, Gandhi G, Arora S, Valliappan JB, Joshi A, et al. Embryo transfer using the SureView catheter-beacon in the womb. Fertility and Sterility. 2010 Jan 15;93(2):344–50.

52. Gilberto Almodin C, Cibele V, Câmara M, Gonçalves P, Pereira C, Lopes Paixão C, et al. Estudo comparativo entre dois cateteres diferentes usados para transferência de embriões Comparative study between different catheters for embryo transfer.

53. Amorcho B, Gomez E, Pontes L, Campos I, Landeras J, Munoz M, et al. Does the selection of catheter for embryo transfer affect the success rate of an ART unit? Human Reproduction. 1999;14(Suppl_3):205–205.

54. Baris D, Ata AB, Isiklar A, Balaban B, Urman B. Prospective randomized comparison of Wallace and Labotect embryo transfer catheters. BioMedicine Online. 2007;14(4):471–6.

55. Boone WR, Johnson JE, Blackhurst DM, Crane MM. Short Communication: Cook Versus EdwardsWallace: Are There Differences in Flexible Catheters? Journal of Assisted Reproduction and Genetics 2001 18:1. 2001;18(1):15–7.

56. Candan Z, Avcil F, Ozdenn H, Uslu H, Karamn Y. Comparing the performance of the Cook and the Prodimed embryo transfer catheters. Reproductive Biomedicine Online. 2014;28:S15.

57. Coroleu B, Barri PN, Carreras O, Belil I, Buxaderas R, Veiga A, et al. Effect of using an echogenic catheter for ultrasound-guided embryo transfer in an IVF programme: A prospective, randomized, controlled study. Human Reproduction. 2006 Jul 1;21(7):1809–15.

58. Curfs M, Cleine J, van Kamp A, Kruse-Blankestijn M, Hondelink M, Leerentveld R. Comparison of the Wallace versus TDT embryo-transfer catheter: a prospective, randomized study. Reprod Biomed Online. 2001;3(1).

59. El-Shawarby SA, Ravhon A, Skull J, Ellenbogen A, Trew G, Lavery S. A prospective randomized controlled trial of Wallace and Rocket embryo transfer catheters. Reproductive BioMedicine Online. 2008 Jan 1;17(4):549–52.

60. Foutouh I, Youssef M, Tolba M, Rushdi M, Nakieb A, Meguid W. Does embryo transfer catheter type affect pregnancy rate? Middle East Fertil Soc. 20003;8:154–8.

61. Ghazzawi IM, Al-Hasani S, Karaki R, Souso S. Transfer technique and catheter choice influence the incidence of transcervical embryo expulsion and the outcome of IVF. Human Reproduction. 1999 Mar 1;14(3):677–82.

62. Grunert GM, Dunn RC, Valdes CT, Wun CC, Wun WSA. Comparison of Wallace, Frydman DT and Cook embryo transfer catheter for IVF: a prospective randomised study [abstract]. Fertility and Sterility. 1998;70(S120).

63. Karande V, Hazlett D, Vietzke M, Gleicher N. A prospective randomized comparison of the Wallace catheter and the Cook Echo-Tip® catheter for ultrasound-guided embryo transfer. Fertility and Sterility. 2002 Apr 1;77(4):826–30.

64. Lavery S, Ravhon A, Skull J, Ellenbogen A, Winston R. A prospective randomized controlled trial of Wallace and Rocket embryo transfer catheters in an IVF–embryo transfer programme [abstract]. Human Reproduction. 2001;16(Suppl 1):124.

65. Levi-Setti PE, Albani E, Baggiani AM, Zannoni E, Colombo G, Liprandi V. Prospective randomized study comparing two soft catheters for embryo transfer. Fertility and Sterility. 2002 Sep 1;78:S234–5.

66. Mayer JF, Nehchiri F, Jones EC, Weedon VM, Kalin HL, Lanzendorf SE, et al. Prospective randomized analysis of the impact of two different transfer catheters on clinical pregnancy rates [abstract]. Fertility and Sterility. 1999;1:144–5.

67. McDonald JA, Norman RJ. A randomized controlled trial of a soft double lumen embryo transfer catheter versus a firm single lumen catheter: significant improvements in pregnancy rates. Human Reproduction. 2002 Jun 1;17(6):1502–6.

68. McIlveen M, Lok FD, Pritchard J, Lashen H. Modern embryo transfer catheters and pregnancy outcome: A prospective randomized trial. Fertility and Sterility. 2005 Oct;84(4):996–1000.

69. Meriano J, Weissman A, Greenblatt EM, Ward S, Casper RF. The choice of embryo transfer catheter affects embryo implantation after IVF. Fertility and Sterility. 2000 Oct;74(4):678–82.

70. Mortimer ST, Fluker M, Yuzpe A. Effect of embryo transfer catheter on implantation rates. Fertility and Sterility. 2002 Sep 1;78:S17–8.

71. Ocal P, Cepni I, Idil MH, Salihoglu F, Irez T, Aksu F. Appropriate embryo transfer technique improves the pregnancy rate in human in vitro fertilization. Middle East Fertility Society Journal. 2003;8(1):65–8.

72. Perin PM, Neves P A, Maluf M. The Influence of Two Different Transfer Catheters on the Pregnancy Rate in a Human In Vitro Fertilization/Embryo Transfer Program. Fertility and Sterility. 1997;1997(1001):220.

73. Rhodes TL, Higdon HL, Boone WR. Comparison of pregnancy rates for two embryo-transfer catheters. Fertility and Sterility. 2007 Feb;87(2):411–6.

74. Ruhlmann C, Gnocchi DC, Cattaneo AR, Molina LG, Rivadeneira LR, Tessari L, et al. Embryo Transfer Catheters: Softer is easier. Jornal Brasileiro de Reproducao Assistida. 2015;19(4):204–9.

75. Saldeen P, Abou-Setta AM, Bergh T, Sundström P, Holte J. A prospective randomized controlled trial comparing two embryo transfer catheters in an ART program. Fertility and Sterility. 2008 Sep;90(3):599–603.

76. Talwar P, Naredi N, Sandeep K, Joneja G, Duggal B. Does catheter choice during embryo transfer alter the pregnancy rate? Medical Journal, Armed Forces India. 2011;67(4):311.

77. van Weering HGI, Schats R, McDonnell J, Vink JM, Vermeiden JPW, Hompes PGA. The impact of the embryo transfer catheter on the pregnancy rate in IVF. Human Reproduction. 2002 Mar 1;17(3):666–70.

78. Wisanto A, Janssens R, Deschacht J, Camus M, Devroey P, van Steirteghem AC. Performance of different embryo transfer catheters in a human in vitro fertilization program. Fertility and Sterility. 1989;52(1):79–84.

79. Yao Z, Vansteelandt S, van der Elst J, Coetsier T, Dhont M, de Sutter P. The efficacy of the embryo transfer catheter in IVF and ICSI is operator-dependent: a randomized clinical trial. Human Reproduction. 2009 Apr 1;24(4):880–7.

80. Lewin A, Schenker JG, Avrech O, Shapira S, Safran A, Friedler S. The role of uterine straightening by passive bladder distension before embryo transfer in IVF cycles. Journal of Assisted Reproduction and Genetics 1997 14:1. 1997;14(1):32–4.

81. Lorusso F, Depalo R, Bettocchi S, Vacca M, Vimercati A, Selvaggi L. Outcome of in vitro fertilization after transabdominal ultrasound–assisted embryo transfer with a full or empty bladder. Fertility and Sterility. 2005 Oct 1;84(4):1046–8.

82. Mitchell JD, Wardle PG, Foster PA, Hull MGR. Effect of bladder filling on embryo transfer. Journal of In Vitro Fertilization and Embryo Transfer. 1989 Aug;6(4):263–5.

83. Arefi S, Fazeli E, Esfahani M, Borhani N, Yamini N, Hosseini A, et al. Granulocyte-colony stimulating factor may improve pregnancy outcome in patients with history of unexplained recurrent implantation failure: An RCT. Int J Reprod BioMed. 2018;16(5):299–304.

84. Obidniak D, Gzgzyan A, Dzhemlikhanova L, Feoktistov A. Effect of colony-stimulating growth factor on outcome of frozen-thawed embryo transfer in patients with repeated implantation failure. Fertility and Sterility. 2016 Sep 1;106(3):e134–5.

85. Singh R, Singh M, Jindal A, Jindal P. A prospective randomized controlled study (RCT) of intra-uterine administration of granulocyte colony-stimulating factor (G-CSF) before embryo-transfer on resistant thin endometrium in IVF cycles. Human Reproduction. 2015;30(1):120.

86. Singh R, Singh M. P-296  RCT of intra-uterine administration or subcutaniousinjection of GCSF (granulocyte colony-stimulating factor) beforeembryo-transfer on resistant thin endometrium in IVF. Human Reproduction. 2018 Jul 1;33(suppl_1):i1–541.

87. Aaleyasin A, Aghahosseini M, Rashidi M, Safdarian L, Sarvi F, Najmi Z, et al. In vitro fertilization outcome following embryo transfer with or without preinstillation of human chorionic gonadotropin into the uterine cavity: A randomized controlled trial. Gynecologic and Obstetric Investigation. 2015 Apr 6;79(3):201–5.

88. Cambiaghi AS, Leao RBF, Alvarez AV, Nascimento PF. Intrauterine injection of human chorionic gonadotropin before embryo transfer may improve clinical pregnancy and implantation rates in blastocysts transfers. Fertility and Sterility. 2013 Sep 1;100(3):S121.

89. Dehghani Firouzabadi R, Janati S, Razi MH. The effect of intrauterine human chorionic gonadotropin injection before embryo transfer on the implantation and pregnancy rate in infertile patients: A randomized clinical trial. International Journal of Reproductive Biomedicine. 2016 Oct 1;14(10):657.

90. Eskandar M, Al-Emain M, Atwan Y, Bakar S. Does intrauterine injection of human chorionic gonadotropin before embryo transfer improve the pregnancy rate in vitro fertilization/ intracytoplasmic sperm injection (IVFICSI) cycles? A prospective randomized controlled trial. Reproductive Sciences. 2016 Mar;23(1):102A.

91. Hafezi M, Madani T, Arabipoor A, Zolfaghari Z, Sadeghi M, Ramezanali F. The effect of intrauterine human chorionic gonadotropin flushing on live birth rate after vitrified-warmed embryo transfer in programmed cycles: a randomized clinical trial. Archives of Gynecology and Obstetrics 2018 297:6. 2018 Apr 6;297(6):1571–6.

92. Hong KH, Forman EJ, Werner MD, Upham KM, Gumeny CL, Winslow AD, et al. Endometrial infusion of human chorionic gonadotropin at the time of blastocyst embryo transfer does not impact clinical outcomes: A randomized, double-blind, placebo-controlled trial. Fertility and Sterility. 2014 Dec 1;102(6):1591-1595.e2.

93. Hosseini RS, Farzad L, Abdollahi S, Nouri M, Ghasemzadeh A, Hamdi K, et al. Effect of intrauterine injection of human chorionic gonadotropin before frozen-thawed embryo transfer on implantation and clinical pregnancy rate: A randomized controlled trial. International Journal of Women’s Health and Reproduction Sciences. 2016 Oct 1;4(4):200–3.

94. Laokirkkiat P, Thanaboonyawat I, Boonsuk S, Petyim S, Prechapanich J, Choavaratana R. Increased implantation rate after intrauterine infusion of a small volume of human chorionic gonadotropin at the time of embryo transfer: a randomized, double-blind controlled study. Archives of Gynecology and Obstetrics. 2019 Jan 14;299(1):267–75.

95. Leao R, Cambiaghi A, Leao B, Alvarez A, Figueiredo P. Intrauterine injection of human chorionic gonadotropin before embryo transfer may improve the pregnancy rates in in vitro fertilization cycles of patients with repeated implantation failures. In: Proceedings of the 5th IVI International Congress. Seville, Spain; 2013.

96. Mansour R, Tawab N, Kamal O, El-Faissal Y, Serour A, Aboulghar M, et al. Intrauterine injection of human chorionic gonadotropin before embryo transfer significantly improves the implantation and pregnancy rates in in vitro fertilization/intracytoplasmic sperm injection: A prospective randomized study. Fertility and Sterility. 2011;96(6).

97. Mostajeran, Godazandeh F, Ahmadi SM, Movahedi M, Jabalamelian SA. Effect of intrauterine injection of human chorionic gonadotropin before embryo transfer on pregnancy rate: A prospective randomized study. Journal of Research in Medical Sciences. 2017;22(1):6.

98. Santibañez Á, García J, Pashkova O, Colín O, Castellanos G, Sánchez AP, et al. Effect of intrauterine injection of human chorionic gonadotropin before embryo transfer on clinical pregnancy rates from in vitro fertilisation cycles: A prospective study. Reproductive Biology and Endocrinology. 2014 Jan 29;12(1).

99. Singh R, Singh M. Intra‐uterine administration of human chorionic gonadotrophin (hCG) before embryo transfer in recurrent implantation failure (RIF) patients improves implantation and pregnancy rates in IVF‐ICSI cycles. Human Reproduction. 2014;29(1).

100. Wang M, Deng H, Ye H. Intrauterine injection of human chorionic gonadotropin improves pregnancy outcome in patients with repeated implantation failure in frozen-thawed embryo transfer. Journal of Central South University (Medical Sciences). 2019 Nov 1;44(11):1247–51.

101. Wirleitner B, Schuff M, Vanderzwalmen P, Stecher A, Okhowat J, Hradecký L, et al. Intrauterine administration of human chorionic gonadotropin does not improve pregnancy and life birth rates independently of blastocyst quality: A randomised prospective study. Reproductive Biology and Endocrinology. 2015 Jul 4;13(1).

102. Wirleitner B, Schuff M, Vanderzwalmen P, Stecher A. O-182 The usefulness of intrauterine hCG administration prior to blastocyst transfer in IVF-patients ≥38 years. Human Reproduction. 2015 Jun 1;30(suppl_1):i1–501.

103. Zarei A, Parsanezhad ME, Younesi M, Alborzi S, Zolghadri J, Samsami A, et al. Intrauterine administration of recombinant human chorionic gonadotropin before embryo transfer on outcome of in vitro fertilization/ intracytoplasmic sperm injection: A randomized clinical trial. Iranian Journal of Reproductive Medicine. 2014 Jan 1;12(1):1.

104. Balaban B, Urman B, Yakin K, Isiklar A, Kilic Y, Aksoy S. High pregnancy and implantation rates can be achieved in blastocyst transfers using hyaluronan enriched culture and transfer medium. Fertility and Sterility. 2004 Sep 1;82:S221.

105. Dittmann-Műller X, Zollner K, Zollner U. Prospective randomised clinical trial about the efficacy of a human embryo transfer medium (EmbryoGlue(R)). Human Reproduction. 2009;24:167.

106. Fancsovits P, Murber A, Gilan Z, Rigo J, Urbancsek J. Effect of hyaluronan containing transfer media on pregnancy and implantation rates in human IVF-ET cycles. A prospective randomized study. Human Reproduction. 2011;26(1).

107. Fancsovits P, Lehner A, Murber A, Kaszas Z, Rigo J, Urbancsek J. Effect of hyaluronan-enriched embryo transfer medium on IVF outcome: a prospective randomized clinical trial. Archives of Gynecology and Obstetrics. 2015 Nov 15;291(5):1173–9.

108. Fasano G, Antonacci R, Biramane J, Mbongolo G, Nguyen Thi M, Vanhelleputte C. Clinical outcomes after use of embryo-glue as a human embryo transfer (ET) medium in warming cycles. Human Reproduction. 2016;31:204–5.

109. Feichtinger W, Strohmer H, Radner KM, Goldin M. The use of fibrin sealant for embryo transfer: development and clinical studies. Human Reproduction. 1992 Jul 1;7(6):890–3.

110. Friedler S, Raziel A, Schachter M, Strassburger D, Kasterstein E, Komarovsky D. Efficacy of hyaluronan-enriched embryo transfer medium in patients with repeated IVF-ET failures. Human Reproduction. 2005;20(1):159.

111. Friedler S, Schachter M, Strassburger D, Esther K, Ron El R, Raziel A. A randomized clinical trial comparing recombinant hyaluronan/recombinant albumin versus human tubal fluid for cleavage stage embryo transfer in patients with multiple IVF-embryo transfer failure. Human Reproduction. 2007 Sep 1;22(9):2444–8.

112. Kandari S. Time lapse selected elective single embryo transfer in hyaluronan enriched transfer medium in PCOS improves live birth rates compared to use of conventional embryo transfer media. a possible alternative to freeze-all cycles in PCOS. Fertility and Sterility ASRM. 2019;11(3):47–8.

113. Korosec S, Virant-Klun I, Tomazevic T, Zech NH, Meden-Vrtovec H. Single fresh and frozen–thawed blastocyst transfer using hyaluronan-rich transfer medium. 2007 [cited 2021 Aug 10];15(6):701–7. Available from: www.rbmonline.com/Article/3065

114. Mahani IM, Davar R. Hyaluronic acid versus albumin in human embryo transfer medium. Eastern Mediterranean Health Journal. 2007;13(4):876–80.

115. Morbeck D. Prospective randomized clinical trial of novel implantation promoting medium (EmbryoGlue) to improve IVF success rates. 2007.

116. Perez O, Adriaanse H, Tilley B, Navarrete G, Lay L, Little LM, et al. The effect of extended blastocyst exposure of hyaluronan enriched transfer media on implantation rate in frozen embryo transfers. Fertility and Sterility. 2019 Sep;112(3):e438.

117. Ravhon A, Nahum H, Weissman A, Biran G, Umansky N, Levran D. Embryo Transfer in Hyaluronan Enriched Transfer Medium Does Not Improve Pregnancy Rate in IVF Treatment. Fertility and Sterility. 2005 Sep 1;84:S376–7.

118. Schoolcraft W, Lane M, Stevens J, Gardner DK. Increased hyaluronan concentration in the embryo transfer medium results in a significant increase in human embryo implantation rate. Fertility and Sterility. 2002 Sep 1;78:S5.

119. Simon A, Safran A, Revel A, Aizenman E, Reubinoff B, Porat-Katz A, et al. Hyaluronic acid can successfully replace albumin as the sole macromolecule in a human embryo transfer medium. Fertility and Sterility. 2003 Jun 1;79(6):1434–8.

120. Tomari H, Honjou K, Kunitake K, Nishimura K, Hidaka N, Nagata Y. Effect of embryo glue transfer medium during fresh and frozen-thawed embryo transfer. Fertility and Sterility. 2014 Sep 1;102(3):e313.

121. Urman B, Yakin K, Ata B, Isiklar A, Balaban B. Effect of hyaluronan-enriched transfer medium on implantation and pregnancy rates after day 3 and day 5 embryo transfers: a prospective randomized study. Fertility and Sterility. 2008 Sep;90(3):604–12.

122. Valojerdi MR, Karimian L, Yazdi PE, Gilani MAS, Madani T, Baghestani AR. Efficacy of a human embryo transfer medium: A prospective, randomized clinical trial study. Journal of Assisted Reproduction and Genetics. 2006 May;23(5):207–12.

123. Walker DL, Thornhill AR, Allemand MC, Tatpati LL, Wentworth MA, Tummon IS. A Randomized, Controlled, Double Blinded Trial of EmbryoGlue® in Frozen Embryo Transfer Cycles: An Interim Analysis. Fertility and Sterility. 2005 Sep 1;84:S415–6.

124. Yakin K, Balaban B, Isiklar A, Bozdag H, Urman B. Improved clinical outcome in frozen-thawed embryo transfers with the use of hyaluronan-enriched medium. Human Reproduction. 2004;19(1):89.

125. Yung SSF, Lai SF, Lam MT, Lui EMW, Ko JKY, Li HWR, et al. Hyaluronic acid–enriched transfer medium for frozen embryo transfer: a randomized, double-blind, controlled trial. Fertility and Sterility. 2021;0(0).

126. Bernabeu R, Roca M, Torres A, Ten J. Indomethacin effect on implantation rates in oocyte recipients. Human Reproduction. 2006;21(2):364–9.

127. Prato LD, Borini A. Effect of piroxicam administration before embryo transfer on IVF outcome: a randomized controlled trial. Reproductive BioMedicine Online. 2009 Oct 1;19(4):604–9.

128. Duvan CI, Ozmen B, Satıroglu H, Atabekoglu CS, Berker B. Does addition of low-dose aspirin and/or steroid as a standard treatment in nonselected intracytoplasmic sperm injection cycles improve in vitro fertilization success? A randomized, prospective, placebo-controlled study. Journal of Assisted Reproduction and Genetics 2005 23:1. 2006 Mar 4;23(1):15–21.

129. Fekih M, Chachia S, Zarrouk W, Khairi H. Effect of ibuprophen administration before embryo transfer on IVF outcome: a randomized controlled trial. Fertility and Sterility. 2013;100:S122.

130. Dehghani Firouzabadi R, Ghandi S, Tayebi N. Effect of administration of single dose piroxicam before embryo transfer on implantation and pregnancy rates in IVF cycles. Journal of Biological Sciences. 2007 Jan 1;7(1):123–6.

131. Moon HS, Park SH, Lee JO, Kim KS, Joo BS. Treatment with piroxicam before embryo transfer increases the pregnancy rate after in vitro fertilization and embryo transfer. Fertility and Sterility. 2004 Oct;82(4):816–20.

132. Zarei A, Homayoon N, Hessami K, Hashemi A, Davoodi S, Razavi B, et al. Effect of piroxicam administration on outcome of frozen–thawed embryo transfer: A randomized, double-blinded, placebo-controlled trial. Journal of Obstetrics and Gynaecology Research. 2021 Jan 1;47(1):296–301.

133. Bjuresten K, Hreinsson JG, Fridström M, Rosenlund B, Ek I, Hovatta O. Embryo transfer by midwife or gynecologist: A prospective randomized study. Acta Obstetricia et Gynecologica Scandinavica. 2003 May 1;82(5):462–6.

134. Rinaldi L, Floccari A, Selman H. Ultrasound guidance of embryo transfer: A role for midwife. Sexual & Reproductive Healthcare. 2014 Jun 1;5(2):47–9.

135. Aba YA, Avci D, Guzel Y, Ozcelik SK, Gurtekin B. Effect of music therapy on the anxiety levels and pregnancy rate of women undergoing in vitro fertilization-embryo transfer: A randomized controlled trial. Applied Nursing Research. 2017 Aug 1;36:19–24.

136. Gavrizi SZ, Skillern A. Does massage therapy immediately prior to embryo transfer improve clinical pregnancy rate in IVF-PGT-A (in-vitro fertilization-preimplantation genetic testing for aneuploidy) cycles? Fertility and Sterility. 2019 Sep;112(3):e184–5.

137. MacLennan AH, Kerin JFP, Kirby C, Grant P, Warnes GM, Cox LW, et al. The Effect of Porcine Relaxin Vaginally Applied at Human Embryo Transfer in an In Vitro Fertilization Programme. Australian and New Zealand Journal of Obstetrics and Gynaecology. 1985;25(1):68–71.

138. Murphy EM, Nichols J, Somkuti SG, Sobel M, Braverman A, Barmat LI. Randomized Trial of Harp Therapy During In Vitro Fertilization-Embryo Transfer. Journal of Evidence-Based Complementary and Alternative Medicine. 2014;19(2):93–8.

139. Shaker AG, Fleming R, Jamieson ME, Yates RWS, Coutts JRT. Infertility: Assessments of embryo transfer after in-vitro fertilization: effects of glyceryl trinitrate. Human Reproduction. 1993 Sep 1;8(9):1426–8.

140. Stocker LJ, Hardingham KL, Cheong YC. A randomized controlled trial assessing whether listening to music at time of embryo transfer effects anxiety levels. Gynecology and Obstetrics. 2016;6(9):1–6.

141. Aflatoonian A, Ghandi S, Tabibnejad N. The Effect of Intercourse around Embryo Transfer on Pregnancy Rate in Assisted Reproductive Technology Cycles. Royan Institue International Journal of Fertility and Sterility. 2(4).

142. Karimian L, Naghibi ZH, Yazdi PE, Moini A, Valojerdi MR, Akhondi MM, et al. 147 THE EFFECT OF INTERCOURSE ON b HCG LEVEL AND PREGNANCY OUTCOME AFTER IVF CYCLES RETROSPECTIVE EVALUATION OF MECHANICAL AND LASER ASSISTED HATCHING EFFICIENCY IN TWO DIFFERENT AGE GROUPS. Reproductive BioMedicine Online. 2010;20:S65.

143. Coroleu B, Barri PN, Carreras O, Martínez F, Parriego M, Hereter L, et al. The influence of the depth of embryo replacement into the uterine cavity on implantation rates after IVF: a controlled, ultrasound-guided study. Human Reproduction. 2002 Feb 1;17(2):341–6.

144. Franco JG, Martins AMVC, Baruffi RLR, Mauri AL, Petersen CG, Felipe V, et al. Best site for embryo transfer: the upper or lower half of endometrial cavity? Human Reproduction. 2004 Aug 1;19(8):1785–90.

145. Nazari A, Askari HA, Check JH, O’Shaughnessy A. Embryo transfer technique as a cause of ectopic pregnancy in in vitro fertilization. Fertility and Sterility. 1993 Nov 1;60(5):919–21.

146. Abdelmassih VG, Abdelmassih ST, Nagy ZP, Abdelmassih R, Balmaceda J. The effect of ultrasound (US) guided embryo transfer (ET) and the choice of catheter on the outcome of IVF. Fertility and Sterility. 2001 Sep 1;76(3):S88–9.

147. Ammar A, Mousa K, Noha Hamed Rabei N, Galal A. Effect of ultrasound guided embryo transfer on pregnancy rates. New York Science Journal. 2013;6(1):79–83.

148. Azmy O, Taha T, Bibars M, Refaat M. Ultrasound guided versus blind touch embryo transfer: the Egyptian experience. In: Proceedings of the 15th World Conference of IVF and 4th Congress of IVM. 2009. p. 9–12.

149. Bar-Hava I, Meltzer S, Rabinson J, Ayash I, Sega S, Tur-Kaspa I. Ultrasound guided versus blind tactile embryo transfer: a prospective randomised study. Human Reproduction. 2003;18(1).

150. Bodri D, Colodrón M, García D, Obradors A, Vernaeve V, Coll O. Transvaginal versus transabdominal ultrasound guidance for embryo transfer in donor oocyte recipients: A randomized clinical trial. Fertility and Sterility. 2011;95(7).

151. Chen S-C, Lai T-H, Lee F-K. The influence of abdominal ultrasound-guided embryo transfer on pregnancy rate: a preliminary report. Fertility and Sterility. 2007 May 1;87(5):1235–7.

152. Coroleu B, Carreras O, Veiga A, Martell A, Martinez F, Belil I, et al. Embryo transfer under ultrasound guidance improves pregnancy rates after in-vitro fertilization. Human Reproduction. 2000 Mar 1;15(3):616–20.

153. Coroleu B, Barri PN, Carreras O, Martínez F, Veiga A, Balasch J. The usefulness of ultrasound guidance in frozen–thawed embryo transfer: a prospective randomized clinical trial. Human Reproduction. 2002 Nov 1;17(11):2885–90.

154. Dalal R, Pai H, Palshetkar N. Comparison of transabdominal (TA) versus transvaginally (TV) guided embryo transfer in oocyte recipient cycles: A prospective randomised trial. BJOG: An International Journal of Obstetrics and Gynaecolog. 2014;121:82.

155. Davar R, Ghandi S, Tayebi N. Does transabdominal ultrasound-guided embryo transfer improve pregnancy rates in ART cycles? Iranian Journal of Reproductive Medicine. 2007;5(3):95–8.

156. de Camargo Martins AMV, Baruffi RLR, Mauri AL, Petersen C, Oliveira JBA, Contart P, et al. Ultrasound guidance is not necessary during easy embryo transfers. Journal of assisted reproduction and genetics. 2004 Dec;21(12):421–5.

157. Deep JP, Li Y, Liu H. The Effect of Embryo Transfer on IVF Treatment: Comparing the Trans-Vaginal Versus Transabdominal Ultrasound Approach. Journal of Minimally Invasive Gynecology. 2013 Nov 1;20(6):S42–3.

158. Drakeley AJ, Jorgensen A, Sklavounos J, Aust T, Gazvani R, Williamson P, et al. A randomized controlled clinical trial of 2295 ultrasound-guided embryo transfers. Human Reproduction. 2008 May 1;23(5):1101–6.

159. Eskandar M, Abou-Setta AM, Almushait MA, El-Amin M, Mohmad SEY. Ultrasound guidance during embryo transfer: a prospective, single-operator, randomized, controlled trial. Fertility and Sterility. 2008 Oct 1;90(4):1187–90.

160. Garcı́a-Velasco JA, Martinez-Salazar J, Isaza V, Landazabal A, Requena A, Simón C. Does ultrasound guidance at embryo transfers improve pregnancy rates in oocyte recipients? Fertility and Sterility. 2001 Sep 1;76(3):S75–6.

161. García-Velasco J, Isaza V, Martinez-Salazar J, Landazábal A, Requena A, Remohí J, et al. Transabdominal ultrasound-guided embryo transfer does not increase pregnancy rates in oocyte recipients. Fertility and sterility. 2002;78(3):534–9.

162. Hauzman E, Kohls G, Barrio A, Martinez-Salazar J, Iglesias C, Garcia-Velasco JA. Comparison of embryo transfer in egg donation recipients with transvaginal and transabdominal ultrasound: a randomized pilot study. Human Reproduction. 2013;28(241):undefined.

163. Kan AKS, Abdalla HI, Gafar AH, Nappi L, Ogunyemi BO, Thomas A, et al. Embryo transfer: ultrasound-guided versus clinical touch. Human Reproduction. 1999 May 1;14(5):1259–61.

164. Karavani G, Ben-Meir A, Shufaro Y, Hyman JH, Revel A. Transvaginal ultrasound to guide embryo transfer: a randomized controlled trial. Fertility and Sterility. 2017 May 1;107(5):1159–65.

165. Kosmas IP, Janssens R, de Munck L, al Turki H, van der Elst J, Tournaye H, et al. Ultrasound-guided embryo transfer does not offer any benefit in clinical outcome: a randomized controlled trial. Human Reproduction. 2007 May 1;22(5):1327–34.

166. Li R, Lu L, Hao G, Zhong K, Cai Z, Wang W. Abdominal ultrasound-guided embryo transfer improves clinical pregnancy rates after in vitro fertilization: Experiences from 330 clinical investigations. Journal of Assisted Reproduction and Genetics. 2005 Jan;22(1):3–8.

167. Maldonado LG, Ajzen SA, Busato WC, Iaconelli A, Bibancos M, Borges E. Impact of Previous Hysterossonometry on Embryo Transfer. Fertility and Sterility. 2005 Sep 1;84:S364.

168. Marconi G, Young E, Vilela M, Bello A, Young E, Sueldo C. Prospective randomized comparison of an ultrasound-guided embryo transfer versus a blind catheter placement. Fertility and Sterility. 2003 Sep 1;80:130.

169. Matorras R, Urquijo E, Mendoza R, Corcóstegui B, Expósito A, Rodríguez-Escudero F. Ultrasound-guided embryo transfer improves pregnancy rates and increases the frequency of easy transfers. Human reproduction (Oxford, England). 2002;17(7):1762–6.

170. Moraga-Sanchez R, Saucedo-De La Llata E, Batiza-Resendiz V, Santos-Haliscak R, Galache-Vega P. Ultrasound influence and 30 second wait in embryo transfer. Human Reproduction. 2004;19:127.

171. Porat N, Boehnlein LM, Schouweiler CM, Kang J, Lindheim SR. Interim analysis of a randomized clinical trial comparing abdominal versus transvaginal ultrasound-guided embryo transfer. Journal of Obstetrics and Gynaecology Research. 2010 Apr 1;36(2):384–92.

172. Prapas Y, Prapas N, Hatziparasidou A, Prapa S, Nijs M, Vanderzwalmen P, et al. The echoguide embryo transfer maximizes the IVF results. Acta Europaea fertilitatis. 1995 May 1;26(3):113–5.

173. Revelli A, Rovei V, Dalmasso P, Gennarelli G, Racca C, Evangelista F, et al. Large randomized trial comparing transabdominal ultrasound-guided embryo transfer with a technique based on uterine length measurement before embryo transfer. Ultrasound in Obstetrics & Gynecology. 2016 Sep 1;48(3):289–95.

174. Sallam HN, Agameya AF, Rahman AF, Ezzeldin F, Sallam AN. Ultrasound measurement of the uterocervical angle before embryo transfer: a prospective controlled study. Human Reproduction. 2002 Jul 1;17(7):1767–72.

175. Saravelos SH, Kong GWS, Chung JPW, Mak JSM, Chung CHS, Cheung LP, et al. A prospective randomized controlled trial of 3D versus 2D ultrasound-guided embryo transfer in women undergoing ART treatment. Human Reproduction. 2016 Oct 1;31(10):2255–60.

176. Tang OS, Ng EHY, So WWK, Ho PC. Ultrasound-guided embryo transfer: A prospective randomized controlled trial. Human Reproduction. 2001;16(11):2310–5.

177. Weissman A, Farhi J, Steinfeld Z, Mutsafi R, Nahum H, Levran D. A prospective, randomized study of ultrasound-guided embryo transfer. Fertility and Sterility. 2003 Sep 1;80:122.

178. Yayla Abide C, Ozkaya E, Sanverdi I, Bostancı Ergen E, Kurek Eken M, Devranoglu B, et al. Prospective Randomized Trial Comparing Embryo Transfers of Cases with and without Catheter Rotation during Its Withdrawal. Gynecologic and Obstetric Investigation. 2018 Jul 1;83(4):397–403.

179. Sigalos GΑ, Michalopoulos Y, Kastoras AG, Triantafyllidou O, Vlahos NF. Low versus high volume of culture medium during embryo transfer: a randomized clinical trial. Journal of Assisted Reproduction and Genetics 2017 35:4. 2017 Dec 12;35(4):693–9.

180. Catoire P, Delaunay L, Dannappel T, Baracchini D, Marcadet-Fredet S, Moreau O, et al. Hypnosis versus Diazepam for Embryo Transfer: A Randomized Controlled Study. American Journal of Clinical Hypnosis. 2013 Apr;55(4):378–86.

181. Ng KKL, Rozen G, Stewart T, Agresta F, Polyakov A. Does nifedipine improve outcomes of embryo transfer?: Interim analysis of a randomized, double blinded, placebo-controlled trial. Medicine. 2019 Jan 1;98(4):e14251.

182. Hannoun A, Zreik TG, Ghaziri G, Abu Musa A, Awwad J. Effect of powdered gloves, worn at the time of embryo transfer, on the pregnancy outcome of IVF cycles. Journal of Assisted Reproduction and Genetics. 2009 Jan;26(1):25–7.

183. Obidniak D, Gzgzyan A, Feoktistov A, Niauri D. Randomized controlled trial evaluating efficacy of autologous platelet -rich plasma therapy for patients with recurrent implantation failure. Fertility and Sterility. 2017 Sep 1;108(3):e370.

184. Mansour R. Minimizing embryo expulsion after embryo transfer: a randomized controlled study. Human Reproduction. 2005 Jan 1;20(1):170–4.

185. Amui J, Check JH, Brasile D. Speculum retention during embryo transfer does not improve pregnancy rates following embryo transfer - A randomized study. Clinical and Experimental Obstetrics and Gynecology [Internet]. 2011 [cited 2021 Aug 12];38(4):333–4. Available from: https://pubmed.ncbi.nlm.nih.gov/22268268/

186. Caanen MR, van der Houwen LE, Schats R, Vergouw CG, de Leeuw B, Lambers MJ, et al. Embryo Transfer with Controlled Injection Speed to Increase Pregnancy Rates: A Randomized Controlled Trial. Gynecologic and Obstetric Investigation. 2016 Sep 1;81(5):394–404.

187. Groutz A, Lessing JB, Wolf Y, Azem F, Yovel I, Amit A. Comparison of transmyometrial and transcervical embryo transfer in patients with previously failed in vitro fertilization-embryo transfer cycles and/or cervical stenosis. Fertility and Sterility. 1997 Jun 1;67(6):1073–6.

188. Abu-Musa A, Usta I, Nassar A, Hajami F, Hannoun A. Effect of 17α-hydroxyprogesterone caproate before embryo transfer on the outcome of in vitro fertilization and embryo transfer: a randomized trial. Fertility and Sterility. 2008 May;89(5):1098–102.
